# Precision multidimensional assay for high-throughput microRNA drug discovery

**DOI:** 10.1038/ncomms10709

**Published:** 2016-02-16

**Authors:** Benjamin Haefliger, Laura Prochazka, Bartolomeo Angelici, Yaakov Benenson

**Affiliations:** 1 Department of Biosystems Science and Engineering, Swiss Federal Institute of Technology (ETH Zürich), Mattenstrasse 26, 4058 Basel, Switzerland

## Abstract

Development of drug discovery assays that combine high content with throughput is challenging. Information-processing gene networks can address this challenge by integrating multiple potential targets of drug candidates' activities into a small number of informative readouts, reporting simultaneously on specific and non-specific effects. Here we show a family of networks implementing this concept in a cell-based drug discovery assay for miRNA drug targets. The networks comprise multiple modules reporting on specific effects towards an intended miRNA target, together with non-specific effects on gene expression, off-target miRNAs and RNA interference pathway. We validate the assays using known perturbations of on- and off-target miRNAs, and evaluate an ∼700 compound library in an automated screen with a follow-up on specific and non-specific hits. We further customize and validate assays for additional drug targets and non-specific inputs. Our study offers a novel framework for precision drug discovery assays applicable to diverse target families.

## Abstract (web summary)

Progress in drug discovery can be hampered by a limited exploration of chemical space and the difficulty in assessing the full range of drug candidates' effects on living cells. Here the authors describe a cell-based assay to distinguish between off-target and specific effects of candidate compounds targeting micro RNAs.

## Graphical abstract

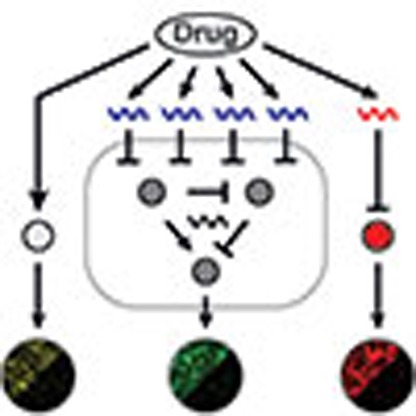

Progress in drug discovery is hampered by under-exploration of chemical space and by the difficulty in assessing the full range of drug candidates' effects on living cells. The former challenge is addressed by extending chemical space coverage, in part using synthetic pathways^1^,^2^ engineered using synthetic biology^3^,^4^,^5^,^6^,^7^,^8^,^9^,^10^,^11^,^12^ methods. The latter is partially solved with cell-based assays^13^ that allow evaluating drug action in a complex environment. Yet, these assays still generate candidate compounds that perform inadequately *in vivo* with respect to efficacy and toxicity^14^ in large part because many unwanted interactions^15^ pass undetected *in vitro*. Multiplex assays and serial testing^16^ have been proposed as a way to gauge off-target effects, yet increasing the number of measured parameters reduces assay throughput and makes it unsuitable for large library screens.

The weaknesses of cell-based assays are amplified with microRNAs (miRNAs) as drug targets. miRNA activity can be enhanced using miRNA mimics^17^ and inhibited with complementary RNA analogs^18^ or genetic sponges^19^. The search for small molecule miRNA modulators^20^,^21^ has relied on qPCR^22^ and genetic reporter^23^,^24^ assays, to produce a few candidate compounds^20^,^21^,^22^,^23^,^24^,^25^. However, multiple miRNA molecules can be easily targeted by the same compound due to similarities in nucleotide sequence and phosphoribose backbone, shared maturation pathway, and common pri-miRNA precursors^26^; therefore, the likelihood of side effects is high. Given that miRNAs are promising drug targets^27^,^28^ playing an important role in a large number of diseases^29^, there is a need for miRNA drug discovery tools that adequately address the high risk of non-specific effects with this target family.

We propose to address this challenge via rapid assessment of multiple off-target effects using intracellular genetic information-processing circuits^5^. These circuits are complex artificial regulatory networks that convert predefined biomolecular input cues into one or more gene products (outputs), according to a specified relationship. The circuits are implemented with multiple artificial genes that are delivered to and expressed in living cells. In the context of miRNA drug discovery, one can envision a network whose inputs are multiple potential off-target miRNAs and whose output is a single fluorescent reporter. This network would generate high output fluorescence when all its inputs are in their default (ground) state, corresponding to the lack of interference between the drug and the inputs, and low fluorescence otherwise, corresponding to at least one side effect. A network such as this can be formally described as an AND logic circuit, because the output of an AND circuit changes when any one of its inputs changes. Using formal notation and denoting a default state of input *X* (*X*=A, B, …) as input *X*^0^, the following equations hold:


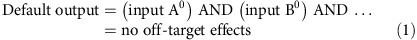



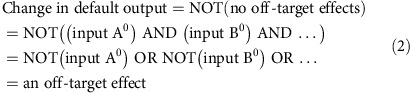


Equation (2) shows why a change in the AND gate output results from at least one off-target effect, without specifically identifying it. Adding inputs to the gate will expand the range of sampled off-target effects while keeping a single output without the need for multiplexing. Consequently, the validity and information content of the screen increases dramatically without sacrificing throughput.

Here we describe a novel cell-based assay that utilizes a genetic information-processing circuit to integrate and compress multiple miRNA inputs into a small number of fluorescent reporters to distinguish between off-target and specific effects of candidate compounds. Using an iterative simulation-aided design process, we implement the concept for miR-122, a promising drug target in liver cancer^30^ and hepatitis C^31^. We validate the assay in HuH-7 cells using miRNA mimics and inhibitors, then further adapt it for automated screening and test a library of ∼700 compounds. Finally, we reprogram and revalidate the assay to address additional miRNA drug targets and off-targets. Importantly, we show that compounds that would have been mistaken as specific hits with traditional methods are correctly identified as non-specific modulators. This study presents a precise yet high-throughput approach for miRNA drug discovery. The general concept is applicable to additional target families with appropriate modifications in the sensing and processing components.

## Results

### Basic concepts

**Figure 1:**
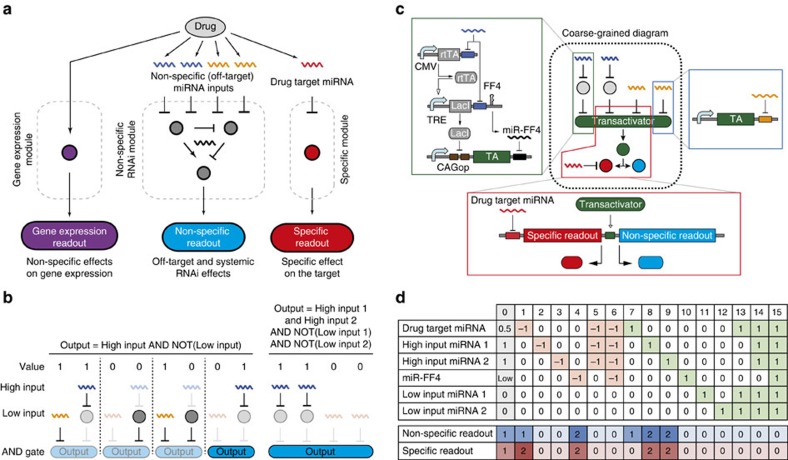
Assay design and validation strategy. (**a**) High-level representation of the screening assay modules. Module names, on- and off-target miRNA molecules and readouts are indicated. (**b**) Left: schematic representation of the possible input combinations and outcomes for a two-miRNA input AND gate. The values of 1 and 0 correspond to high and low miRNA expression, respectively. Note that the output is highly expressed only when high input is highly active, and low input is inactive. Squiggly lines are microRNAs, blunt arrows denote repression. Pale hue indicates low expression or activity. Logic gate that determines output activity is shown on top. Right: logic gate and corresponding input combination in the scaled-up four-input AND gate that results in high output expression. (**c**) Coarse-grained and detailed assay diagrams with four inputs for the non-specific module and one input for the specific module. Genetic implementation for each of the building blocks is shown in respective zoomed-in frames, with pointed arrows indicating activation, blunt arrows indicating repression and component names shown. (**d**) Validation perturbation table with 15 combinations. The ground state (column 0) describes the miRNA expression levels in the assay cell line furnished with the assay circuit, without perturbation. 1 stands for high and 0 for low expression, respectively. Drug target miRNA expression is shown as 0.5, indicating intermediate activity levels required to detect both up- and downregulation with drug candidates. For each perturbation column, −1 corresponds to miRNA inhibition, +1 is miRNA activation/overexpression relative to the ground state and 0 indicates no change. The anticipated expression of non-specific and specific readouts following a perturbation is shown in two bottom rows, with 1, 0 and 2 representing, respectively, the ground state, reduced and elevated readout. CAGop, CAG promoter followed by an intron with two LacO sites; CMV, cytomegalovirus immediate-early promoter; LacI, Lac repressor; rtTA, reverse Tet transactivator; TA, transactivator; TRE, TetR responsive element.

The assay circuit consists of three genetic modules reflecting distinct drug effects, including, respectively, global effects on gene expression (gene expression module), systemic effects on the RNAi pathway or off-target miRNAs (non-specific RNAi module) and the desired effect on the intended miRNA drug target (specific module) (Fig. 1a). The gene expression module reports on cell-wide changes in gene expression and cell viability via a constitutively expressed fluorescent protein. The same protein also serves as a transfection normalization control (see below). The non-specific RNAi module implements a genetic AND gate integrating multiple potential off-target miRNA inputs, into a single output reflected in the intensity of a fluorescent readout. To do so, we first place fully complementary, tandem binding sites for an miRNA input in the 3′-UTR of an mRNA coding for this output, to implement a ‘NOT (input)' logic^3^. A second miRNA input knocks down a repressor of that same output via fully complementary, tandem binding sites in the repressor's 3′-UTR, inverting miRNA inhibitory activity. This results in a logic gate that facilitates high output expression when the second miRNA is highly expressed (high input) and the first miRNA is expressed at low levels (low input): ‘high input AND NOT (low input)' (Fig. 1b, left). Additional high and low miRNA inputs can be added to scale up the gate (Fig. 1b, right). This gate structure suits our purpose because non-specific downregulation of miRNAs will reduce highly expressed miRNA inputs, and non-specific upregulation will elevate low miRNA inputs, with either scenario altering the output. Previous work^8^ showed how to improve the inversion of high inputs with additional genetic elements (Fig. 1c, left). We note that the module contains multiple gene expression components and it might also be sensitive to global changes in gene expression.

The output of the AND gate can be a fluorescent reporter; however, following our previously shown strategy for robust integration of multiple modules using a shared transactivator ‘knot' component^32^, we use a transactivator as the immediate AND gate output. This transactivator controls two fluorescent proteins. The first mirrors transactivator expression and serves as the non-specific assay readout; the second protein is furnished in addition with the binding sequence of the intended drug target miRNA in its 3′-UTR. Increased miRNA activity leads to a reduction in this reporter level and *vice versa* (Fig. 1c, bottom), relative to the non-specific readout. This reporter's expression (normalized to the non-specific readout) constitutes the specific assay readout.

### Validation strategy

We established a set of positive and negative controls to validate the assay modules. Ideally, controls should be chemical counterparts of candidate compounds^33^. We sought small molecule compounds with proven anti miR-122 activity, as well as those targeting multiple miRNAs or the RNAi pathway. Due to the late emergence of miRNAs as drug targets, controls were difficult to identify (see below), and we sought alternatives as suggested by good practice^33^. On the basis of prior reports^20^,^23^,^25^, we chose miRNA mimics and locked nucleic acid-based miRNA inhibitors (referred to as LNAs) to respectively increase and decrease miRNA activity in a predictable manner. Perturbing individual miRNA inputs with mimics and LNA emulates individual drug–miRNA interactions, while perturbing multiple inputs simultaneously emulates systemic alteration of miRNA-processing pathways. We designed 15 different assay perturbations comprising subsets of mimics and LNAs that span a range of possible off-target and on-target effects (Fig. 1d), and used these perturbations to calculate *Z*′-factors^34^ for assay performance evaluation. Each perturbation is associated with its own *Z*′-factor, providing a comprehensive insight into assay behaviour under different conditions.

### Experimental system

As stated above, for the proof-of-concept we used miR-122 as the target. The assay was tested in HuH-7 cells, a liver cancer cell line used as a model for liver tumour^35^, hepatitis C virus infection^36^ and miR-122 drug discovery^37^. Studies showed that miR-122 has an effect on both liver cancer^30^,^38^ and hepatitis C virus replication^31^, making it a promising drug target^39^.

**Figure 2:**
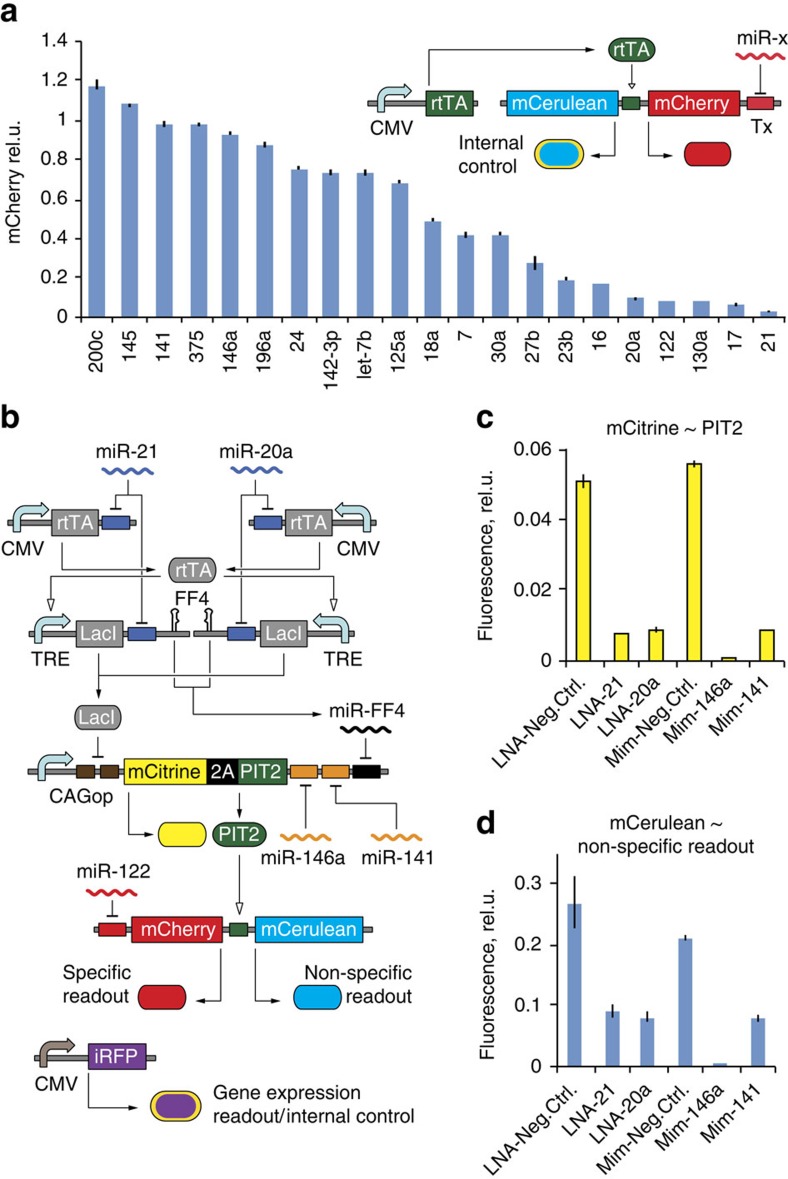
Pilot assay design and characterization. (**a**) Activity profile of 21 endogenous miRNAs in HuH-7 cells. High and low fluorescence indicate, respectively, low and high miRNA activity. Inset: schematics of the bidirectional miRNA reporter assay. (**b**) Schematics of the pilot circuit. miR-21 and miR-20a are the high inputs, miR-146a and miR-141 are the low inputs. mCitrine is a 2A-peptide linked reporter to read the immediate AND gate output. miR-122 is the drug target and iRFP reports on gene expression fluctuations. (**c**,**d**) Expression levels of the immediate AND gate reporter mCitrine (**c**) and non-specific assay readout mCerulean (**d**) following perturbations of individual non-specific inputs (see Fig. 1d, perturbations 2, 3, 11 and 12). Transfections are described in Supplementary Tables 5 and 6. The bar charts show mean±s.d. for biological triplicates. 2A, self-cleaving peptide; iRFP, near-infrared red fluorescent protein; LNA, locked nucleic acid (miRNA inhibitor); Mim, miRNA mimics; PIT2, Streptogramin-responsive transactivator (Pristinamycin-induced protein (Pip) fused to p65); rel.u., relative units.

To determine non-specific assay inputs, we looked for miRNAs expressed at either high or low levels as required by our approach (Fig. 1c). We measured the activities of 21 miRNAs^40^ with bidirectional reporters (Fig. 2a), and retained miR-145, -141, -375 and -146a as non-specific low inputs; and miR-21 and -20a as high inputs. Initially we planned to validate the assay with both RNA-based and chemical or genetic agents known to affect miR-122 or the RNAi pathway. We identified a number of relevant small molecules^25^ and genetic modulators^41^,^42^ and tested them as potential positive controls using bidirectional miRNA reporters. In brief, when using our reporter systems these modulators were insufficiently active and/or specific to serve as positive validation controls (Supplementary Fig. 1; Supplementary Note 1). Next, we tested miRNA mimics and LNAs^20^,^25^ with satisfactory outcomes. We chose miR-146a and -141 as low, and miR-21 and -20a as high inputs for the non-specific RNAi module after confirming their mutual orthogonality (Supplementary Fig. 2a–d).

### Assembly and testing of pilot assay

We optimized individual circuit building blocks for function and dynamic range under a subset of validation perturbations. We evaluated synthetic transactivators for the central knot^32^ and chose the streptogramin-responsive transactivator (Pristinamycin-induced protein (Pip) fused to p65; PIT2)^43^. Next, we calibrated DNA composition of the high input sensor to maximize the effect when high inputs are inhibited, and assessed the position effect of low input miRNA-binding sites in the knot's 3′-UTR to maximize the effect when low inputs are activated (Supplementary Note 2; Supplementary Fig. 2e–i). The optimized components were assembled in a circuit dubbed ‘pilot assay' (Fig. 2b). The layout follows the structure in Fig. 1c, with the addition of an auxiliary fluorescent reporter mCitrine coupled to the PIT2 transactivator via 2A peptide linker for characterization purposes. The circuit senses five different miRNA inputs, of which four feed into the non-specific RNAi module. Initially we tested a subset of perturbations by targeting each non-specific input individually. We found that the assay performance was satisfactory when judged by the changes in mCitrine/PIT2 (Fig. 2c), but it deteriorated at the non-specific readout level for both high and low input modulation, leading to *Z*′-factors <0.5, reflecting merely ‘acceptable' assay performance (Fig. 2d). We concluded that the pilot assay was not robust enough and considered alternative topologies.

### Alternative assay design and simulations

**Figure 3:**
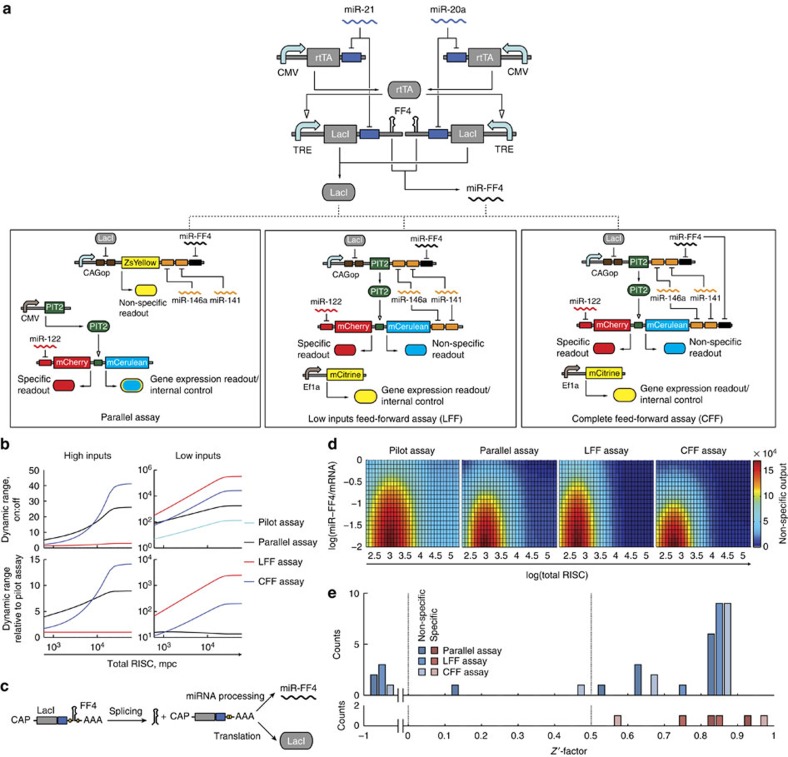
Design and validation of alternative assay layouts. (**a**) Schematic representation of different assay topologies. The sensor genes for two high inputs are common to all designs. The components specific to individual layouts are shown in separate panels. mCherry serves as the specific module readout in all layouts. ZsYellow is the non-specific RNAi module readout in the parallel assay, and mCerulean in the LFF and CFF assays. The gene expression readout in parallel assay is mCerulean. In LFF and CFF assays it is mCitrine. (**b**) A sample of simulation results showing how different assays respond to changes in parameter values, here the total cytoplasmic concentration of RNA-induced silencing complex (RISC) in molecules per cell (mpc) units. See Supplementary Note 3 for details on simulating the dynamic range for high and low inputs. (**c**) The construct expressing LacI and miR-FF4 from the same mRNA. Ratio of mature miR-FF4 to LacI-mRNA can be used to detect influence of candidate molecules on miRNA-processing machinery. (**d**) Simulated dependency of the non-specific RNAi module readout on changes in total RISC concentration and processing efficiency of miR-FF4, as reflected in miR-FF4/LacI-mRNA ratio. (**e**) Experimentally measured *Z′*-factor histograms for the three different assays tested with the 15 validation perturbations (Fig. 1d). Blue bars represent the *Z′*-factors of perturbations probing the non-specific module, and red bars represent perturbations specifically modulating miR-122. Transfections are described in Supplementary Table 7. All data points are mean values of biological triplicates. Ef1a, promoter of elongation factor 1-alpha 1 protein.

The first alternative is the ‘Parallel assay' where specific and non-specific modules are not connected by the PIT2 ‘knot' transactivator. Non-specific effects are reflected in a fluorescent protein ZsYellow replacing the PIT2 knot. The specific module is a PIT2-induced bidirectional reporter of miR-122 activity, driving mCherry furnished with miR-122-binding sites and a reference mCerulean. The latter also serves as a global gene expression readout and transfection control, similar to iRFP in the pilot assay. The two other architectures extend the pilot assay circuit with additional feed-forward loops^44^ that proved their utility in high input sensing^8^. We implemented the motifs by augmenting the non-specific readout mRNA with binding sites for miRNAs that bind to the PIT2 knot. In an implementation called ‘low inputs feed-forward (LFF) assay', only the low inputs miR-146a and -141 target the readout; in the ‘complete feed-forward (CFF) assay', these are miR-146a, -141 and -FF4 (Fig. 3a).

We explored these designs *in silico* using mechanistic models of the four architectures (Supplementary Note 3; Supplementary Fig. 3). We calculated the dynamic range of the non-specific readout by alternating *in silico* between high and low non-specific miRNA input concentrations. For high inputs, we concluded that parallel and CFF architectures are superior, and that under wide range of parameter values, the CFF assay improves 2–3-fold over the parallel assay (Fig. 3b; Supplementary Figs 4–6). For low inputs, LFF comes at the top and CFF is close second best. To simulate sensitivity of assays to global changes in the RNAi pathway, we mapped non-specific readout expression as a function of RNA-induced silencing complex concentration and miR-FF4/LacI-mRNA ratio, the latter being a proxy for miRNA-processing efficiency (Fig. 3c,d). Parallel and CFF assays are most sensitive to changes in these parameters. Because the miR-FF4-binding site is embedded in the readout mRNA's 3′-UTR, CFF is slightly more sensitive than the parallel assay. Thus simulations suggest CFF as the optimal architecture.

### Validation of alternative assays

We quantitatively validated and characterized all three variants using a complete set of input perturbations (Fig. 1d), due to uncertainties in simulating complex networks. *Z*′-factors for specific targeting of miR-122 are >0.5 (excellent) for all assays (Fig. 3e, lower panel). *Z*′-factors corresponding to all perturbations involving non-specific inputs form distinct distributions in each of the three assays (Fig. 3e, upper panel). As predicted by the model, the CFF assay has the smallest number of *Z*′-factors <0.5 (2 out of 13), with the rest being ‘excellent'. With other assays, the lack of miR-FF4 feed-forward loop leads to lower performance when high inputs are perturbed. By applying the ‘best-worst-case' as an indicator of the system's weakest link^45^, we judge the CFF assay to be superior.

### In-depth validation of CFF assay

**Figure 4:**
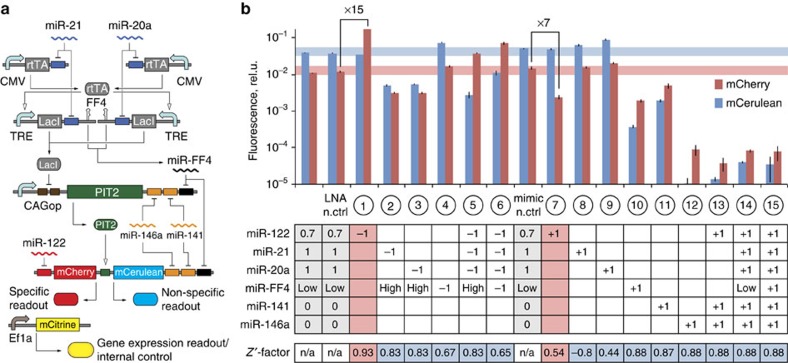
In-depth validation of the CFF assay. (**a**) Schematic representation of the CFF assay circuit used in these experiments, reproduced here from Fig. 3 for convenience. (**b**) Performance of the CFF assay when tested with the 15 validation perturbations. The table describes the miRNA/LNA mixtures. Each column shows, which miRNA(s) are changed relative to HuH-7 background (grey columns). ‘−1' indicates inhibition of a miRNA using LNAs and ‘+1' indicates induction of a miRNA with mimic(s). Grey columns show miRNA expression pattern (0—not expressed, 1—highly expressed, 0.7—intermediate level of the drug target miR-122) in the HuH-7 ground state. Pink columns emphasize specific modulation of miR-122. Pale blue and pink bands indicate the range in which the changes in the non-specific and the specific module readouts, respectively, are insignificant. *Z′*-factors for the different perturbations are displayed in the bottom row: values >0.5 indicate ‘excellent' performance, >0 are ‘acceptable', while the ones <0 are not suitable for screening. Pink and blue shades highlight *Z′*-factors calculated for specific and non-specific module readouts, respectively. Transfections are described in Supplementary Table 8. All bars are mean±s.d. of biological triplicates.

The detailed data of CFF assay (Fig. 4a) response to all perturbations are displayed in Fig. 4 and Supplementary Fig. 7. Zooming into Fig. 4b, we see that specific changes in miR-122 are readily discovered. For the non-specific RNAi module, the induction of miR-20a and miR-21 and the inhibition of miR-FF4 generate the smallest effects, while under other perturbations the assay performs very well. Weak response to increasing high inputs is expected, as they already exert most of their effect in the default state and their main purpose is to serve as detectors of non-specific miRNA inhibition.

**Figure 5:**
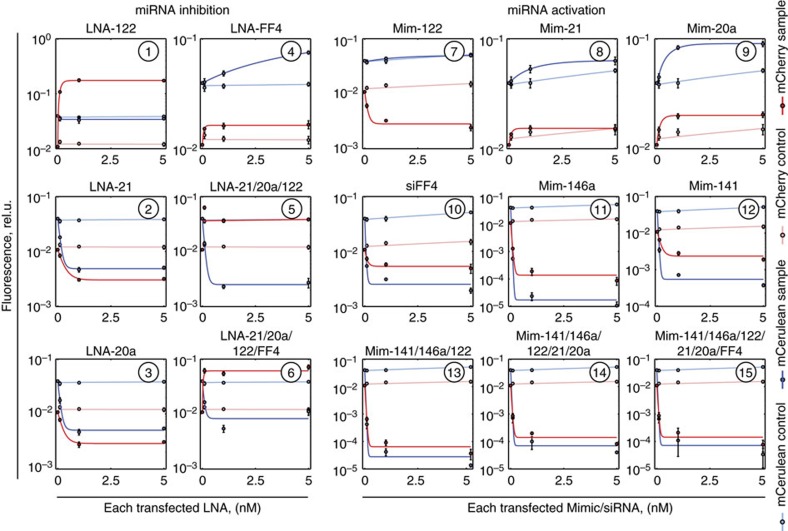
Dose response of assay readouts to gradual change in perturbation strength. Pale blue and red lines show dose–response behaviour with the relevant controls. The dose–response curves were fitted with exponential functions to serve as a visual guide. *Z′*-factors for all data points are reported in Supplementary Table 1. Transfections are described in Supplementary Tables 8 and 9. All data points are mean±s.d. of biological triplicates.

We quantified assay's sensitivity to intermediate perturbation strength by measuring dose response across 50-fold intensity variation (Fig. 5; Supplementary Table 1). The specific module is sensitive to all perturbations in this range; only miR-122 upregulation at the lowest concentration of mimic-122 shows a decreased *Z*′-factor (*Z*′=0.43). The non-specific RNAi module exhibits shallow dose response to increase in high inputs and decrease in miR-FF4, consistent with the end-point data. On the contrary, assay sensitivity to low input activation with respective mimics is very high, resulting in strong readout reduction already at the lowest tested concentration. The asymmetry in response to the two types of inputs is advantageous, since it precludes balancing of two opposing unspecific effects of the same strength. This is confirmed on simultaneous activation of all non-specific inputs with respective mimics, whereby the effect of perturbing low inputs overrides that of high inputs. Last, in all cases where the intended drug target miR-122 is affected simultaneously with other miRNAs, we observe changes in non-specific RNAi module readout. These perturbations emulate a scenario when the intended target is modulated together with a number of off-targets. For all such cases the assay correctly reports the side effects. For further details, see section ‘Assay comparison to bidirectional reporters' below.

### Automated screening of small molecule library

**Figure 6:**
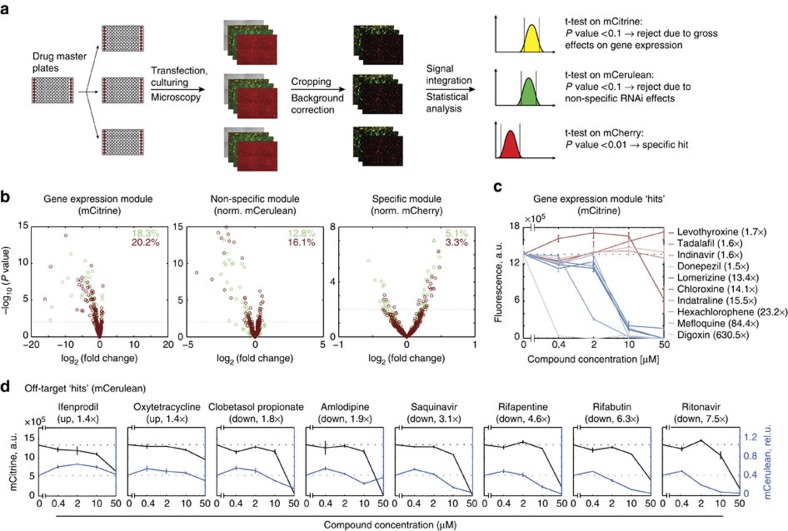
Screening of NIH clinical collection 1 and 2. (**a**) Schematic representation of the small-molecule screen and the data-processing pipeline. (**b**) Volcano plots of screening results with each point representing the mean of a biological triplicate. *X* values are fold changes of the candidate triplicate compared with the plate average, and *y* values represent the *P* value of a two-sided *t*-test of each triplicate compared with the plate averages. Green dots are from screen 1, red ones from screen 2. Horizontal dotted lines are drawn at *P*=0.1 for the gene expression module (mCitrine) and the non-specific RNAi module (normalized mCerulean), and at *P*=0.01 for the specific module (normalized mCherry). (**c**) Dose response of assay gene expression module readout mCitrine to select compounds originally excluded based on this readout in the automated screen. Compounds found to downregulate gene expression readout mCitrine in the automated screen are in shades of blue, and those upregulating mCitrine are in shades of red. (**d**) Dose response of gene expression and non-specific readouts for select compounds excluded based on the non-specific RNAi module readout in the automated screen. Black lines show gene expression module readout (mCitrine); blue ones show the non-specific module readout (normalized mCerulean). Values in **c** and **d** are mean±s.d. of biological duplicates. Transfections are described in Supplementary Tables 10–12. a.u., absolute units.

Following extensive assay optimization and validation, we performed a pilot small molecule screen. First, we adapted the assay to a high-throughput screening protocol^33^ by automating compound dilution and transfection, and developing an image-processing pipeline for 96-well plates (Fig. 6a, Methods section). The assay was revalidated with the automated protocol using select perturbations and was found to retain its performance (*Z*′>0.5). As the pilot library, we chose the NIH Clinical Collections 1 and 2 with a total of 726 compounds. We tested the library twice using automated liquid handling, transient transfections and triplicate measurements. Assuming that most compounds are inactive^34^, we used all readouts from an entire 96-well compound plate as the reference distribution that is compared, using two-sided *t*-test, with triplicate measurements of individual compounds from the same plate (Supplementary Figs 8 and 9). A *P* value cutoff of *P*<0.1 (that is, a compound has a ‘true' effect on a given readout with at least 90% probability) was used to exclude a compound from further analysis based on changes in gene expression readout mCitrine. In screens 1 and 2, 18.3% and 20.2% of compounds, respectively, were excluded. The same criterion was applied to non-specific RNAi module readout (normalized mCerulean), respectively excluding 12.8% and 16.1% of compounds as potential non-specific effectors. For hit identification with the specific module readout (normalized mCherry) we used *P*<0.01 cutoff, identifying 39 and 31 hits, respectively, corresponding to an apparent hit rate of 5.1% and 3.3% (Fig. 6b). However, as the relative magnitude of the effects is <30% for all cases, many hits might be false-positives; therefore, secondary validation is needed.

### Validation of screening hits

We followed up on the miR-122 hits and some of the excluded compounds by measuring dose response of assay readouts. We looked for compounds that significantly affected at least one fluorescent readout in both screen replicas and ranked them according to mean deviation from reference distribution. We measured dose-response of the top 10 modulators of the gene expression module (four up- and six downregulators, Supplementary Fig. 10) and top eight compounds affecting the non-specific module (two up- and six downregulators, Supplementary Fig. 11). We reproduced the effects on the gene expression module with 8 out of 10 compounds, whereas Indinavir and Donepezil (1.6- and 1.5-fold change in the initial screen) failed to elicit the expected response (Fig. 6c; Supplementary Fig. 12). Similarly, compounds that reduced the readout of the non-specific RNAi module in the screen also reduced it in a dose-dependent manner. However, all of them affected the gene expression readout mCitrine at a concentration of 50 μM, five times higher than the one used in the screen, suggesting additional effects on gene expression or cell health. Among compounds that elevated the non-specific module readout, Ifenprodil did so in a dose-dependent manner but Oxytetracyclin could not be confirmed (Fig. 6d). For the seven miR-122 hits, including three up- and four downregulators (Supplementary Fig. 13), we confirmed their lack of effect on gene expression and non-specific module readouts apart from an occasional effect on mCitrine at 50 μM. However, modest modulation of miR-122 activity observed in the screen (<1.3-fold) could not be reproduced. On repeated testing with a miR-122 bidirectional reporter, we confirmed the lack of anti-miR-122 effect (Supplementary Fig. 14), indicating that the hits were likely false-positives resulting from ‘multiplicity of testing' problems^46^.

### Assay customization

**Figure 7:**
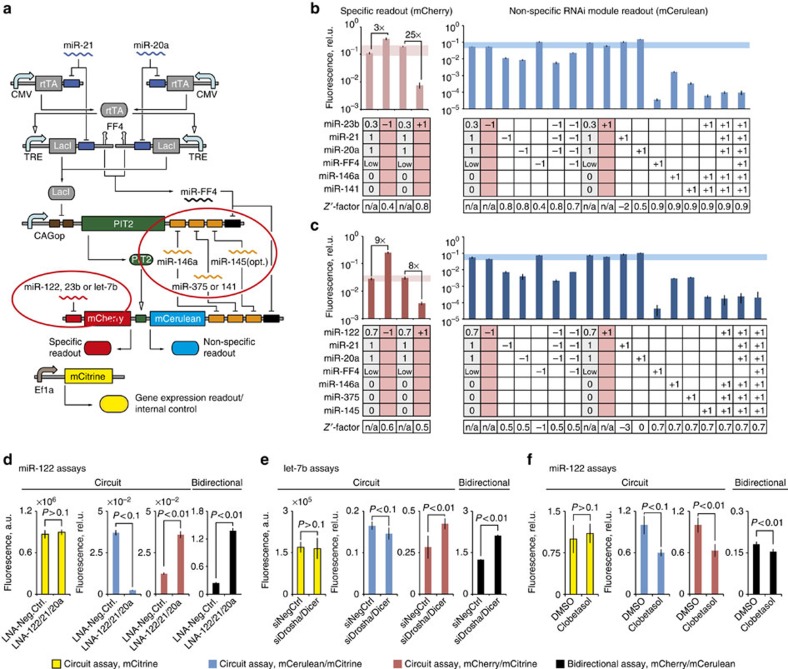
Assay customization and benchmarking. (**a**) Schematic representation of new customized assay circuits with highlighted modifications (red ellipses). (**b**,**c**) Validation of customized assays. Response of the miR-23b drug discovery assay (**b**) and miR-122 drug discovery assay with augmented non-specific inputs (**c**) to different perturbations of the inputs with appropriately adjusted validation perturbations (Fig. 1d). Each column shows which miRNA(s) are changed relative to HuH-7 background (grey columns, 0—not expressed, 1—highly expressed, 0.3 or 0.7—intermediate level). ‘−1' indicates inhibition of an miRNA using LNA(s) and ‘+1' indicates induction of an miRNA with mimic(s). Pink columns emphasize specific modulation of miR-23b or miR-122. Faint blue and red bands indicate the range in which the changes in the non-specific and specific module readouts, respectively, are insignificant. *Z*′-factors for the different perturbations are displayed in the bottom row: values >0.5 indicate ‘excellent' performance, >0 are ‘acceptable', while the ones <0 are not suitable for screening. (**d**–**f**) The effects of non-specific modulators on assay circuit readouts (yellow, blue and red bars) as compared with bidirectional reporter assay readout (black bars, see schematics in Fig. 2a). The respective miRNA drug targets, types of assays and the readouts are indicated in the panels. Data in (**f**) for the circuit assay are pooled from the two triplicate runs of the small molecule screens (Fig. 6) and normalized to the measured plate means. Transfections are described in Supplementary Tables 13–17. Bars in **b** and **c** are mean±s.d. of biological triplicates, in **d**–**f** of six biological replicates.

The assay can be customized, or reprogrammed, to address different miRNA drug targets and different sets of non-specific inputs. To show this experimentally, we modified specific and non-specific modules, generating two new assay circuits. The first is a miR-23b drug discovery assay circuit obtained by replacing miR-122-binding sites with those for miR-23b. The second circuit has an augmented non-specific input set obtained through replacement of miR-141-binding sites with the sites for miR-375 and miR-145 (Fig. 7a). We validated the new assay with perturbations modified to match the new miRNA drug target and non-specific inputs. In the miR-23b assay, the *Z*′-factors for individual perturbations were comparable to those measured with the miR-122 assay, with the exception of miR-23b inhibition that resulted in ‘acceptable', but not ‘excellent', performance (*Z*′=0.38, Fig. 7b). This can be explained by the relatively low endogenous activity of miR-23b in HuH-7, and may be rectified with a more sensitive sensor for miR-23b activity. For the miR-122 assay with augmented non-specific inputs, most of the *Z*′-factors were comparable to the original miR-122 assay, apart from lower sensitivity to LNA-FF4 due to weaker miR-FF4 effect in this circuit (Fig. 7c). These data show that specific and non-specific inputs can be swapped and augmented in a modular fashion requiring only minor optimizations, resulting in drug discovery assays for new on- and off-targets.

### Assay comparison to bidirectional reporters

A key assay feature is the ‘filtering' of candidate compounds that would have been otherwise considered specific in simple luciferase or fluorescent reporter assays. To illuminate this advantage, we sought compounds or perturbations that affect RNAi pathway non-specifically. The simplest example is a validating perturbation that affects miR-122 together with the non-specific inputs, for example, a mixture of multiple LNAs with LNA-122 (Fig. 1d, Perturbation 5). When this combination is applied to a bidirectional reporter (Fig. 7d, right chart), it can be mistaken for a specific modulator; analysis of the circuit assay readouts suggests otherwise, because the non-specific RNAi readout mCerulean is significantly reduced (Fig. 7d, charts on the left). Next, we noticed that in bidirectional reporter assays, siRNAs^47^ against the RNAi pathway proteins Drosha and Dicer affected the activity of miR-20a and let-7b, but not of miR-122 (Supplementary Fig. 1j). To illustrate the distinction between specific and non-specific let-7b targeting, we exchanged miR-122-binding sites in the original CFF circuit with those for let-7b, resulting in an assay for let-7b. We subjected this new assay, as well as a bidirectional let-7b reporter, to siDrosha/siDicer mixture. The bidirectional reporter shows clear effect on let-7b. In the circuit assay, the gene expression readout mCitrine was unchanged, while the non-specific module readout mCerulean changed relative to the control with *P*<0.1. In our decision tree, this compound is flagged as a non-specific hit (Fig. 7e). Last, we looked at some of the compounds that affected the non-specific RNAi module in the automated screen. Among those, Clobetasol propionate also had an effect on miR-122 in the bidirectional reporter assay. The measurements with Clobetasol propionate done with circuit-based miR-122 assay and with a bidirectional reporter show that this compound would have been identified as specific modulator in a bidirectional assay, but it is excluded in the complete multi-module circuit assay (Fig. 7f). These data show that non-specific compounds affecting RNAi can be identified with our assay, while they would be considered specific with simple bidirectional reporter assays.

## Discussion

In this report we describe a large-scale mammalian gene circuit serving as an assay for drug discovery against miRNA targets, enabling highly precise identification of specific target modulators with high throughput. Until now off-target effects have been usually assessed in secondary screens^48^. Although gene circuits have been suggested for use in small-molecule screening before^9^,^11^, this is to the best of our knowledge, the first multi-input, customizable assay. It might as well be one of the first large-scale mammalian synthetic circuits that can be directly applied to an unmet technological need. Transient transfection of plasmid sets is sufficient to establish the assay because the readouts are averaged across transfected wells. Thanks to a cell-level computation^5^ the circuit performs over a set of potential off-target inputs, assay readouts carry rich information that is difficult to measure otherwise. We used miRNA as a drug target in this study because off-target effects are expected due to shared maturation pathway and common mechanisms of action. To read out the effect of a compound on a certain miRNA, we employ synthetic, fully complementary binding sites to record the miRNA's activity. They are used as robust and representative reporters of the activity that a miRNA is expected to have on its many endogenous targets.

The same general strategy can be applied in just about any cell-based screening scenario given that appropriate sensing–computing networks are designed and experimentally implemented. For example, very recent report uncovered four consensus genes related to toxicity^49^. Promoters driving these genes can be integrated with an assay such as ours and add to the information content of the non-specific reporter. Our results validate the basic premise behind rationally designed biological information-processing networks, namely that appropriate design frameworks^3^, inspired by and built on solid engineering principles, can give rise to multiple systems with diverse properties and intended uses, eliminating the need to construct each new system from scratch. This is not to say that these design frameworks have reached the ‘plug and play' stage. In this study we describe four different circuits that were tested extensively *in silico* and in experiments. Eventually, we arrived at a well performing, customizable architecture and implemented an automated screening protocol, suggesting that these circuits can be used ‘as is' in exploratory screening campaigns. Our engineering efforts have also augmented the toolkit of synthetic biology with new concepts such as the nested feed-forward motif from CFF assay. Thus, encounters of abstract concepts with real-life applications not only address specific needs, but also provide rich data that are applicable in other contexts of circuit engineering.

## Methods

### Plasmid construction

Standard cloning techniques were used to construct plasmids. *Escherichia coli* DH5α served as the cloning strain, cultured in LB Broth Miller Difco (BD) supplemented with appropriate antibiotics (Ampicillin, 100 μg ml^−1^, Chloramphenicol, 25 μg ml^−1^ and Kanamycin, 50 μg ml^−1^). Enzymes were purchased from New England Biolabs (NEB). Phusion High-Fidelity DNA Polymerase (NEB) was used for PCR amplification. Oligonucleotides used as primers or for annealing were purchased from Microsynth, IDT or Sigma-Aldrich. Digestion products or PCR fragments were purified using GenElute Gel Extraction Kit or Gen Elute PCR Clean Up Kit (both Sigma-Aldrich). Ligations were performed using T4 DNA Ligase (NEB) at 16 °C for 1 h for sticky end overhangs or at 4 °C overnight for blunt-end ligation, followed by transformation into chemically competent cells and plating on LB Agar plates with appropriate antibiotics. Clones were screened by colony-PCR using Quick-Load *Taq* 2X Master Mix (NEB) or by test restriction. Plasmids were sequenced by Microsynth. Detailed cloning procedure for each plasmid can be found in Supplementary Table 2, with primers listed in Supplementary Table 3 and gBlocks in Supplementary Table 4.

### Cell culture and transfection

HuH-7 cells were received from the Health Science Research Resources Bank of the Japan Health Sciences Foundation (catalogue no. JCRB0403, Lot # 07152011) and cultured at 37 °C, 5% CO_2_ in DMEM, low glucose, GlutaMAX (Life Technologies, catalogue no. 21885-025), supplemented with 10% FBS (Sigma-Aldrich, catalogue no. F9665 or Life Technologies, catalogue no. 10270106) and 1% Penicillin/Streptomycin Solution (Sigma-Aldrich, catalogue no. P4333). Cells were passaged every 3–4 days using 0.25% Trypsin-EDTA (Life Technologies, catalogue no. 25200-072). Transfection was performed using Lipofectamine 2000 Transfection Reagent (Life Technologies, catalogue no. 11668-019) in uncoated 24-well plates (Thermo Scientific, catalogue no. 142475), 96-well plates (Thermo Scientific, catalogue no. 167008) or black μ-clear 96-well plates (Greiner Bio-One, catalogue no. 655090). For transfections in 24-well plates HuH-7 cells were seeded 1 day before transfection at a density of 65,000 cells per well in 500 μl complete medium. The medium was replaced before transfection with medium supplemented with Doxycycline hyclate (Fluka, catalogue no. 44577) at a final concentration of 1 μg ml^−1^. For 96-well plates, transfection was performed in suspension. Specifically, HuH-7 cells were seeded at a density of 30,000 cells per well in 100 μl in complete medium supplemented with Doxycycline hyclate (Fluka, catalogue no. 44577) at a final concentration of 1 μg ml^−1^ right before transfection (suspension transfection protocol from the manufacturer). Transfections were performed at 80–90% cell confluence. Plasmids were purified from 100–400 ml cultures of *Escherichia coli* DH5α grown overnight at 37 °C at 200 r.p.m. in LB Broth Miller Difco (BD) supplemented with appropriate antibiotic using HiPure Plasmid Filter Maxi/Midi Kit (Invitrogen) or PureYield Plasmid Midiprep Kit (Promega). After plasmid purification, an additional purification step was performed using Endotoxin Removal Kit (Norgen Biotek Corporation). DNA amounts were quantified using Nanodrop (ND-2000) and integrity was verified by agarose gel electrophoresis. The purified plasmids were mixed according to Supplementary Tables 5–39 and diluted with 50/25 μl Opti-MEM I Reduced Serum (Gibco, Life Technologies catalogue no. 31985-962) per sample for 24/96-well plates, respectively. If needed, microRNA mimics, siRNAs and LNA-inhibitors were added to the plasmid mix. Mimics were purchased from Thermo Scientific, siRNAs from Microsynth and LNA inhibitors form Exiqon (Supplementary Table 40). Lipofectamine 2000 was used at a Lipofectamine (μl):DNA (μg) ratio of 2.5:1 and was mixed with 50/25 μl Opti-MEM for 24/96-well plates, respectively. After 5 min incubation at room temperature, the diluted Lipofectamine was mixed with the diluted DNA sample. The mixture was incubated for 20 min at room temperature and added to the cells.

### Compound sources

NSC308847 was received from Santa Cruz Biotechnology, Santa Cruz, USA (catalogue no. sc-207283) and from Fisher Bioservices, Germantown, USA (catalogue no. NSC308847) via the NCI Chemotherapeutic Agents Repository. NSC158959 was received from ASINEX Ltd, Moscow, Russia (catalogue no. BAS 00204187) and from Fisher Bioservices, Germantown, USA (catalogue no. NSC158959) via the NCI Chemotherapeutic Agents Repository. NSC5476 was received via the National Cancer Institute Developmental Therapeutics Program, Bethesda, USA (catalogue no. NSC5476). Enoxacin was received from Sigma-Aldrich Chemie GmbH, Buchs, Switzerland (catalogue no. E3764-500MG). Acriflavine hydrochloride was received from AppliChem via its Swiss reseller Axon Lab AG, Baden-Dättwil, Switzerland (A2952.0010) and Poly-L-lysine hydrobromide from Sigma-Aldrich Chemie GmbH, Buchs, Switzerland (catalogue no. P6516-100MG).

### Small-molecule screening

Small molecules were received from the NIH Clinical Collection program via Evotec. 727 compounds were shipped in 96-well plates, 10 mM in 50 μl dimethylsulphoxide (DMSO; Supplementary Table 41). Purity was guaranteed by supplier. Dilutions were first performed in DMSO to 1 mM and afterwards in complete medium to 30 μM. 50 μl of this compound-containing medium was added to the screening plates. All steps were performed on a Hamilton Microlab STAR Line robot with custom configuration and protocol. The circuit transfection was performed manually in bulk suspension as described above. In large screens with about 40 plates in total, two separate transfected cell batches were prepared for the first and the last 20 plates, respectively, to avoid long waiting times. A batch of 150 ml transfected cells in growth medium was placed in a V-shaped container. Immediately afterwards, 100 μl aliquots containing suspended transfected cells were dispensed into compound-containing wells (96-well plates) by a custom robot program, resulting in a final concentration of 10 μM compound and 1% DMSO. Cells were maintained in uniform suspension by periodic pipetting of the mixture in the large container; shaking the container on the other hand resulted in cell death. Positive and negative controls were placed in rows 1 and 12, containing untransfected cells, pure DMSO, 5 nM LNA-122, 5 nM Mim-122, 5 nM LNA-21 and 5 nM Mim-146a. Cells were assayed after 48 h using microscopy as described below (Supplementary Fig. 15). Data processing is described below.

### Fluorescent microscopy

Cells were measured 48 h after transfection by an inverted Fluorescent Microscope (Nikon Eclipse Ti) using a Fiber Illuminator (Nikon Intensilight C-HGFI), with optical filter sets (Semrock) and a Digital Camera System (Hammamatsu, ORCA R2). The filters are a combination of excitation and emission band pass filters combined with a dichroic filter for each individual fluorescent protein. We measured mCerulean, mCitrine, mCherry and iRFP with the filter set CFP HC (HC 438/24, HC 483/32, BS 458), YFP HC (HC 500/24, HC 542/27, BS 520), TxRed HC (HC 624/40, HC 562/40, BS 593), Cy5.5-A (HC 655/40, HC 716/40, BS 685), respectively. For the high-throughput screening experiments, a Plan Apo λ × 2 objective was used and 2 × 1 frames were acquired to cover the majority of a well. For all other experiments a Plan Fluor × 10 Ph1 DLL objective was used and 2 × 2 frames were acquired.

### Flow cytometry

Samples were analysed 48 h after transfection using a BD LSR Fortessa cell analyzer. Medium was removed and cells were incubated with 150/50 μl phenol-red free Trypsin (0.5% Trypsin-EDTA (Gibco, Life Technologies, catalogue #15400-054) 1:2 diluted with PBS (Life Technologies catalogue no. 10010-56) for 5/15 min for 24/96-well plates, respectively. Detached cells were transferred to small FACS tubes (Life Systems Design, catalogue no. 02-1412-000) and kept on ice. The fluorophores were measured with a combination of excitation lasers and emission filters. For mCherry, we used a 561-nm excitation laser, 600 nm long-pass filter and 610/20 nm emission filter. For mCitrine, we used a 488-nm laser, 505 nm long-pass filter and 542/27 nm emission filter. For mCerulean, we used a 445-nm laser and a 473/10-nm emission filter. For iRFP, we used a 640-nm laser and 780/60 nm emission filter. Photomultiplier tube voltages were checked and adjusted if needed using standard fluorescent beads (SPHERO Rainbow Calibration Particles, RCP-30-5A (eight peaks) and Alignflow Flow Cytometry Alignment Beads, A-16500, Life Technologies) before and after each measurement to ensure constant device performance.

### Luciferase assays

Cells were harvested 48 h post transfection. Supernatant was removed, cells were washed with PBS, and 100 μl 1 × passive lysis solution (Promega) was added (incubation at 37 °C for 15 min). The luciferase reaction was performed using Promega dual luciferase assay kit. We mixed 20 μl of lysed cells with 50 μl of the respective reagent. Measurements were performed with a lag time of 2 and 10 s recoding time using Sirius instrument (Berthold Detection Systems). In the case of sensor saturation, measurements were repeated with half the amount of lysis solution until quantifiable.

### Flow cytometry data and image processing

All flow cytometry experiments were analysed using FlowJo software. Compensation of crosstalk (<1.9%) of mCerulean into the 488–542/27 nm channel of mCitrine was performed if necessary using single-colour controls. The values in the various figures shown as absolute units (a.u.) or relative units (rel.u.) are calculated as follows. (i) Live cells were gated using forward and side scatter. (ii) Within this gate, fluorophore positive gates are constructed using untransfected controls such that 99.9% of cells in this control sample fall outside of the selected gate. (iii) For each positive cell population in a given channel, the mean value of the fluorescent intensity is calculated and multiplied by the frequency of the positive cells to result in absolute intensity (a.u.):


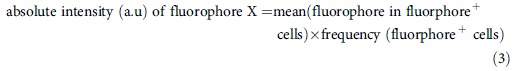


For the relative intensities, the absolute intensity of the fluorophore of interest was divided by the absolute intensity of a constitutively expressed fluorescent protein that was co-transfected with the other plasmids:


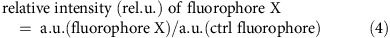


Small-molecule screening data of Fig. 6 was processed as follows: all microscopy images were exported as TIFF files using NIS-Elements Viewer's ‘Export' function. All images were cropped by 460 pixels on both sides, to remove well border artefacts. The background image for each plate in mCerulean and mCitrine channels was calculated by individual averaging of image pixels from non-transfected wells A1 and A12. Then, we subtract (pixel-by-pixel) these averaged background image files from all other images of the same plate. For mCherry, after cropping we first applied MATLAB's msbackadj function (‘Windowsize', 130) row wise for all images, including non-transfected wells A1 and A12, thus performing the first round of background correction. In the second step the background image for each plate was calculated by averaging each pixel of previously corrected mCherry snapshots from non-transfected wells A1 and A12. Finally, we subtracted these averaged background images, pixel-by-pixel, from all other previously corrected mCherry images in the same plate thus performing an additional round of background correction. Next, we create a ‘positive pixel mask' based on mCitrine: each pixel in mCitrine images with an intensity >250 a.u. is defined as positive, all others negative. Finally, we calculate the average for these pixels in all three colours (mCitrine, mCerulean, mCherry).

### Statistical analysis

Ten compound storage plates were used in the screen, with about 80 compounds in each plate. Each plate was ‘replicated' three times for the screening assay, and each compound assayed as a triplicate in three different plates. Triplicate assay plates belonging to separate storage plates were analysed separately. For mCitrine, absolute readouts were compared, while both mCerulean and mCherry readouts were internally normalized to mCitrine values in each well. The set of measurements made with all compounds in a given storage plate (about 240 values in total for each readout) was used as a reference distribution for individual compounds from this plate for the purpose of exclusion and hit identification^34^. Specifically, a triplicate measurement of each compound was compared against its respective reference distribution using a two-sided *t*-test. Compounds that generated non-specific mCitrine and normalized mCerulean readouts that differed from the reference with a *P* value ≤0.1, were considered as potential non-specific modulators and thus were excluded from the analysis. Compounds that generated normalized mCherry readouts that differed from the reference distribution with *P* value ≤0.01, were classified as hits (Fig. 6a). To test whether the data used in the *t*-test are distributed normally, we built histograms of readouts and fitted them to normal distribution using histfit MATLAB function (Supplementary Figs 8 and 9). *Z*′-factors for different perturbations were calculated with the following formula: *Z*′=1–3 × (*σ*(p)+*σ*(n))/|*μ*(p)−*μ*(n)|, where *σ*(p) and *μ*(p) are the s.d. and mean of that perturbation, respectively, and *σ*(n) and *μ*(n) ones of the control. For perturbations including LNAs, the control is measured with scrambled LNAs, and in perturbations involving miRNA mimic, the control is a scrambled miRNA mimic.

### Modelling

Model was built and simulations performed using MATLAB and SimBiology toolbox. All simulations were performed with ‘sundials' solver, with 10^−6^ absolute and 0.001 relative tolerance. The end point of the simulation was chosen at 50 h. Parameter scans were performed with a MATLAB code executing the SimBiology model in a loop using sbiosimulate command with different parameter values. End-point values were used for analysis. Further details can be found in Supplementary Note 3.

## Additional information

**How to cite this article:** Haefliger, B. *et al*. Precision multidimensional assay for high-throughput microRNA drug discovery. *Nat. Commun.* 7:10709 doi: 10.1038/ncomms10709 (2016).

## Supplementary Material

Supplementary Information Supplementary Figures 1-15, Supplementary Tables 1-41, Supplementary Notes 1-3 and Supplementary References
