# Supplementary material for: Precision multidimensional assay for high-throughput microRNA drug discovery
Source: Nat Commun. 2016 Feb 16;7:10709. doi: 10.1038/ncomms10709 (PMC4757758; doi:10.1038/ncomms10709)
Supplement: Supplementary Information — Supplementary Figures 1-15, Supplementary Tables 1-41, Supplementary Notes 1-3 and Supplementary References [file ncomms10709-s1.pdf]

Precision multidimensional assay for high-throughput microRNA drug discovery

Benjamin Haefliger, Laura Prochazka, Bartolomeo Angelici and Yaakov Benenson

Department of Biosystems Science and Engineering, Swiss Federal Institute of Technology (ETH Zürich), Mattenstrasse 26, Basel 4058, Switzerland

To whom correspondence should be addressed: Y.B. (kobi.benenson@bsse.ethz.ch)

Supplementary information

Supplementary Figures

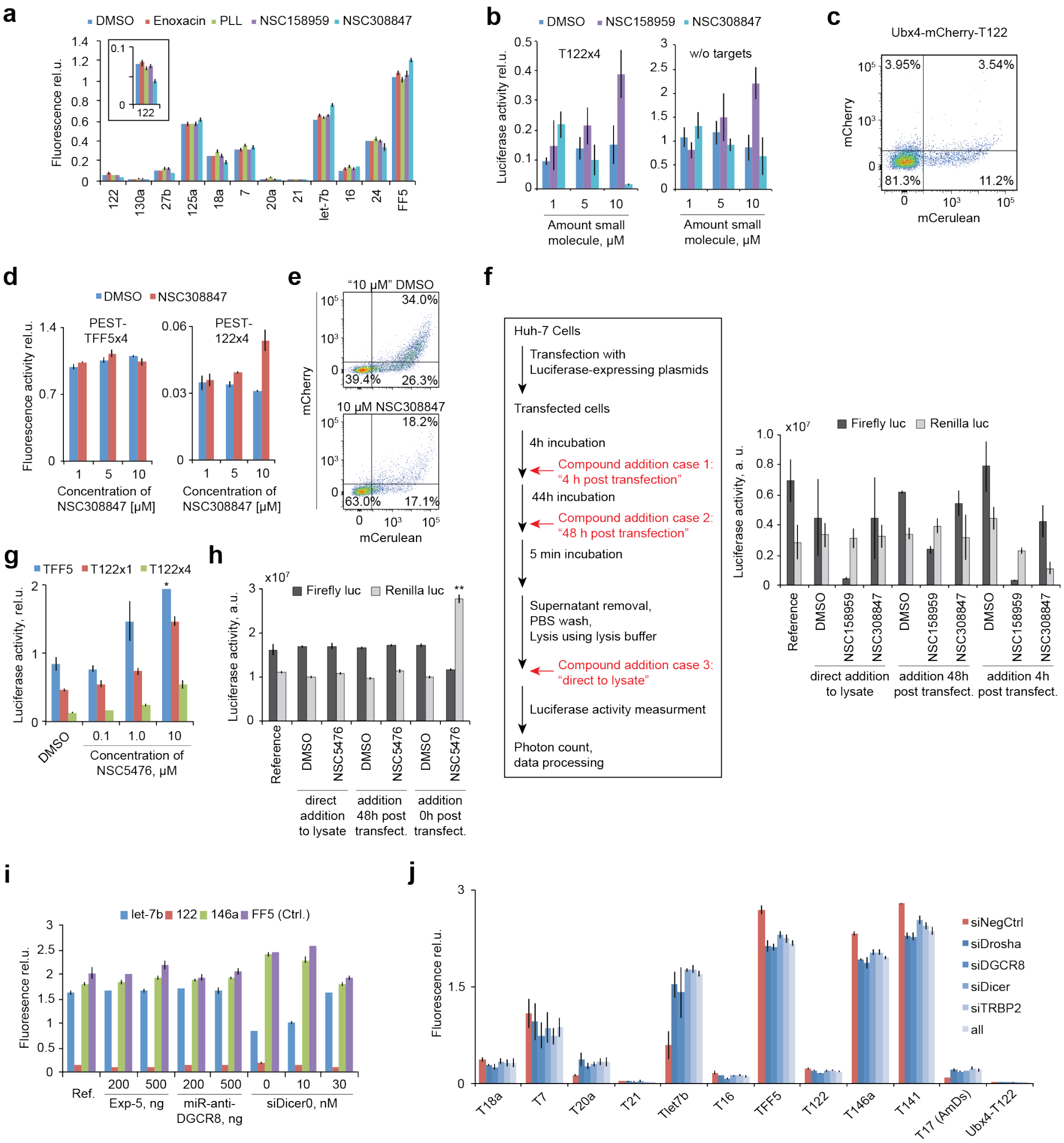

Supplementary Figure 1 Testing of various miRNA modulators. (**a**) Effects of small molecules (10 µM) on different miRNA reporters in HuH-7 cells. Maximal expected expression level is represented by negative control target TFF5. X-axis labels are miRNA names whose fully complementary binding sites are placed in the 3'UTR of the fluorescent reporter (See **Fig. 2a** for reporter schematics) (**b**) Effects of specific miR-122 modulators on luciferase-based reporters. miR-122 reporters (left) are compared with controls (right) to

account for non-specific effects. Different shades of blue represent different small molecule modulators as indicated. (**c**) Flow cytometry scatter plot of bidirectional miRNA activity reporter, expressing wild-type mCerulean as an internal control, and Ubiquitin x4-mCherry-PEST-T122x4 as a destabilized miR-122 reporter. (**d**) The effect of NSC308847 on PEST-tagged mCherry with miR-122 binding sites (left) and scrambled binding sites (right), measured with a bi-directional reporter expressing wild-type mCerulean as an internal control. (**e**) Flow cytometry scatter plot showing the effect of 10 µM NSC308847 on HuH-7 cell transfected with the PEST-destabilized reporter corresponding to data in panel **d** (right) to illustrate effect on expression and cell health. (**f**) Effects of small molecules (10 µM) on *Renilla* or *firefly* luciferase without any miRNA binding sites. The chemicals are added at different time points and/or treatments following the transfection of luciferase-expressing genes into HuH-7 cells, as indicated. Box: Flow diagram of the experimental design to interrogate compounds' effect on luciferase genes without miRNA binding sites. The time points of small molecule addition in different cases are indicated in red. (**g**) Effect of NSC5476 on luciferase bidirectional reporter. * luciferase activity could not be quantified because of oversaturation of the photon counter for all pipettable lysate volumes. (**h**) Effect of NSC5476 (10 µM) on *Renilla* and *Firefly* luciferases. NSC5476 is added at different time point post transfection or directly to cell lysate, as indicated. The procedure is schematically depicted in the flow diagram in Panel **f**. ** calculated luciferase activity from half the lysate due to saturation of the sensor with standard lysate amounts. (**i**) Effects of Exportin-5 overexpression, anti-DGCR8-miRNA and siDicer0 on a subset of candidate reporters (**Fig. 2a**, inset). (**j**) The effects of additional siRNAs[1] on miRNA reporters (**Fig. 2a**, inset). miRNAs selected as pilot circuit inputs were tested together with candidates identified based on deep sequencing data[2]. The reporters were co-transfected with 20 nM siRNA for individual siRNA testing, and with 5 nM of each siRNA for simultaneous siRNA delivery. All bars shown are mean ± s.d. of biological triplicates; scatter plots are representative single measurements of biological triplicates. Transfection setup for this figure is given in **Supplementary Tables 18-27**.

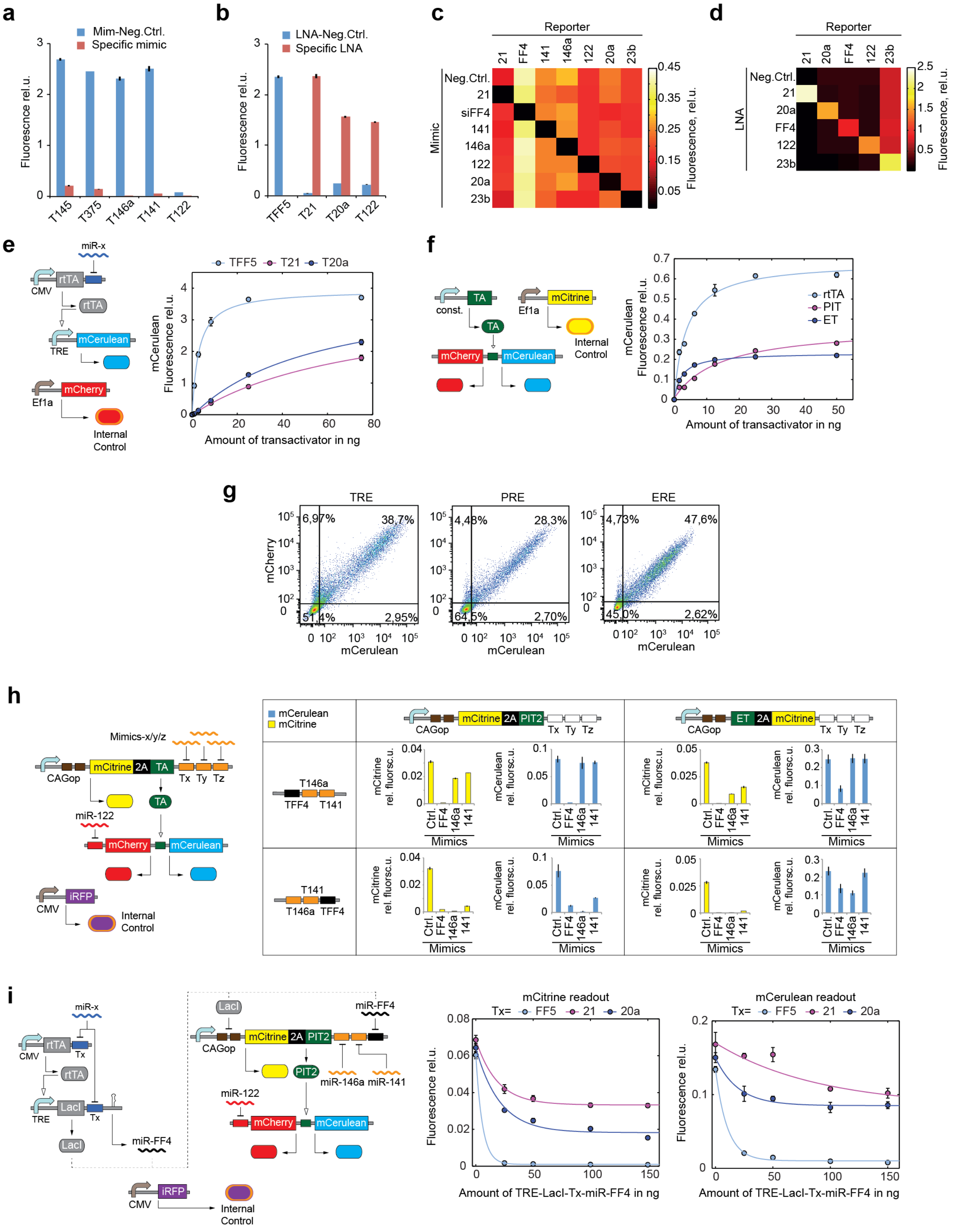

Supplementary Figure 2 Pilot circuit optimization (**a, b**) Testing of miRNA mimics/LNAs (5 nM) with bidirectional reporters (**Fig. 2a**, inset). TFF5 reporter serves as control, representing unrepressed reporter level. miRNA binding sites in the reporters are shown on X axis. (**c**) Orthogonaltiy tests of mimics (5 nM) on bidirectional reporters (**Fig. 2a**, inset) in those cell

lines where the respective miRNA is not expressed for background-free functional characterization. We used HEK293 cells for all mimics except for Mim-20a, which was tested in S2 drosophila cells. Color code of heat map represents the expression of the respective reporter. miRNA mimics and reporters are indicated. (**d**) Orthogonality tests with LNAs (5 nM) on bidirectional reporters (**Fig. 2a**, inset) in HuH-7 cells. (**e**) Dose-response of pTRE-driven fluorescent reporter to varying amounts of rtTA furnished with different miRNA targets, as indicated. FF5 is a scrambled miRNA target and it results in the strongest possible dose-response. (**f**) Dose-response of newly constructed bidirectional reporters to varying amounts of their cognate activators, as indicated in the panel. rtTA dose-response is shown for comparison. (**g**) Flow cytometry scatter plots for the different bidirectional reporters at 25 ng of transactivator as shown in (**f**). (**h**) Comparison of knockdown efficiency of miRNA mimics (5 nM) on two different transactivators with different positioning of the respective targets, as indicated. Yellow bars represent the direct readout of the targeted protein (mCitrine). Blue bars indicate the expression of a fluorescent reporter controlled by this transactivator via a bidirectional promoter. (**i**) Optimization of high input sensor composition in HuH-7 cells. Response of the transactivator output (mCitrine) and the downstream mCerulean output is measured with varying amounts of high input sensor genes, separately for miR-21 and miR-20a sensors. Sensor genes furnished with scrambled FF5 target sequence generate baseline "Off" response. Transfections are described in **Supplementary Tables 28-36**. All data points and bars shown are mean ± s.d. of biological triplicates. TA, transactivator; CMV, cytomegalovirus immediate-early promoter; TRE, TetR responsive element; PRE, PIT2 response element; ERE, ET responsive element; LacI, Lac repressor; rtTA, reverse Tet transactivator; CAGop, CAG promoter followed by an intron with two LacO sites; PIT2, Streptogramin-responsive transactivator (Pristinamycin- induced protein (Pip) fused to p65); ET, ET-dependent transactivator (MphR(A) fused to VP16); iRFP, near-infrared red fluorescent protein; 2A, Self-cleaving peptide.

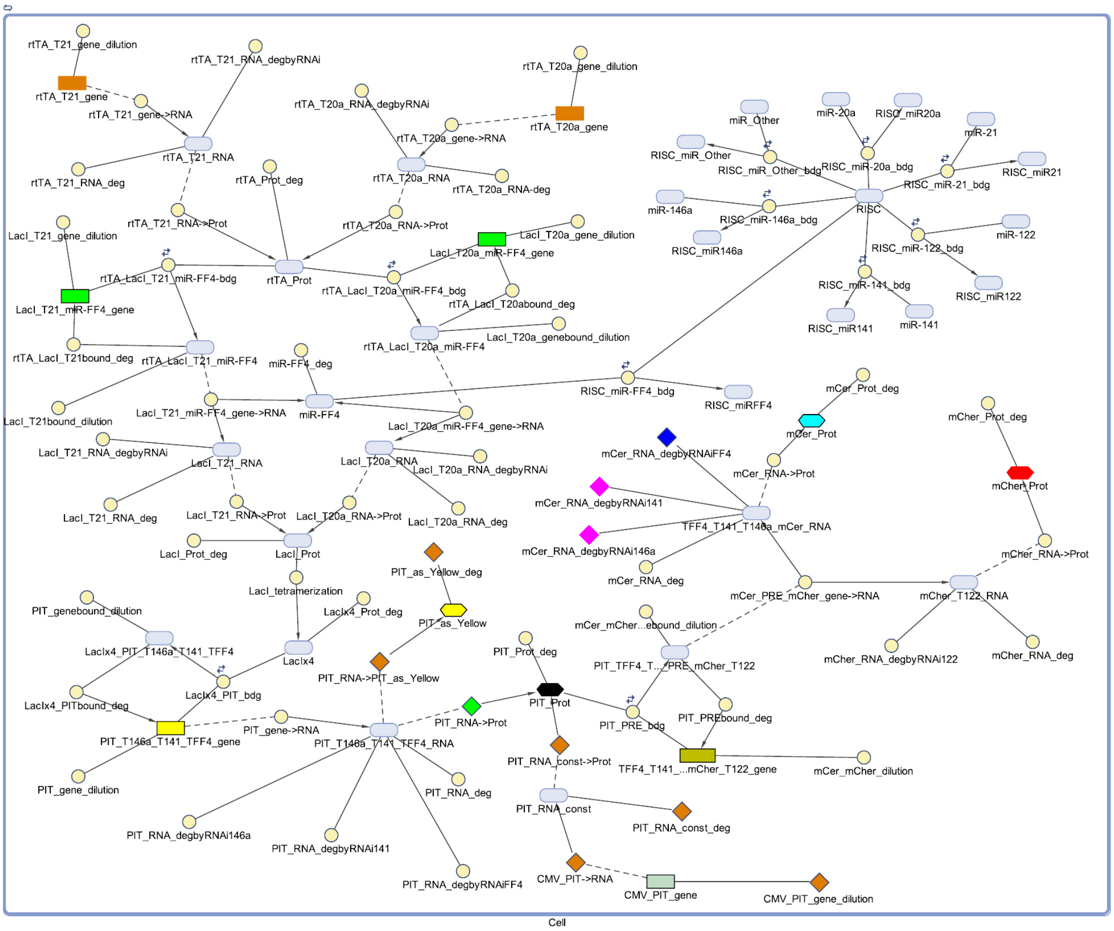

Supplementary Figure 3 SimBiology model used to simulate the different assays. Reactions in green are shared for Pilot, LFF and CFF assays and the ones in purple for LFF and CFF. Orange reactions are specific for the Parallel assay, the blue one for the CFF assay. All other species and reactions are identical for all assays and parameters used for comparison are the same.

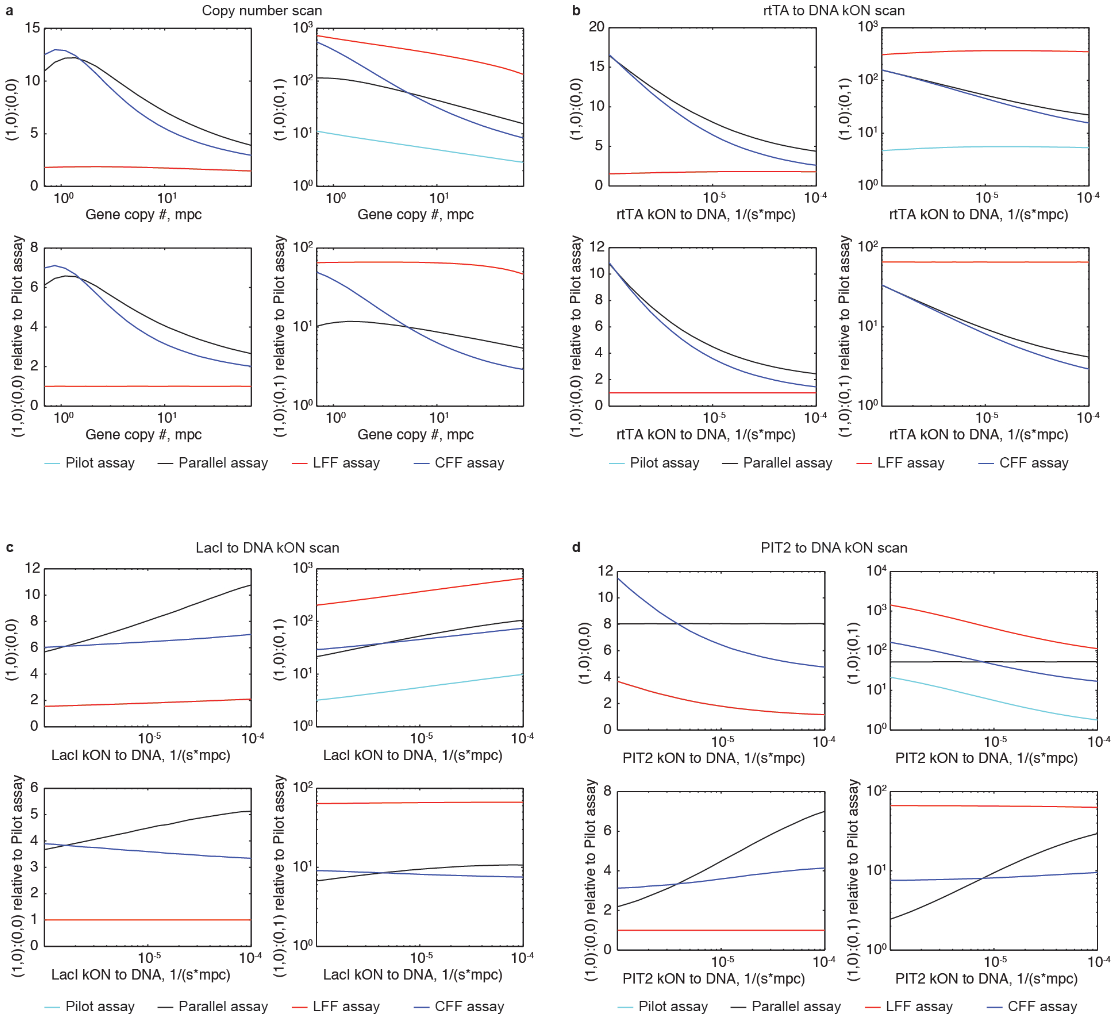

Supplementary Figure 4 Different parameters were scanned with the model shown in **Supplementary Figure 3**. (1,0) represents the On state. In case of (0,0) the high input miRNAs are inhibited or absent, generating the higher of the Off states. (0,1) is usually the lowest of the Off states, where high input miRNAs are inhibited or absent and the low inputs are present. All the plots show relative values of the On-state to either of the two Off-states. (**a**) Copy number scans. (**b**) The association rate constant of rtTA to its promoter is varied. (**c**) The association rate constant of LacI and its operator are varied. (**d**) The association rate constant of PIT2 is varied. mpc, molecules per cell.

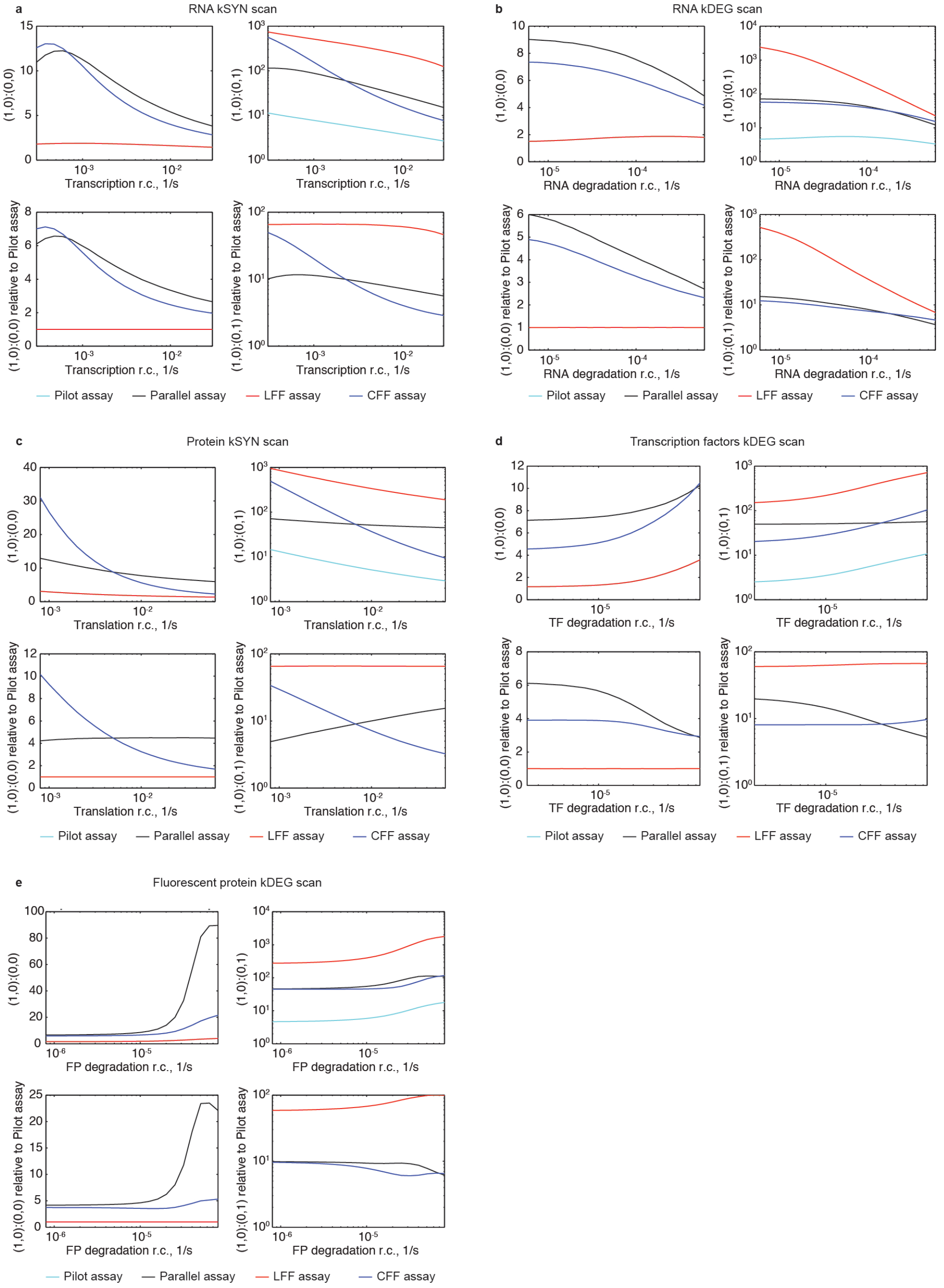

Supplementary Figure 5 RNA and protein synthesis and degradation parameters were scanned using the model shown in **Supplementary Figure 3**. Since the half-life of transcription factors and fluorescent proteins vary significantly we assessed their behavior separately. The explanation of the plot data is in the legend to **Supplementary Figure 4**. The parameters scanned are as follows: (**a**) RNA synthesis rate constant. (**b**) RNA degradation rate constant. (**c**) Protein synthesis rate constant. (**d**) Degradation of transcription factors rate constant. (**e**) Degradation of fluorescent proteins rate constant.

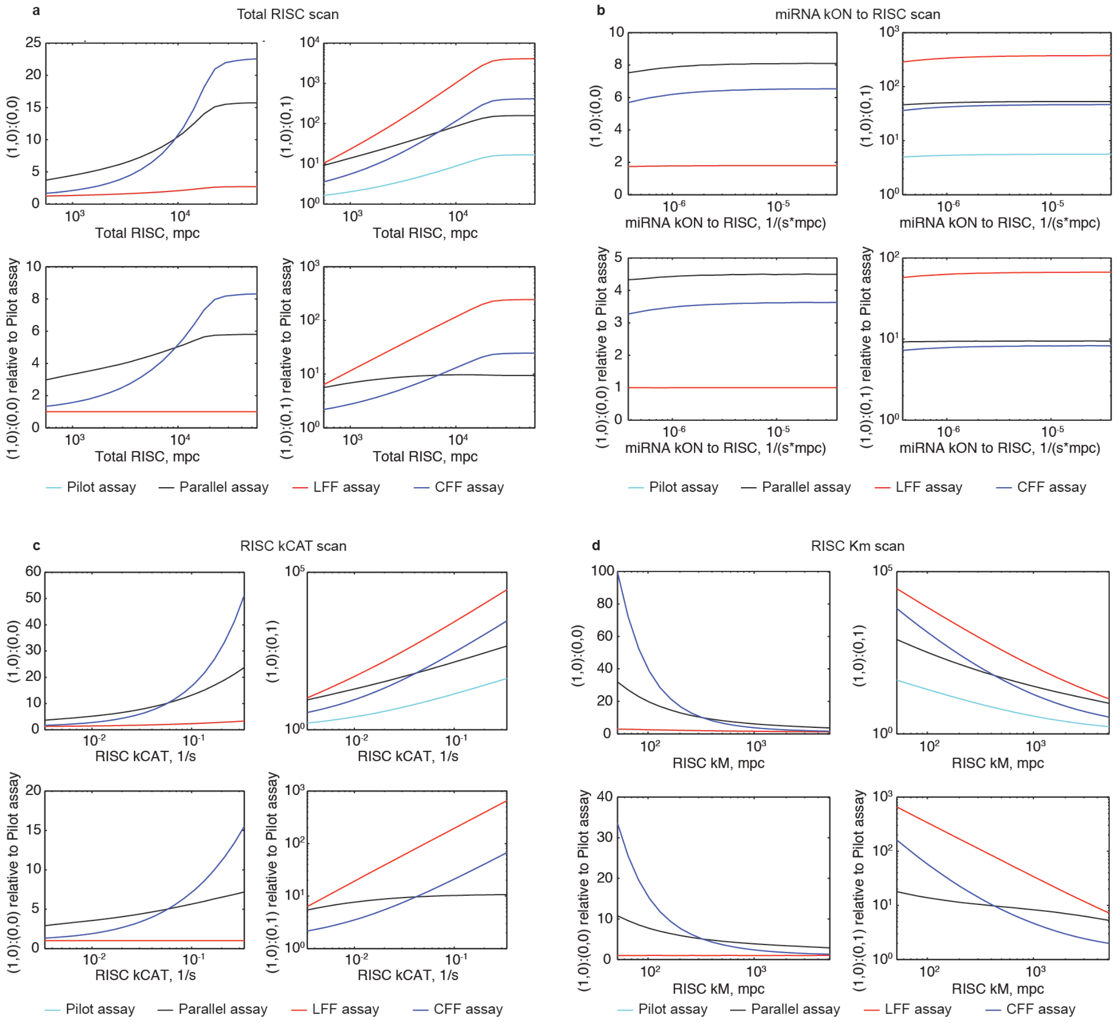

Supplementary Figure 6 We modeled miRNA related mRNA knockdown by Michaelis-Menten kinetics with a finite pool of RISC molecules. All parameters involved in these processes were scanned for their impact on the different assays' performances. The explanation of the plot data is in the legend to **Supplementary Figure 4**. The parameters scanned are as follows: (**a**) Total amount of RISC molecules present in the cell. (**b**) Rate constant of miRNA binding to RISC. (**c**) k_{CAT} of the loaded RISC-miRNA complex toward mRNA cleavage/sequestration. (**d**) K_M of the loaded RISC complex-induced mRNA cleavage/sequestration.

a

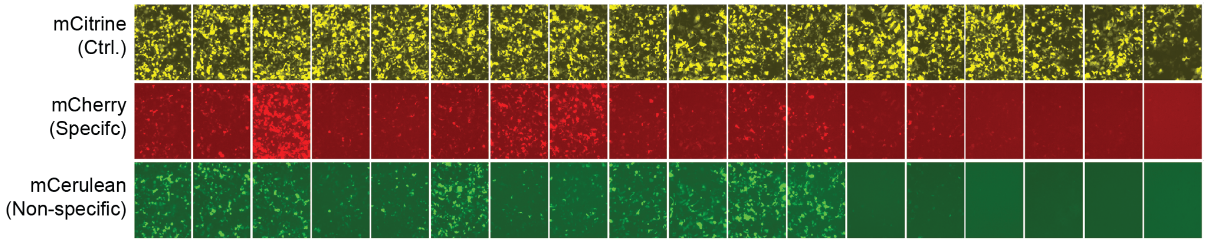

		LNA n.ctrl	1	2	3	4	5	6	mimic n.ctrl	7	8	9	10	11	12	13	14	15
miR-122	0.7	0.7	-1				-1	-1	0.7	+1						+1	+1	+1
miR-21	1	1		-1			-1	-1	1		+1						+1	+1
miR-20a	1	1			-1		-1	-1	1			+1					+1	+1
miR-FF4	low	low		high	high	-1	high	-1	low				+1				low	+1
miR-141	0	0							0					+1		+1	+1	+1
miR-146a	0	0							0						+1	+1	+1	+1

b

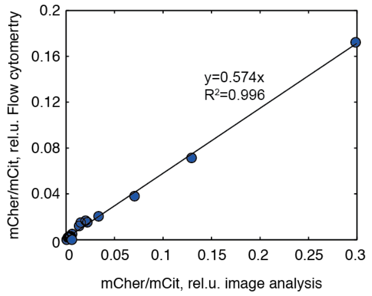

Supplementary Figure 7 (**a**) Representative microscopy images of transfections shown in **Figure 4b** for all perturbations used to characterize and validate the assay. The perturbations table is reproduced from **Figure 4b** for convenience. (**b**) Correlation between normalized mCherry values generated from flow cytometry data (**Fig. 4b**) with the values generated by our image-processing pipeline using microscopy (**Supplementary Fig. 7a**). Straight line indicates linear regression using least square fit. The slope and coefficient of determination (R^2) are displayed. Transfections are described in **Supplementary Table 8**. All bars and data points are mean ± s.d. of biological triplicates.

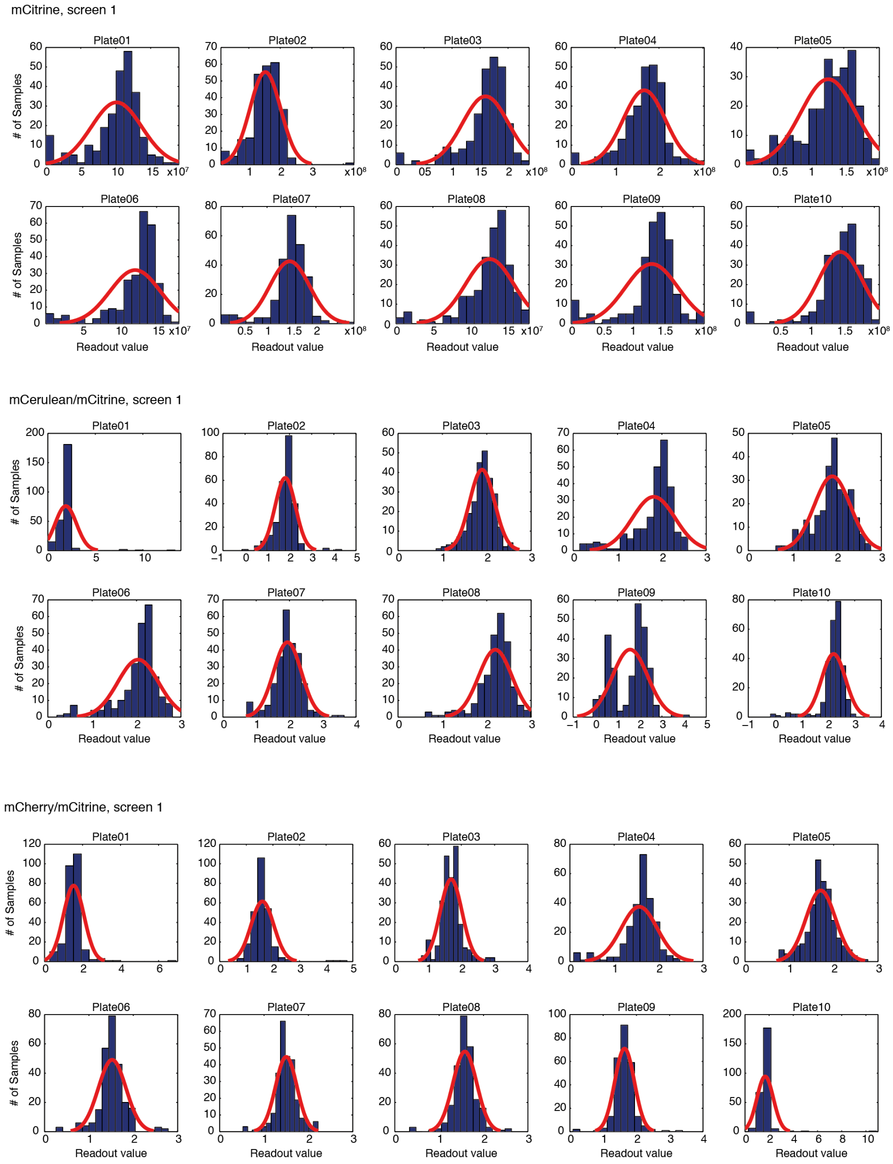

Supplementary Figure 8 Analysis of data distributions for screen 1. Different assay readouts corresponding to triplicate assay plates of the same compound plate are pooled together to build the histograms, which are further fitted to a normal distribution. The pooled readouts are used as reference distributions for hit identification (see **Methods**). Readouts and the compound storage plates are indicated. All the reference distributions are close to normal, except for mCerulean/mCitrine for plate09. Yet, most of these points are excluded based on mCitrine data. Transfections are described in **Supplementary Table 10**.

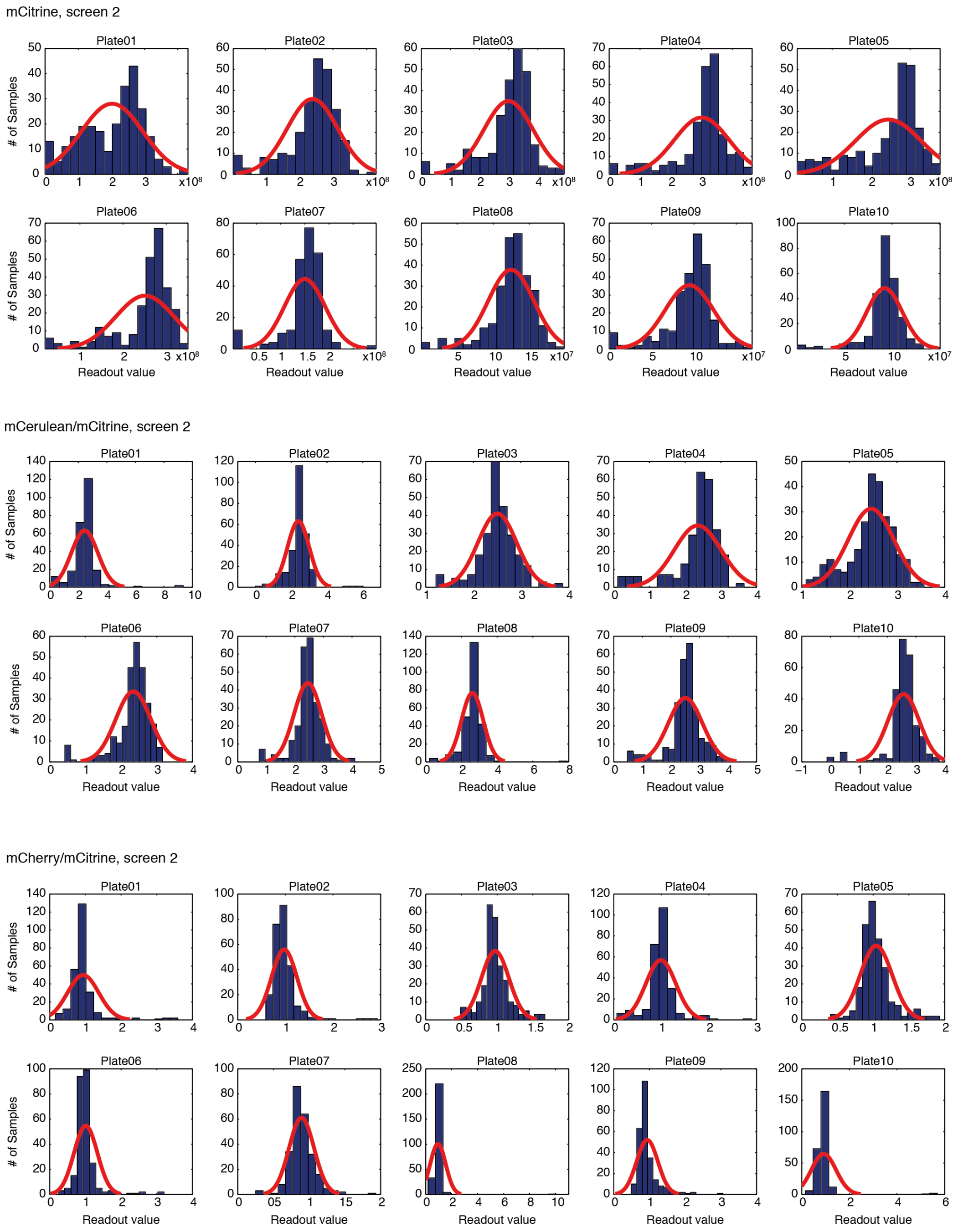

Supplementary Figure 9 Analysis of data distributions for screen 2. Different assay readouts corresponding to triplicate assay plates of the same compound plate are pooled together to build the histograms, which are further fitted to a normal distribution. The pooled readouts are used as reference distributions for hit identification (see **Methods**). Readouts as well as the compound storage plates are indicated. All of the reference distributions are close to normal, except for mCitrine for plate01. Transfections are described in **Supplementary Table 10**.

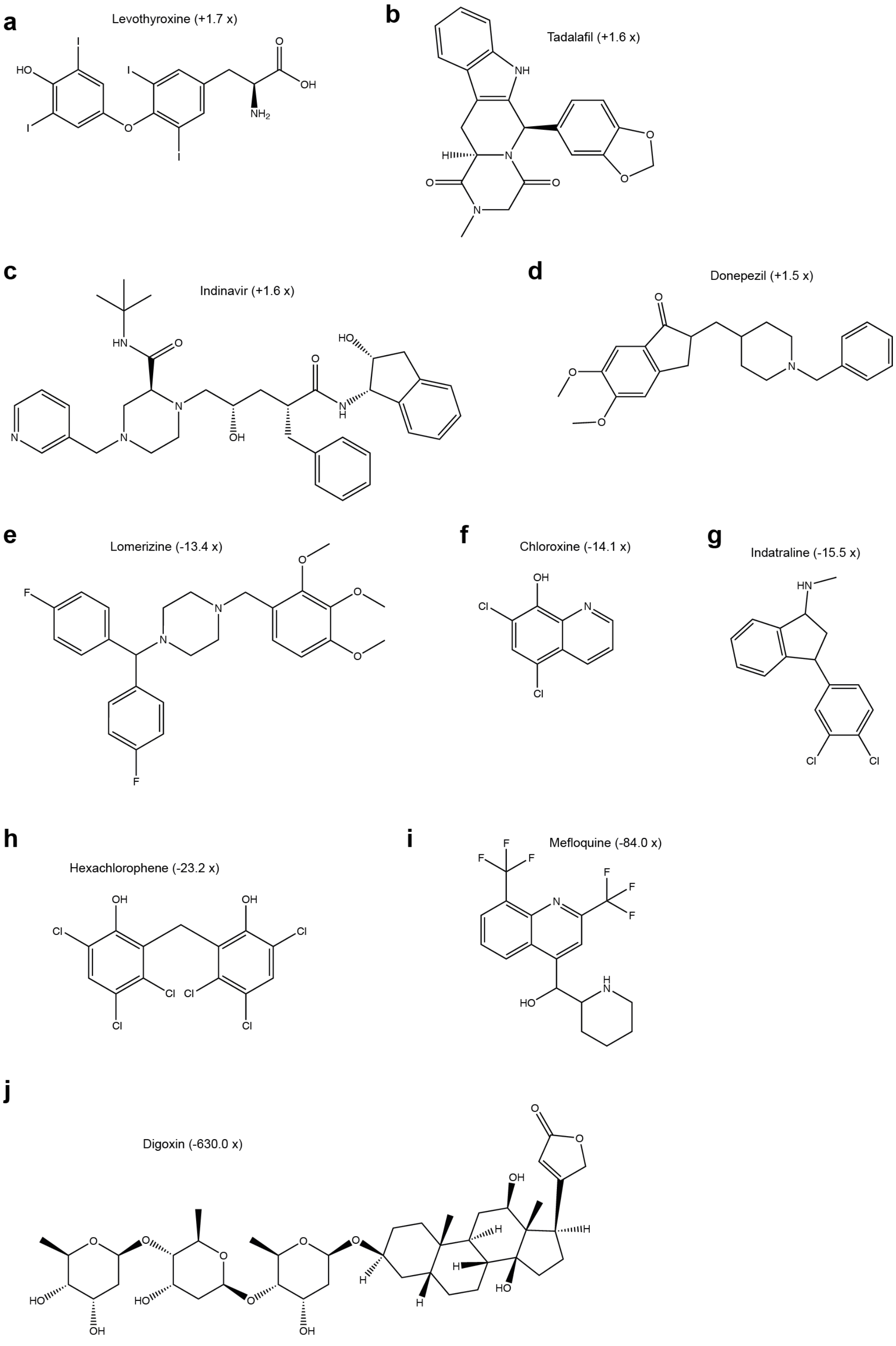

Supplementary Figure 10 (**a**)-(**j**) Chemical structures of compounds excluded based on the gene expression module (mCitrine) that were followed up in dose-response experiments. Numbers in brackets represent fold changes compared to plate mean averaged for the two screening runs.

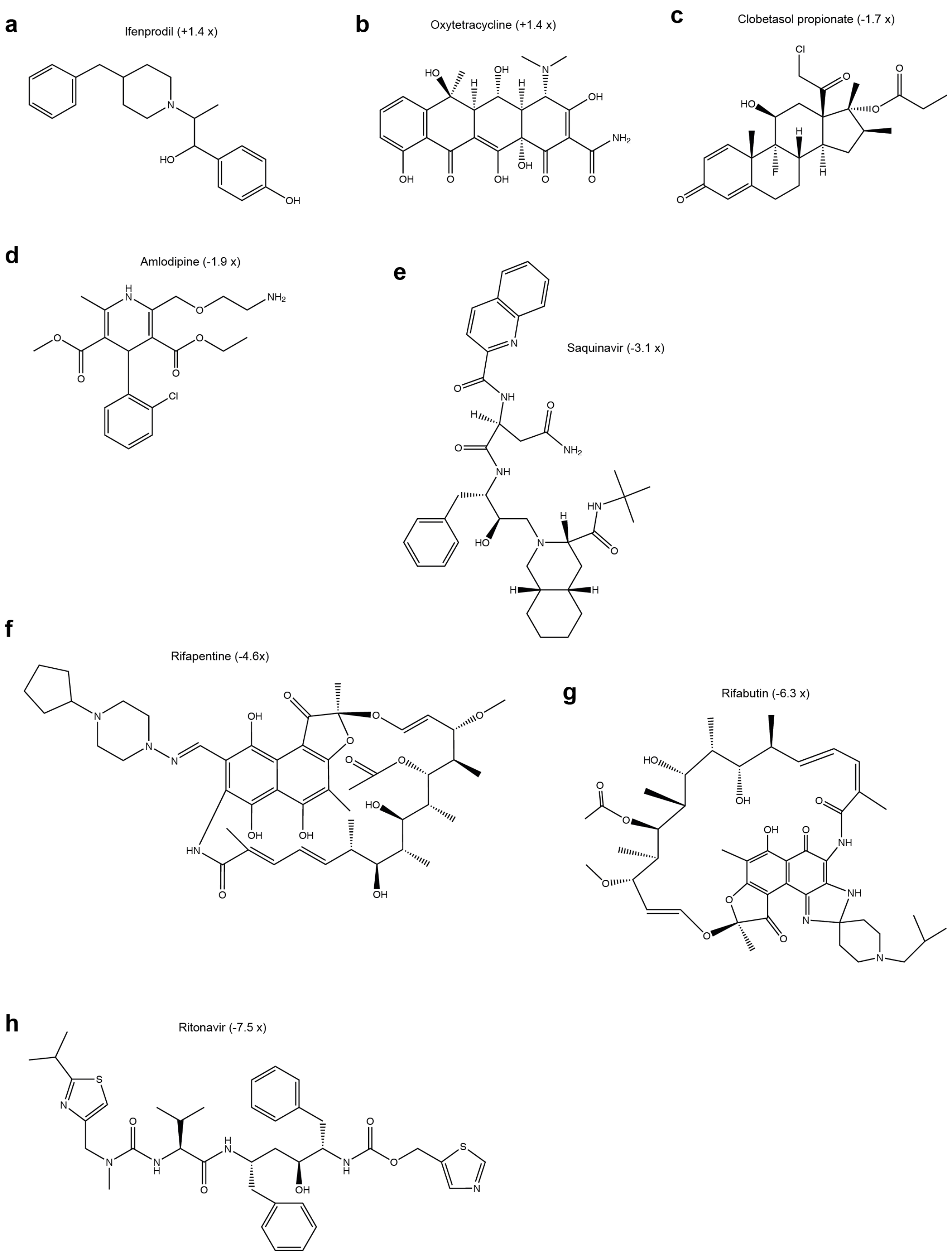

Supplementary Figure 11 (**a**)-(**h**) Chemical structures of compounds excluded based on the non-specific RNAi module readout (normalized mCerulean) that were followed up in dose-response experiments. Numbers in brackets represent fold changes compared to plate mean averaged for the two screening runs.

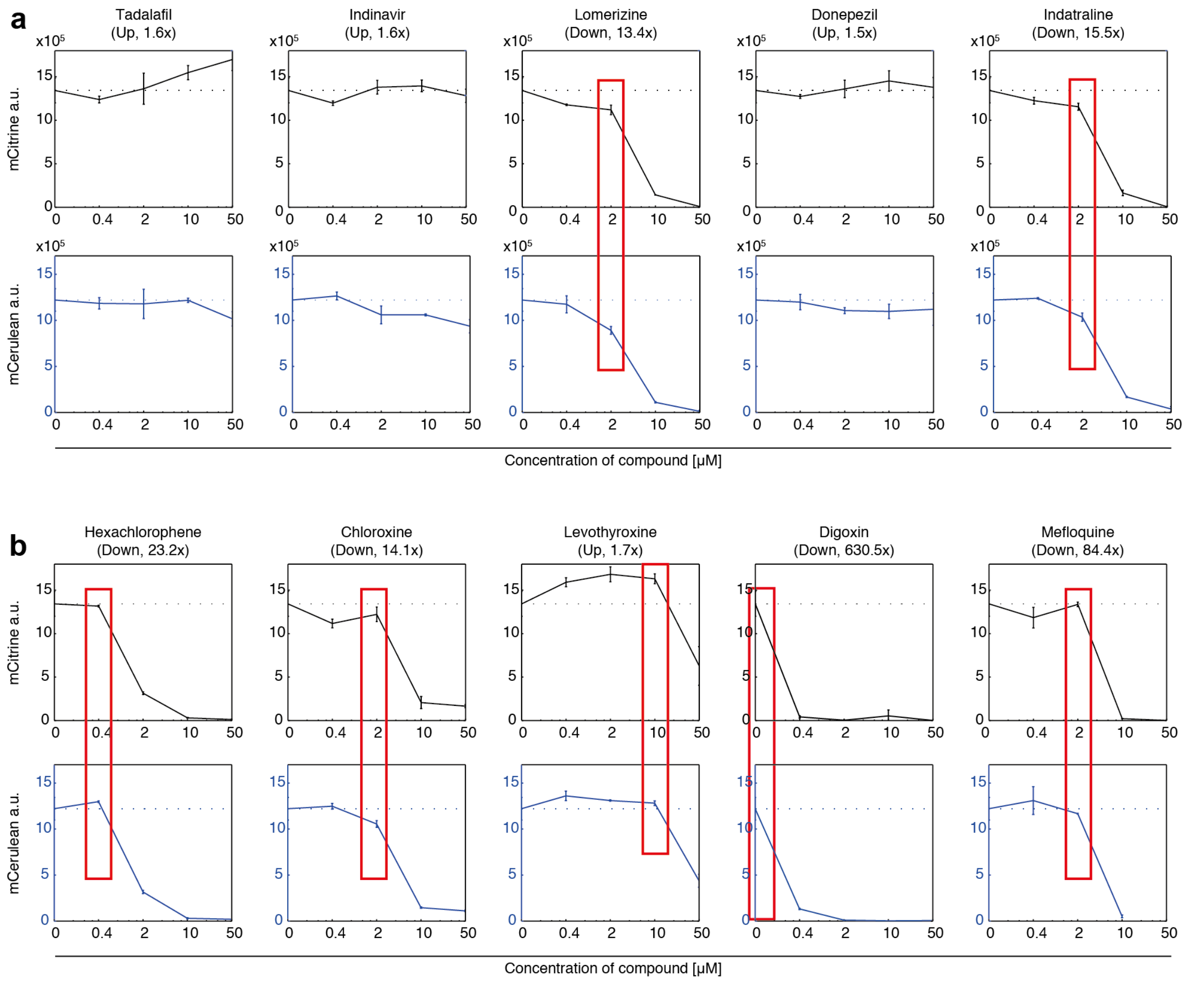

Supplementary Figure 12 Dose response characterization of gene expression module "hits" (mCitrine-based exclusion) with the full CFF screening assay as well as with the simple bidirectional reporter assay (See **Fig. 2a** for a scheme). (**a**) Gene expression readout (mCitrine) measured with the CFF assay (top row) and corresponding mCerulean readout for the simple bidirectional reporter assay (bottom row). mCerulean is without miRNA binding sites and overall protein load is smaller for this assay. Last concentration without toxic effects is indicated. (**b**) Gene expression readout (mCitrine) measured with the screening assay (top row) and corresponding mCerulean readout for the simple bidirectional reporter assay (bottom row). mCerulean is without miRNA binding sites and overall protein load is smaller for this assay. Last concentration without toxic effects is indicated. Transfections are described in **Supplementary Tables 37 and 38**. All data points and bars shown are mean ± s.d. of biological duplicates.

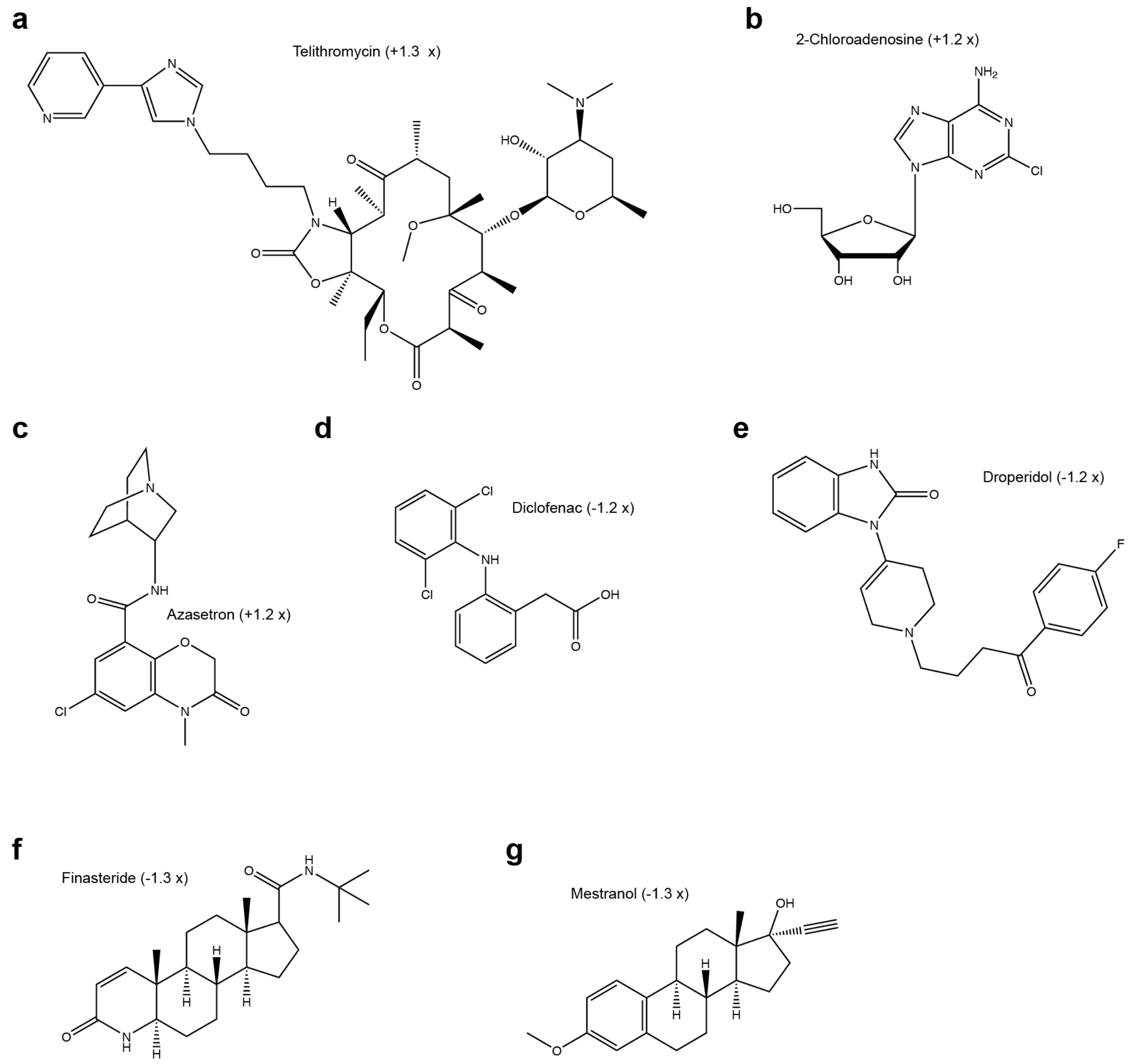

Supplementary Figure 13 (**a**)-(**g**) Chemical structures of compounds classified as specific hits (normalized mCherry). Numbers in brackets represent fold changes compared to plate mean averaged for the two screening runs.

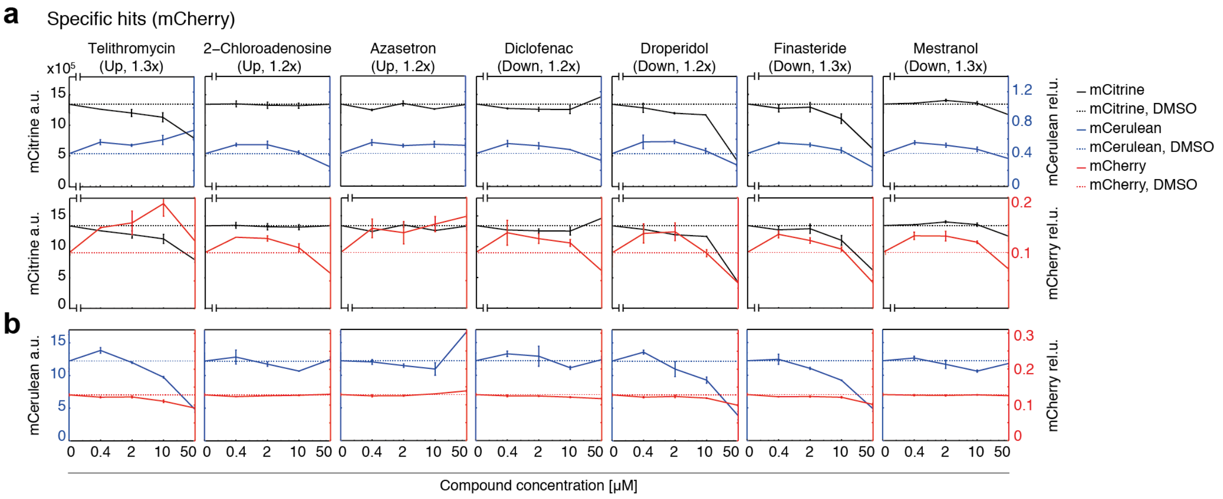

Supplementary Figure 14 Dose-response data for specific hits (mCherry based identification) measured with the circuit assay (**a**) as well as with simple bidirectional reporter assay (**b**). Transfections are described in **Supplementary Table 39**. All data points and bars shown are mean ± s.d. of biological duplicates.

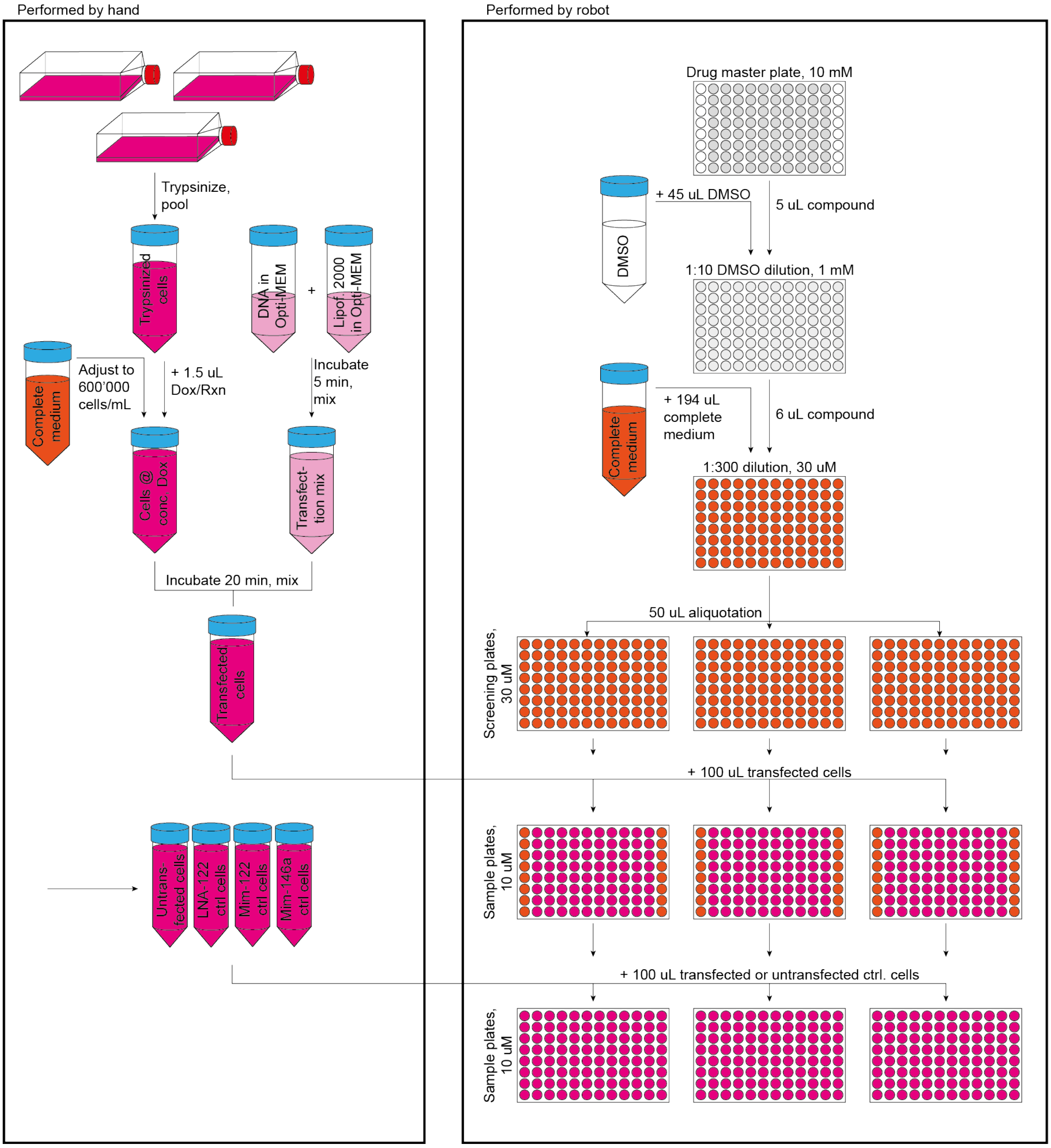

Supplementary Figure 15 Schematic representation of the transfection process for the screening experiments. Steps performed by hand and by robot are shown in separate boxes and the different dilution steps are indicated. Transfection mixes for the control wells are performed by hand as described for the sample ones.

Supplementary Tables

Supplementary Table 1 Z'-factors for the conditions displayed in **Figure 5**. The transfection details can be found in **Supplementary Table 8 and 9**. Values in shades of green indicate "excellent" assay performance (Z'>0.5), in yellow "acceptable" (0.5>Z'>0) and in red unsuitable for screening (Z'<0).

		mCerulean/mCitrine			mCherry/mCitrine		
		0.1 nM	1 nM	5 nM	0.1 nM	1 nM	5 nM
LNA-122	1				0.93	0.79	0.93
LNA-21	2	0.5	0.81	0.83			
LNA-20a	3	0.25	0.8	0.83			
LNA-FF4	4	-2.69	-0.1	0.67			
LNA-21/20a/122	5	0.49	0.85	0.83			
LNA-21/20a/122/FF4	6	0.47	0.78	0.65			
Mim-122	7				0.43	0.73	0.54
Mim-21	8	-6.19	-0.75	-0.82			
Mim-20a	9	-1.7	0.52	0.45			
siFF4	10	0.72	0.7	0.88			
Mim-141	11	0.73	0.68	0.87			
Mim-146a	12	0.78	0.71	0.88			
Mim-141/146a/122	13	0.77	0.71	0.88			
Mim-141/146a/122/21/20a	14	0.76	0.71	0.88			
Mim-141/146a/122/21/20a/FF4	15	0.77	0.7	0.88			

Supplementary Table 2 Plasmid Construction

**Ubi-Nos** (pDT7007, Junk-DNA): Described in Xie et al.[3]
**pcDNA3.1-IFP1.4**: Described in Shu et al.[4]
**CMV-PpLuc** (pZ003): Described in Xie et al.[5]
**CMV-RrLuc** (pZ005): Described in Xie et al.[5]
**CMV-Neomycin-miR-30 backbone-miR-FF3-miR-30 backbone** (pZ037): Described in Leisner et al.[6]
**AmCyan-TRE-DsRed-T21x4** (pZ072): Described in Xie et al.[3]
**AmCyan-TRE-DsRed-TFF5x4** (pZ073): Described in Xie et al.[3]
**CMV-rtTA-T21x4** (pZ090): Described in Xie et al.[3]

**CMV-rtTA-TFF5x4** (pZ091): Described in Xie et al.[3]
**CMV-rtTA-T21x4** (pZ090): Described in Xie et al.[3]
**TRE-LacI-TFF5x4** (pZ094): Described in Xie et al.[3]
**AmCyan-TRE-DsRed-T141x4** (pZ116): Described in Xie et al.[3]
**AmCyan-TRE-DsRed-T142-3px4** (pZ117): Described in Xie et al.[3]
**AmCyan-TRE-DsRed-T146ax4** (pZ118): Described in Xie et al.[3]
**AmCyan-TRE-DsRed-T17x4** (pZ145): Described in Xie et al.[3]
**AmCyan-TRE-DsRed-T30ax4** (pZ146a): Described in Xie et al.[3]
**TRE-LacI-T21x4-miR-FF4** (pZ224): Described in Xie et al.[3]
**TRE-LacI-TFF5x4-miR-FF4** (pZ225): Described in Xie et al.[3]
**CMV-IFP1.4** (pZ210): Commercial plasmid from Clontech #632441 was digested with NheI and XhoI and ligated with NheI and XhoI digested pcDNA3.1-IFP1.4.
**CMV-PIT2** (pMF206): Provided by Fussenegger Lab, described in Weber et al.[7]
**ERE-minCMV-SEAP** (pBP013): Provided by Fussenegger Lab, described in Weber et al.[8]
**PRE-minCMV-SEAP** (pBP031): Provided by Fussenegger Lab, described in Weber et al.[7]
**CMV-iRFP** (pCS0012): Plasmid from Addgene #31857, deposited by Vladislav Verkhusha, described in Filonov et al.[9]
**Ef1α-mCerulean** (pKH024): Described in Prochazka et al.[10]
**Ef1α-mCitrine** (pKH025): Described in Prochazka et al.[10]
**Ef1α-mCherry** (pKH026): Described in Prochazka et al.[10]
**MCS-TRE tight BI-MCS** (pIM001): Commercial plasmid from Clontech # 631068.
**MCS-TRE-mCherry** (pIM003): mCherry was PCR amplified using primers PR0534 and PR0535 from pKH026, digested with KpnI and MluI and ligated with KpnI and MluI digested pIM001.
**mCerulean-TRE-mCherry** (pIM002): mCerulean was PCR amplified using primers PR0522 and PR0563 form pKH024, digested using NdeI and EcoRI and ligated with NdeI and EcoRI digested pIM003.
**mCerulean-TRE-MCS** (pIM015): mCerulean was extracted from pIM003 by KpnI and MluI and was ligated with KpnI and MluI digested pIM001.
**TRE-LacI-TFF4x4** (pBA007): pZ094 was digested using NotI and SalI and was ligated with annealed oligos PR1093 and PR1094, coding for 4 repeats of inverse complement sequence of miR-FF4.

**ETR-2x-Hnf1-AmCyan-^miR-FF4^-2A-ET** (pBA026): Described in Angelici et al. (In preparation)
**PIR-2x-Hnf1-AmCyan-^miR-FF4^-2A-PIT2** (pBA065): Described in Angelici et al. (In preparation)
**CMV-tTA** (pBA166): Commercial plasmid from Clontech # 631069.
**CMV-DsRed-Express-PEST** (pNL69): Commercial plasmid from Clontech #632430 was digested using BglII and EcoRI and ligated with BglII and EcoRI digested PCR product of primers PR0913 and PR0914 on annealed oligos PR0876 and PR0877.
**AmCyan-TRE-DsRed-T145x4** (pBH0008): pZ072 was digested using NotI and SalI and was ligated with annealed oligos PR0077 and PR0078, coding for 4 repeats of inverse complement sequence of miR-145.
**AmCyan-TRE-DsRed-T24x4** (pBH0010): pZ072 was digested using NotI and SalI and was ligated with annealed oligos PR0081 and PR0082, coding for 4 repeats of inverse complement sequence of miR-24.
**AmCyan-TRE-DsRed-T375x4** (pBH0012): pZ072 was digested using NotI and SalI and was ligated with annealed oligos PR0085 and PR0086, coding for 4 repeats of inverse complement sequence of miR-375.
**AmCyan-TRE-DsRed-T196ax4** (pBH0014): pZ072 was digested using NotI and SalI and was ligated with annealed oligos PR0089 and PR0090, coding for 4 repeats of inverse complement sequence of miR-196a.
**CMV-DsRed-Express** (pBH0015): Commercial plasmid from Clontech #632430.
**CMV-ZsYellow** (pBH0016): Commercial plasmid from Clontech #632445.
**CMV-AmCyan** (pBH0017): Commercial plasmid from Clontech #632441.
**AmCyan-TRE-DsRed-T200cx4** (pBH0019): pZ072 was digested using NotI and SalI and was ligated with annealed oligos PR0107 and PR0108, coding for 4 repeats of inverse complement sequence of miR-200c.
**AmCyan-TRE-DsRed-T16x4** (pBH0020): pZ072 was digested using NotI and SalI and was ligated with annealed oligos PR0109 and PR0110, coding for 4 repeats of inverse complement sequence of miR-16.
**AmCyan-TRE-DsRed-Tlet7bx4** (pBH0021): pZ072 was digested using NotI and SalI and was ligated with annealed oligos PR0111 and PR0112, coding for 4 repeats of inverse complement sequence of let-7b.

**AmCyan-TRE-DsRed-T200cx4** (pBH0022): pZ072 was digested using NotI and SalI and was ligated with annealed oligos PR0113 and PR0114, coding for 4 repeats of inverse complement sequence of miR-23b.
**CMV-Neo-miR-30 Stem loop-miR-145 coding sequence** (pBH0024): pZ037 was digested using XhoI and EcoRI and ligated with annealed oligos PR0101 and PR0102, coding for a fully complementary lower miRNA stem, the miR-145 coding sequence and a miR-30 pPRIME[11] based loop sequence.
**mCerulean-TRE-mCherry-Spacer** (pBH0074): pBH0016 was digested using NotI and SalI to extract an 800 bp long Spacer. pIM002 was digested using NotI and SalI and was ligated with the 800 bp Spacer.
**mCerulean-TRE-mCherry-T130ax4** (pBH0075): pBH0074 was digested using NotI and SalI and was ligated with annealed oligos PR0626 and PR0627, coding for 4 repeats of inverse complement sequence of miR-130a.
**mCerulean-TRE-mCherry-T27bx4** (pBH0076): pBH0074 was digested using NotI and SalI and was ligated with annealed oligos PR0628 and PR0629, coding for 4 repeats of inverse complement sequence of miR-27b.
**mCerulean-TRE-mCherry-T125ax4** (pBH0077): pBH0074 was digested using NotI and SalI and was ligated with annealed oligos PR0630 and PR0631, coding for 4 repeats of inverse complement sequence of miR-125a.
**mCerulean-TRE-mCherry-T18ax4** (pBH0078): pBH0074 was digested using NotI and SalI and was ligated with annealed oligos PR0632 and PR0633, coding for 4 repeats of inverse complement sequence of miR-18a.
**mCerulean-TRE-mCherry-T7x4** (pBH0079): pBH0074 was digested using NotI and SalI and was ligated with annealed oligos PR0634 and PR0635, coding for 4 repeats of inverse complement sequence of miR-7.
**mCerulean-TRE-mCherry-T20ax4** (pBH0080): pBH0074 was digested using NotI and SalI and was ligated with annealed oligos PR0636 and PR0637, coding for 4 repeats of inverse complement sequence of miR-20a.

**mCerulean-TRE-mCherry-T21x4** (pBH0081): pBH0074 was digested using NotI and SalI and was ligated with NotI and SalI digested pZ072, coding for 4 repeats of inverse complement sequence of miR-21.
**mCerulean-TRE-mCherry-T21x4** (pBH0082): pBH0074 was digested using NotI and SalI and was ligated with NotI and SalI digested pBH0021, coding for 4 repeats of inverse complement sequence of let-7b.
**mCerulean-TRE-mCherry-T16x4** (pBH0083): pBH0074 was digested using NotI and SalI and was ligated with NotI and SalI digested pBH0020, coding for 4 repeats of inverse complement sequence of miR-16.
**mCerulean-TRE-mCherry-T24x4** (pBH0084): pBH0074 was digested using NotI and SalI and was ligated with NotI and SalI digested pBH0010, coding for 4 repeats of inverse complement sequence of miR-24.
**mCerulean-TRE-mCherry-TFF5x4** (pBH0091): pBH0074 was digested using NotI and SalI and was ligated with NotI and SalI digested pZ091, coding for 4 repeats of FF5 sequence.
**mCerulean-PRE-mCherry-800 bp Spacer** (pBH0107): The backbone encoding mCherry-Spacer-BB-mCerulean was amplified form pBH0074 using PR0646 and PR0647. PRE-Spacer-minCMV was amplified from pBP031 using primers PR0648 and PR0649. Spacer-minCMV was amplified from pBP031 using primers PR0650 and PR0651. All fragments were digested using BspQI and batch ligated to result in mCerulean-minCMV-Spacer-PRE-Spacer-minCMV-mCherry-Backbone.
**mCerulean-ERE-mCherry-800 bp Spacer** (pBH0111): The backbone encoding for mCherry-Spacer-BB-mCerulean was amplified form pBH0074 using PR0646 and PR0647. ERE-Spacer-minCMV was amplified from pBP013 using primers PR0648 and PR0649. Spacer-minCMV was created by annealing PR0644 and PR0645. All PCR products were digested using BspQI and batch ligated together with the annealing product to result in mCerulean-minCMV-Spacer-ERE-Spacer-minCMV-mCherry-Backbone.
**mCerulean-TRE-mCherry-T122x4** (pBH0112): pBH0074 was digested using NotI and SalI and was ligated with annealed oligos PR0720 and PR0721, coding for 4 repeats of inverse complement sequence of miR-122.

**mCerulean-TRE-mCherry-T122x1** (pBH0145): pBH0074 was digested using NotI and SalI and was ligated with annealed oligos PR0775 and PR0776, coding for 1 repeat of inverse complement sequence of miR-122.
**CMV-Exportin-5** (pBH0151): Plasmid from Addgene #12552, deposited by Ian Macara, described in Brownawell et al.[12]
**CAGop-ET-2A-Citrine-MCS** (pBH0152): A Gibson assembly was performed with the following fragments: pZ166 digested using AflII and AgeI. mCitrine PCR amplified form pKH025 using primers PR0784 and PR0789, ET PCR amplified from pBA026 using primers PR0782 and PR0783 and the MCS-pA was introduced by gBlock0013.
**CAGop-Citrine-2A-PIT2** (pBH0153): A Gibson assembly was performed with the following fragments: pZ166 digested using AflII and AgeI. mCitrine PCR amplified form pKH025 using primers PR0778 and PR0779, PIT2 PCR amplified from pBA065 using primers PR0780 and PR0781 and the MCS-pA was introduced by gBlock0013.
**mCerulean-TRE-RrLuc** (pBH0156): RrLuc was PCR amplified from pZ005 using primers PR0825 and PR0826 and digested using NotI and NheI. This product was ligated with EagI and NheI digested pIM015.
**PpLuc-TRE-RrLuc** (pBH0157): PpLuc was PCR amplified from pZ003 using primers PR0827 and PR0828 and digested using BsaI and BglII. This product was ligated with EcoRI and BglII digested pBH0156.
**PpLuc-TRE-RrLuc-TFF5x4** (pBH0159): pBH0157 was digested using NotI and SalI and ligated with NotI and SalI digested PCR product of primers PR0673 and PR0674 on pBH0145.
**PpLuc-TRE-RrLuc-T122x1** (pBH0160): pBH0157 was digested using NotI and SalI and ligated with NotI and SalI digested PCR product of primers PR0673 and PR0674 on pBH0091.
**PpLuc-TRE-RrLuc-T122x4** (pBH0161): T122x4 was PCR amplified form pBH0122 and digested using NotI and SalI. This was ligated with NotI and SalI digested pBH0157.

**CMV-Ubiquitin x4-ZsYellow** (pBH0173): Four ubiquitin coding fragments were produced the following: gBlock0014 was PCR amplified using primers PR0932 and PR0933 and digested with XbaI. gBlock0014 was PCR amplified using primers PR0934 and PR0935 and digested with AvrII and EcoRI. gBlock0014 was PCR amplified using primers PR0936 and PR0937 and digested with MfeI and PstI. gBlock0014 was PCR amplified using primers PR0938 and PR0939 and digested with NsiI. These four fragments were ligated and digested simultaneously with XbaI, AvrII, EcoRi, PstI, MfeI, NsiI in NEBuffer2 supplemented with ATP at 25 °C[13]. The largest ligation product was purified from gel and digested using SacI and BamHI to produce four repeats of ubiquitin. This fragment was then ligated into SacI and BamHI digested pBH0016.
**CMV-Neo-miR-30 Stem loop- anti-DGCR8 miRNA** (pBH0175): pBH0024 was digested using XhoI and EcoRI and ligated with annealed oligos PR0947 and PR0948 encoding for a DGCR8 targeting siRNA described in Chien et al.[14]
**mCerulean-ERE-mCherry-T122x4** (pBH0177): T122x4 was recived by digesting pBH0112 using NotI and SalI and was ligted into NotI and SalI digested pBH0111.
**CAGop-ET-2A-Citrine-MCS, killed XhoI site in ET** (pBH0178): In order to mutate the XhoI site within the coding region of ET, oligos PR0782 and PR0994 were annealed and filled in using phusion. This part was blunt-end ligated with the PCR product of PR0675 and PR0993 on pBH0152 and digested with AgeI and KpnI. The 986 bp band was purified form gel and ligated with the AgeI and KpnI digested pBH0152.
**CAGop-Citrine-2A-PIT2-MCS, killed XhoI site in PIT2** (pBH0179): In order to mutate the XhoI site in within the coding region of PIT2, two fragments were PCR amplified from pBH0153, once using primer PR0358 and PR0992, once with primers PR0793 and PR0091. These two fragments were blut-end ligated and digested using BglII and BspEI. The 593 bp band was gel extracted and ligated with BglII and BspEI digested pBH0153.
**CAGop-ET-2A-Citrine-TFF4x4** (pBH0180): TFF4x4 was extracted from pBA007 by NotI and SalI digestion to be ligated with NotI and PspXI digested pBH0178.
**CAGop-Citrine-2A-PIT2-TFF4x4** (pBH0181): TFF4x4 was extracted from pBA007 by NotI and SalI digestion to be ligated with NotI and PspXI digested pBH0179.
**mCerulean-PRE-mCherry-T122x4** (pBH0182): T122x4 was received by digesting pBH0112 using NotI and SalI and was ligted into NotI and SalI digested pBH0107.

**TRE-Ubiquitinx4-mCherry** (pBH0183): pIM003 was digested using KpnI, dephosphorylated and ligated with BstXI digested PCR product of primers PR0940 and PR0941 on pBH0173.
**TRE-Ubiquitinx4-mCherry-PEST** (pBH0184): pBH0183 was digested using BsrGI and HindIII and ligated with BsrGI and HindIII digested PCR product of PR0995 and PR0996 on pNL_69.
**mCerulean-TRE-mCherry-T145x4** (pBH0193): pBH0074 was digested using NotI and SalI and was ligated with NotI and SalI digested pBH0008, coding for 4 repeats of inverse complement sequence of miR-145.
**mCerulean-TRE-mCherry-T375x4** (pBH0194): pBH0074 was digested using NotI and SalI and was ligated with NotI and SalI digested pBH0012, coding for 4 repeats of inverse complement sequence of miR-375.
**mCerulean-TRE-mCherry-T146ax4** (pBH0195): pBH0074 was digested using NotI and SalI and was ligated with NotI and SalI digested pZ118, coding for 4 repeats of inverse complement sequence of miR-146a.
**β-actin-op-ET-2A-Citrine-TFF4x4** (pBH0197): A MCS in the backbone of pBH0180 and its neighboring CMV early enhancer were deleted by SnaBI digestion and subsequent self-ligation.
**β-actin-op-Citrine-2A-PIT2-TFF4x4** (pBH0198): A MCS in the backbone of pBH0181 and its neighboring CMV early enhancer were deleted by SnaBI digestion and subsequent self-ligation.
**β-actin-op-ET-2A-Citrine-TFF4x4-T145x4** (pBH0199): T145x4 was PCR amplified from pBH0193 using primers PR0673 and PR0674 and digested using MluI and SalI to be ligated with AscI and BsmBI digested pBH0197.
**β-actin-op-Citrine-2A-PIT2-TFF4x4-T145x4** (pBH0200): T145x4 was PCR amplified from pBH0193 using primers PR0673 and PR0674 and digested using MluI and SalI to be ligated with AscI and BsmBI digested pBH0198.
**CMV-rtTA-T20ax4-T130ax4** (pBH0201): NotI-T20ax4-SgrDI-AarI-T130a-AarI-HindIII was obtained by annealing oligo PR1069 with PR1070 and PR1071 with PR1072, followed by subsequent ligation and gel purification. This fragment was then ligated with NotI and HindIII digested pZ090.
**TRE-LacI-T20ax4-T130ax4-miR-FF4** (pBH0202): Target insert was recived as for pBH0201 and ligated with NotI and HindIII digested pZ224.

**β-actin-op-ET-2A-Citrine-TFF4x4-T375x4-T145x4** (pBH0203): T375x4 was PCR amplified from pBH0194 using primers PR0673 and PR0674 and digested using MluI and SalI to be ligated with PaeR7I and MluI digested pBH0201.
**β-actin-op-Citrine-2A-PIT2-TFF4x4-T375x4-T145x4** (pBH0204): T375x4 was PCR amplified from pBH0194 using primers PR0673 and PR0674 and digested using MluI and SalI to be ligated with PaeR7I and MluI digested pBH0200.
**β-actin-op-ET-2A-Citrine-TFF4x4-T146ax4-T375x4-T145x4** (pBH0206): T146ax4 was PCR amplified from pBH0195 using primers PR0673 and PR0674 and digested using NheI and SalI to be ligated with SpeI and XhoI digested pBH0203.
**β-actin-op-ET-2A-Citrine-TFF4x4-T146ax4-T375x4-T145x4** (pBH0207): T146ax4 was PCR amplified from pBH0195 using primers PR0673 and PR0674 and digested using NheI and SalI to be ligated with SpeI and XhoI digested pBH0204.
**CMV-rtTA-T20ax4** (pBH0211): pBH0201 was digested using AarI, Klenow Large Fragment treated and self-ligated.
**TRE-LacI-T20ax4-miR-FF4** (pBH0212): pBH0202 was digested using AarI, Klenow Large Fragment treated and self-ligated.
**CMV-rtTA-T130ax4** (pBH0213): pBH0201 was digested using SgrDI and NotI, Klenow Large Fragment treated and self-ligated.
**CAGop-ET-2A-Citrine-TFF4x4-T146ax4-T375x4-T145x4** (pBH0219): ET-2A-Citrine-Tbox was extracted from pBH0206 by digesting with XbaI and BsmBI and ligate with XbaI and BsmBI digested pBH0180.
**CAGop-Citrine-2A-PIT2-TFF4x4-T146ax4-T375x4-T145x4** (pBH0220): In order to get Citrine-2A-PIT2-Tbox pBH0207 is digested using XbaI and BsmBI. This fragment is ligated with pBH0180 digested using XbaI and BsmBI as well.
**CMV-rtTA-T20ax1** (pBH0225): pBH0201 was digested using NotI and SalI and ligated with annealed oligos PR1277 and PR1278, coding for 1 repeat of inverse complement sequence of miR-20a.
**CMV-rtTA-T20ax2** (pBH0226): pBH0201 was digested using NotI and SalI and ligated with annealed oligos PR1279 and PR1280, coding for 2 repeats of inverse complement sequence of miR-20a.

**CAGop-ET-TFF4x4-T146ax4-T375x4-T145x4** (pBH0229): ET was extracted from pBH0219 by PCR amplification with primers PR0830 and PR1307. This fragment was digested using AgeI and BsrGI and ligated with pBH0219 digested by AgeI and BsrGI.
**CAGop-PIT2-TFF4x4-T146ax4-T375x4-T145x4** (pBH0230): PIT2-Target-box was extracted from pBH0220 by PCR amplification with primers PR0793 and PR1308. This fragment was digested using AgeI and BsmBI and ligated with pBH0220 digested by AgeI and BsmBI.
**CAGop-ET-TFF4x4-T146ax4-T141x4** (pBH0231): pBH0219 was digested using MluI and BsmBI and ligated with annealed oligos PR1320 and PR1321, which are coding for 4 repeats of inverse complement sequence of miR-141.
**CAGop-Citrine-2A-PIT2-TFF4x4-T146ax4-T141x4** (pBH0232): pBH0220 was digested using MluI and BsmBI and ligated with annealed oligos PR1320 and PR1321, which are coding for 4 repeats of inverse complement sequence of miR-141.
**CAGop-PIT2-TFF4x4-T146ax4-T141x4** (pBH0234): pBH0230 was digested using MluI and BsmBI and ligated with annealed oligos PR1320 and PR1321, which are coding for 4 repeats of inverse complement sequence of miR-141.
**CAGop-Citrine-TFF4x4-T146ax4-T375x4-T145x4** (pBH0235): In order to remove PIT2 or ET to get a Citrine only construct pBH0229 and pBH0232 were digested using XbaI and BsrGI, which results after proper insert/backbone choice in the wanted product.
**T141x4-T146ax4-mCerulean-PRE-mCherry-T122x4** (pBH0246): T141x4-T146ax4 is extracted from pBH0231 by PCR using primers PR1442 and PR1443. This fragment digested using PspOMI and XbaI is ligated with pBH0182 cut with the same restriction enzymes.
**CAGop-ZsYellow-TFF4x4-T146ax4-T375x4-T145x4** (pBH0247): ZsYellow was extracted from pBH0016 by PCR amplification using primers PR1125 and PR1451 and digested using XmaI and BsaI. This fragment was ligated with pBH0235 digested with AgeI and BsrGI.
**CAGop-ET-2A-Citrine-T146ax4-T375x4-T145x4-TFF4x4** (pBH0249): T146ax4-T375x4-T145x4 was extracted from pBH0229 by PCR using primer PR1442 and PR1492 and digested with BsaI. Then ligated with annealed oligos PR1493 and PR1494 coding for 4 repeats of inverse complement sequence of miR-FF4 to result in T146ax4-T375x4-T145x4-TFF4x4. This was digested with HindIII and ligated with HindIII and BsmBI digested pBH0219.

**CAGop-ET-2A-Citrine-T146ax4-T141x4-TFF4x4** (pBH0250): T141x4-T146ax4 was extracted from pBH0231 by PCR using primers PR1442 and PR1492 and digested with BsaI. Then ligated with annealed oligos PR1493 and PR1494, coding for 4 repeats of inverse complement sequence of miR-FF4 to result in T141x4-T146ax4-TFF4x4. This was digested with HindIII and ligated with HindIII and BsmBI digested pBH0219.
**CAGop-ET-T146ax4-T141x4-TFF4x4** (pBH0252): Target box T141x4-T146ax4-TFF4x4 constructed as for pBH0250, but ligation with HindIII and BsmBI digested pBH0229.
**CAGop-Citrine-2A-PIT2-T146ax4-T141x4-TFF4x4** (pBH0254): Target box T141x4-T146ax4-TFF4x4 constructed as for pBH0250, but ligation with HindIII and BsmBI digested pBH0232.
**CAGop-PIT-T146ax4-T375x4-T145x4-TFF4x4** (pBH0255): Target box T146ax4-T375x4-T145x4 was constructed as for pBH0249, but ligation with HindIII and BsmBI digested pBH0234.
**CAGop-PIT2-T146ax4-T141x4-TFF4x4** (pBH0256): Target box T141x4-T146ax4-TFF4x4 constructed as for pBH0250, but ligation with HindIII and BsmBI digested pBH0234.
**CAGop-ZsYellow-T146ax4-T141x4-TFF4x4** (pBH0260): Target box T141x4-T146ax4-TFF4x4 constructed as for pBH0250, but ligation with HindIII and BsmBI digested pBH0247.
**TFF4x4-T145x4-T375x4-T146ax4-mCerulean-PRE-mCherry-T122x4** (pBH0263): TFF4x4-T145x4-T375x4-T146ax4 is extracted from pBH0249 by PCR using primers PR1441 and PR1443. This fragment digested using PspOMI and XbaI is ligated with pBH0182 cut with the same restriction enzymes.
**TFF4x4-T141x4-T146ax4-mCerulean-PRE-mCherry-T122x4** (pBH0264): TFF4x4-T141x4-T146ax4 is extracted from pBH0250 by PCR using primers PR1441 and PR1443. This fragment digested using PspOMI and XbaI is ligated with pBH0182 cut with the same restriction enzymes.
**Lac-op free Junk-DNA Ubi-Nos** (pBH265): MauBI and BspQI flank the lacO sites in pDT7004. These enymes were used to digest the plasmid before Klenow Large Fragment treatment and self-ligation.
**mCerulean-TRE-mCherry-TFF4x4** (pBH0266): pBH0074 was digested using NotI and SalI and was ligated with annealed oligos PR1493 and PR1494, coding for 4 repeats of inverse complement sequence of miR-FF4.

**mCerulean-TRE-mCherry-T141x4** (pBH0267): pBH0074 was digested using NotI and SalI and was ligated with NotI and SalI digested pZ116, coding for 4 repeats of inverse complement sequence of miR-141.
**mCerulean-TRE-mCherry-23bx4** (pBH0273): pBH0074 was digested with NotI and SalI and ligated with NotI and SalI digested PCR product of primers PR0673 and PR0674 on pBH0022.
**TRE-Ubiquitinx4-mCherry-PEST-T122x4** (pBH0277): pBH0184 was digested using NotI and EcoRV and ligated with NotI and EcoRV digested PCR product of primers PR0673 and PR0674 on pBH0112.
**TFF4x4-T141x4-T146ax4-mCerulean-PRE-mCherry-T23bx4** (pBH0278): pBH0264 was digested with NheI and SalI and ligated with NheI and SalI digested pBH0273.
**mCerulean-TRE-Ubx4-mCherry-T122x1** (pBH0279): pBH0145 was digested using NotI and XhoI and ligated with NotI and XhoI digested pBH0183.
**mCerulean-TRE-Ubx4-mCherry-PEST-T122x1** (pBH0280): pBH0145 was digested using NotI and XhoI and ligated with NotI and XhoI digested pBH0184.
**mCerulean-TRE-Ubx4-mCherry-T122x4** (pBH0281): pBH0279 was digested using NotI and AseI and ligated with NotI and AseI digested pBH0277.
**mCerulean-TRE-Ubx4-mCherry-PEST-T122x4** (pBH0282): pBH0280 was digested using NotI and AseI and ligated with NotI and AseI digested pBH0277.
**mCerulean-TRE-Ubx4-mCherry-PEST-TFF5x4** (pBH0284): pBH0282 was digested using NotI and AseI and ligated with NotI and AseI digested pBH0091.
**mCerulean-TRE-mCherry-PEST-T122x4** (pBH0286): pBH0282 was digested using AgeI and NheI, treated with T4-DNA polymerase and ligated to close on itself.
**mCerulean-TRE-mCherry-PEST-TFF5x4** (pBH0287): pBH0284 was digested using AgeI and NheI, treated with T4-DNA polymerase and ligated to close on itself.
**TFF4x4-T141x4-T146ax4-mCerulean-PRE-mCherry-Tlet7bx4** (pBH0288): pBH0264 was digested with BsaI and NheI and ligated with BsaI NheI digested pBH0082.

Supplementary Table 3 List of primers used

PR0077	GGCCGCAAAAGGGATTCCTGGGAAAACTGGACAGGGATTCCTGGGAAAACTGGACAG GGATTCCTGGGAAAACTGGACAGGGATTCCTGGGAAAACTGGACG
PR0078	TCGACGTCCAGTTTTCCCAGGAATCCCTGTCCAGTTTTCCCAGGAATCCCTGTCCAGTT TTCCCAGGAATCCCTGTCCAGTTTTCCCAGGAATCCCTTTTGC
PR0081	GGCCGCAAACTGTTCCTGCTGAACTGAGCCACTGTTCCTGCTGAACTGAGCCACTGTT CCTGCTGAACTGAGCCACTGTTCCTGCTGAACTGAGCCAG
PR0082	TCGACTGGCTCAGTTCAGCAGGAACAGTGGCTCAGTTCAGCAGGAACAGTGGCTCAG TTCAGCAGGAACAGTGGCTCAGTTCAGCAGGAACAGTTTGC
PR0085	GGCCGCAAATCACGCGAGCCGAACGAACAAATCACGCGAGCCGAACGAACAAATCAC GCGAGCCGAACGAACAAATCACGCGAGCCGAACGAACAAAG
PR0086	TCGACTTTGTTCGTTCGGCTCGCGTGATTTGTTCGTTCGGCTCGCGTGATTTGTTCGTT CGGCTCGCGTGATTTGTTCGTTCGGCTCGCGTGATTTGC
PR0089	GGCCGCAAACCCAACAACATGAAACTACCTACCCAACAACATGAAACTACCTACCCAA CAACATGAAACTACCTACCCAACAACATGAAACTACCTAG
PR0090	TCGACTAGGTAGTTTCATGTTGTTGGGTAGGTAGTTTCATGTTGTTGGGTAGGTAGTTT CATGTTGTTGGGTAGGTAGTTTCATGTTGTTGGGTTTGC
PR0091	TGAAGGGCGAGATCCACA
PR0101	TCGAGGAGCATTCCTGGTCCAGTTTTCCCAGGAATCCCTTAGTAAGAGGGCAACCTTA AGGGATTCCTATGAAAACTGAATCAGGAGTGTTTG
PR0102	AATTCAAACACTCCTGATTCAGTTTTCATAGGAATCCCTTAAGGTTGCCCTCTTACTAAG GGATTCCTGGGAAAACTGGACCAGGAATGCTCC
PR0107	GGCCGCAAATCCATCATTACCCGGCAGTATTATCCATCATTACCCGGCAGTATTATCCA TCATTACCCGGCAGTATTATCCATCATTACCCGGCAGTATTAG
PR0108	TCGACTAATACTGCCGGGTAATGATGGATAATACTGCCGGGTAATGATGGATAATACTG CCGGGTAATGATGGATAATACTGCCGGGTAATGATGGATTTGC
PR0109	GGCCGCAAACGCCAATATTTACGTGCTGCTACGCCAATATTTACGTGCTGCTACGCCA ATATTTACGTGCTGCTACGCCAATATTTACGTGCTGCTAG
PR0110	TCGACTAGCAGCACGTAAATATTGGCGTAGCAGCACGTAAATATTGGCGTAGCAGCAC GTAAATATTGGCGTAGCAGCACGTAAATATTGGCGTTTGC
PR0111	GGCCGCAAAAACCACACAACCTACTACCTCAAACCACACAACCTACTACCTCAAACCA CACAACCTACTACCTCAAACCACACAACCTACTACCTCAG
PR0112	TCGACTGAGGTAGTAGGTTGTGTGGTTTGAGGTAGTAGGTTGTGTGGTTTGAGGTAGT AGGTTGTGTGGTTTGAGGTAGTAGGTTGTGTGGTTTTTGC
PR0113	GGCCGCAAAGGTAATCCCTGGCAATGTGATGGTAATCCCTGGCAATGTGATGGTAATC CCTGGCAATGTGATGGTAATCCCTGGCAATGTGATG
PR0114	TCGACATCACATTGCCAGGGATTACCATCACATTGCCAGGGATTACCATCACATTGCCA GGGATTACCATCACATTGCCAGGGATTACCTTTGC
PR0358	AAGGAGGACGGCAACATCCTG
PR0522	CGGCCATATGTTACTTGTACAGCTCGTCCATG

PR0534	CAATGCTAGCGCCTCAGACAGTGGTTCAAAG
PR0535	CAGCCACCACCTTCTGATAGG
PR0563	GTACAGAATTCGCCACCATGGTGAGCAAGG
PR0626	GGCCGCAAAATGCCCTTTTAACATTGCACTGATGCCCTTTTAACATTGCACTGATGCCCTTTTAACATTGCACTGATGCCCTTTTAACATTGCACTGG
PR0627	TCGACCAGTGCAATGTTAAAAGGGCATCAGTGCAATGTTAAAAGGGCATCAGTGCAATGTTAAAAGGGCATCAGTGCAATGTTAAAAGGGCATTTTGC
PR0628	GGCCGCAAAGCAGAACTTAGCCACTGTGAAGCAGAACTTAGCCACTGTGAAGCAGAACTTAGCCACTGTGAAGCAGAACTTAGCCACTGTGAAG
PR0629	TCGACTTCACAGTGGCTAAGTTCTGCTTCACAGTGGCTAAGTTCTGCTTCACAGTGGCTAAGTTCTGCTTCACAGTGGCTAAGTTCTGCTTTGC
PR0630	GGCCGCAAATCACAGGTTAAAGGGTCTCAGGGATCACAGGTTAAAGGGTCTCAGGGATCACAGGTTAAAGGGTCTCAGGGATCACAGGTTAAAGGGTCTCAGGGAG
PR0631	TCGACTCCCTGAGACCCTTTAACCTGTGATCCCTGAGACCCTTTAACCTGTGATCCCTGAGACCCTTTAACCTGTGATCCCTGAGACCCTTTAACCTGTGATTTGC
PR0632	GGCCGCAAACTATCTGCACTAGATGCACCTTACTATCTGCACTAGATGCACCTTACTATCTGCACTAGATGCACCTTACTATCTGCACTAGATGCACCTTAG
PR0633	TCGACTAAGGTGCATCTAGTGCAGATAGTAAGGTGCATCTAGTGCAGATAGTAAGGTGCATCTAGTGCAGATAGTAAGGTGCATCTAGTGCAGATAGTTTGC
PR0634	GGCCGCAAAACAACAAAATCACTAGTCTTCCAACAACAAAATCACTAGTCTTCCAACAACAAAATCACTAGTCTTCCAACAACAAAATCACTAGTCTTCCAG
PR0635	TCGACTGGAAGACTAGTGATTTTGTTGTTGGAAGACTAGTGATTTTGTTGTTGGAAGACTAGTGATTTTGTTGTTGGAAGACTAGTGATTTTGTTGTTTTGC
PR0636	GGCCGCAAACTACCTGCACTATAAGCACTTTACTACCTGCACTATAAGCACTTTACTACCTGCACTATAAGCACTTTACTACCTGCACTATAAGCACTTTAG
PR0637	TCGACTAAAGTGCTTATAGTGCAGGTAGTAAAGTGCTTATAGTGCAGGTAGTAAAGTGCTTATAGTGCAGGTAGTAAAGTGCTTATAGTGCAGGTAGTTTGC
PR0644	GGCCCAGGCGATCTGACGGTTCACTAAACGAGCTCTGCTTATATAGGCCTCCCACCGTACACGCCTACTCGACCCGGGTACCGAGCTCGAATTACGATCCTGCAGG
PR0645	TCGCCTGCAGGATCGTAATTCGAGCTCGGTACCCGGGTCGAGTAGGCGTGTACGGTGGGAGGCCTATATAAGCAGAGCTCGTTTAGTGAACCGTCAGATCGCCTGG
PR0646	GCACCATCCTGCTCTTCCGAGCGCCACCATGGTGAGCAAGGGCGAGGAGGATAACATG
PR0647	GCACCATCCTGCTCTTCCGCCGCCACCATGGTGAGCAAGGGCGAGGAGCTGTTCAC
PR0648	GCACCATCCTGCTCTTCCGCGCTGTACAGCGTATGGGAATCTCTTG
PR0649	GCACCATCCTGCTCTTCCCTCCAGGCGATCTGACGGTTCACTAAAC
PR0650	GCACCATCCTGCTCTTCCCGCTATTTCCAAGGAGCCTGCAGGATCGTCGAGCTCGGTAC
PR0651	GCACCATCCTGCTCTTCCGGCGATCTGACGGTTCACTAAACGAG

PR0673	TCCCACAACGAGGACTACAC
PR0674	CGAGTCAGTGAGCGAGGAAG
PR0675	AACTTGTGGCCGTTTACGTC
PR0720	GGCCGCAAAACAAACACCATTGTCACACTCCACAAACACCATTGTCACACTCCACAAAC ACCATTGTCACACTCCACAAACACCATTGTCACACTCCAG
PR0721	TCGACTGGAGTGTGACAATGGTGTTTGTGGAGTGTGACAATGGTGTTTGTGGAGTGTG ACAATGGTGTTTGTGGAGTGTGACAATGGTGTTTGTTTTGC
PR0778	ATTACGGCCGCTAGCGCTACCGGACTCAGATCCACCGGTTCGCCACCATGGTGAGCA AGGGCGAGGAG
PR0779	CTTCCCCTGCCCTCGGCTCTGGTACCCTTGTACAGCTCGTCCATGCCGAGAGTGATCC
PR0780	GCATGGACGAGCTGTACAAGGGTACCAGAGCCGAGGGCAGGGGAAGTCTTCTAACAT GC
PR0781	TTGCACTAGTCGCGTGACTCGAGTTCGTGTTGGCGGCCGCCCATGTACTTGGGAAGCT TCCTTAGGAGCTGATCTGACTCAGCAGGGCTGAGAAGTCCATGTC
PR0782	ATTACGGCCGCTAGCGCTACCGGACTCAGATCCACCGGTCGCCACCATGCCCCGCCC CAAGCTCAAGTCCGATG
PR0783	TTTTCCTCCACGTCCCCGCATGTTAGAAGACTTCCCCTGCCCTCGGCTCTGGTACCCC CACCGTACTCGTCAATTCCAAGG
PR0784	GCAGGGGAAGTCTTCTAACATGCGGGGACGTGGAGGAAAATCCCGGGCCCAGATCTG TGAGCAAGGGCGAGGAGCTGTTCAC
PR0789	TTGCACTAGTCGCGTGACTCGAGTTCGTGTTGGCGGCCGCAAGCTTCCATGTACTTGG CTACTTGTACAGCTCGTCCATGCCGAGAGTGATC
PR0793	ATTTTAACAAAATATTAACGCTTACAATTTACGCCTTAAGATAC
PR0825	AAGCAGAGCTGGTTTAGTGAACCGTCAGAT
PR0826	GCGGCCGCAGCTTATTGTTCATTTTTGAGAACTCGCTCAACGAACGATTT
PR0827	CGCGGTCTCCAATTGCCACCATGGAAGACGCCAAAAAC
PR0828	TTGTGGAATCGCCGCTTTCG
PR0830	GATCCACCGGTCGCCAC
PR0876	GGGCATGGCTTCCCGCCGGCGGTGGCGGCGCAGGATGATGGCACGCTGCCCATGTC TTGTGCCCAGGAGAGCGGGATGGACCGTCAC
PR0877	ATCGTGACGGTCCATCCCGCTCTCCTGGGCACAAGACATGGGCAGCGTGCCATCATC CTGCGCCGCCACCGCCGGCGGGAAGCCATG
PR0913	GGTTCCAGAGATCTGGGCATGGCTTCCCGCCGGC
PR0914	GGTTCCAGGAATTCCTCGAGCTACACATTGATCCTAGCAGAAGCACAGGCTGCAGGGT GACGGTCCATCCCGCTCTCCTGG
PR0932	GAATGGACTAGAGCTCGCCACC ATGCAGATCTTCGTGAAAACCCTTACC
PR0933	GAATGGACTATCTAGACACACCTCTCAGACGCAGGAC
PR0934	GAATGGACTACCTAGGATGCAGATCTTCGTGAAAACCCTTACC

PR0935	GAATGGACTAGAATTCCACACCTCTCAGACGCAGGAC
PR0936	GAATGGACTACAATTGATGCAGATCTTCGTGAAAACCCTTACC
PR0937	GAATGGACTACTGCAGCACACCTCTCAGACGCAGGAC
PR0938	GAATGGACTAATGCATATGCAGATCTTCGTGAAAACCCTTACC
PR0939	GAATGGACTAGGATCCCACACCTCTCAGACGCAGGAC
PR0940	GAATGGACTACCACGTACCTGGGCTCGCCACCATGCAGATCTTC
PR0941	GAATGGACTACCAAGTACATGGACCGGTGGATCCCACACCTCTC
PR0995	GAATGGACTATGTACAAGCATGGCTTCCCGCCGGCGGTG
PR0996	GAATGGACTAAAGCTTAAGATCAACGTCTCGTCGAGAATTCGCGGCCGCCGAGCTACA CATTGATCCTAGCAGAAGCACAGGCTGCAGGGTGAC
PR0947	TCGAGGAGCATTCCTGGCTCGATGAGTTAGAAGATTTCTCGAGAAATCTTCTAACTCAT CGAGCGGAGTGTTTG
PR0948	AATTCAAACACTCCGCTCGATGAGTTAGAAGATTTCTCGAGAAATCTTCTAACTCATCG AGCCAGGAATGCTCC
PR0992	CAGCAACTGGTCCTTGGTGTCGACGTACCAGTACACGGACATC
PR0993	GAGGCCGCCACCGTAGTGCTGAAG
PR0994	CAGTACCTCGTCATCGGACTTGAG
PR1069	GGCCGCAAACTACCTGCACTATAAGCACTTTACTACCTGCACTATAAGCACTTTACTAC CTGCACTATAAGCACTTTACTACCTGCACTATAAGCACTTTACGTCGACGCACCTGC
PR1070	GCATGCAGGTGCGTCGACGTAAAGTGCTTATAGTGCAGGTAGTAAAGTGCTTATAGTG CAGGTAGTAAAGTGCTTATAGTGCAGGTAGTAAAGTGCTTATAGTGCAGGTAGTTTGC
PR1071	ATGCGGCCAAAATGCCCTTTTAACATTGCACTGATGCCCTTTTAACATTGCACTGATGC CCTTTTAACATTGCACTGATGCCCTTTTAACATTGCACTGTCGAATGCGCAGGTGA
PR1072	AGCTTCACCTGCGCATTCGACAGTGCAATGTTAAAAGGGCATCAGTGCAATGTTAAAA GGGCATCAGTGCAATGTTAAAAGGGCATCAGTGCAATGTTAAAAGGGCATTTTGGCC
PR1093	TTCCTCTAATGGTCGACCCCTGAGGAAAAAAAAGGAAACAATTGAAAAAAGTGATTTAA TTTATACCATTTTAATTCAGCTTTGTAAA
PR1094	GAATGGACTAGAATTCGCTCGCCACCATGCAGATCTTC
PR1125	GAATGGACTAACCGGTCCGGTGGATCCCACACCTCTC
PR1277	GGCCGCAAACTACCTGCACTATAAGCACTTTAA
PR1278	AGCTTTAAAGTGCTTATAGTGCAGGTAGTTTGC
PR1279	GGCCGCAAACTACCTGCACTATAAGCACTTTACTACCTGCACTATAAGCACTTTAA
PR1280	AGCTTTAAAGTGCTTATAGTGCAGGTAGTAAAGTGCTTATAGTGCAGGTAGTTTGC
PR1307	CAGAATTGTACATAACCCACCGTACTCGTCAATTCCAAG

PR1308	GATATTGCCACCACCGGTATGAGTCGAGGAGAGGTGCGC
PR1320	CGCGTAAACCATCTTTACCAGACAGTGTTACCATCTTTACCAGACAGTGTTACCATCTT TACCAGACAGTGTTACCATCTTTACCAGACAGTGTTA
PR1321	TCGATAACACTGTCTGGTAAAGATGGTAACACTGTCTGGTAAAGATGGTAACACTGTCT GGTAAAGATGGTAACACTGTCTGGTAAAGATGGTTTA
PR1441	GAATGGACTAGGGCCCAAGTAGCCAAGTACATGGAAG
PR1442	GAATGGACTAGGGCCCGCCGCATCGATAAGCTTAAC
PR1443	GAATGGACTATCTAGAATGGCTGATTATCGTCTC
PR1451	GAATGGACTAGGTCTCTGTACATCAGGCCAGGGCGCT
PR1492	GAATGGACTAGGTCTCCGGCCGCATGGCTGATTATCGTCTC
PR1493	GGCCGCCCGCTTGAAGTCTTTAATTAAACCGCTTGAAGTCTTTAATTAAACCGCTTGAA GTCTTTAATTAAACCGCTTGAAGTCTTTAATTAAAG
PR1494	TCGACTTTAATTAAAGACTTCAAGCGGTTTAATTAAAGACTTCAAGCGGTTTAATTAAAG ACTTCAAGCGGTTTAATTAAAGACTTCAAGCGGGC

Supplementary Table 4 List of gBlocks used

gBlock0013	GCGGCCGCCAACACGAACTCGAGTCACGCGACTAGTGCAACGAGCTCTCGAGGT CATCACGCGTTCCGTGATCTCGAGGAATCGGGCGCGCCGGCCAAGATCGAAGA GACGATAATCAGCCATACCACATTTGTAGAGGTTTTACTTGCTTTAAAAAACCTCC CACACCTCCCCCTGAACCTGAAACATAAAATGAATGCAATTGTTGTTGTTAACTTG TTTATTGCAGCTTATAATGGTTACAAATAAAGCAATAGCATCACAAATTTCACAAAT AAAGCATTTTTTTCACTGCCCCGAGCTTCCTCGCTCGTCCAAACTCATCAATGTAT CTTAAGGCGTAAATTGTAAGCGTTAATATTTTGTTAAAAT
gBlock0014	ATGCAGATCTTCGTGAAAACCCTTACCGGCAAGACCATCACCCTTGAGGTGGAGC CCAGTGACACCATCGAAAATGTGAAGGCCAAGATCCAGGATAAGGAAGGCATTC CTCCCGACCAGCAGAGGCTCATCTTTGCAGGCAAGCAGCTGGAAGATGGCCGTA CTCTTTCTGACTACAACATCCAGAAGGAGTCGACCCTGCACCTGGTCCTGCGTCT GAGAGGTGTG

Supplementary Table 5 Transfection setup of experiment shown in **Figure 2a**. The numbers are the nanogram (ng) plasmid amounts co-transfected per sample in a 96-well setup.

	200c	145	141	375	146a	196a	24	142	let-7b	125a	18a	7	30a
AmCyan-TRE-DsRed-T200cx4 (pBH0019)	25												
mCerulean-TRE-mCherry-T145x4 (pBH0193)		25											
mCerulean-TRE-mCherry-141x4 (pBH0267)			25										
mCerulean-TRE-mCherry-T375x4 (pBH0194)				25									
mCerulean-TRE-mCherry-T146ax4 (pBH0195)					25								
AmCyan-TRE-DsRed-T196ax4 (pBH0014)						100							
mCerulean-TRE-mCherry-T24x4 (pBH0084)							25						
AmCyan-TRE-DsRed-T142-3px4 (pZ117)								25					
mCerulean-TRE-mCherry-Tlet7bx4 (pBH0082)									25				
mCerulean-TRE-mCherry-T125ax4 (pBH0077)										25			
mCerulean-TRE-mCherry-T18ax4 (pBH0078)											25		
mCerulean-TRE-mCherry-T7x4 (pBH0079)												25	
AmCyan-TRE-DsRed-T30ax4 (pZ146)													25
mCerulean-TRE-mCherry-T27bx4 (pBH0076)													
AmCyan-TRE-DsRed-T23bx4 (pBH0022)													
mCerulean-TRE-mCherry-T16x4 (pBH0083)													
mCerulean-TRE-mCherry-T20ax4 (pBH0080)													
mCerulean-TRE-mCherry-T122x4 (pBH0112)													
mCerulean-TRE-mCherry-T130ax4 (pBH0075)													
AmCyan-TRE-DsRed-T17x4 (pZ145)													
mCerulean-TRE-mCherry-T21x4 (pBH0081)													
Junk-DNA, Ubi-empty-NOS (pDT7004)	137.5	137.5	137.5	137.5	137.5	137.5	137.5	137.5	137.5	137.5	137.5	137.5	137.5
CMV-rtTA-TFF5 (pZ091)	12.5	12.5	12.5	12.5	12.5	12.5	12.5	12.5	12.5	12.5	12.5	12.5	12.5

Supplementary Table 5 Continuation

	27b	23b	16	20a	122	130a	17	21
AmCyan-TRE-DsRed-T200cx4 (pBH0019)								
mCerulean-TRE-mCherry-T145x4 (pBH0193)								
mCerulean-TRE-mCherry-141x4 (pBH0267)								
mCerulean-TRE-mCherry-T375x4 (pBH0194)								
mCerulean-TRE-mCherry-T146ax4 (pBH0195)								
AmCyan-TRE-DsRed-T196ax4 (pBH0014)								
mCerulean-TRE-mCherry-T24x4 (pBH0084)								
AmCyan-TRE-DsRed-T142-3px4 (pZ117)								
mCerulean-TRE-mCherry-Tlet7bx4 (pBH0082)								
mCerulean-TRE-mCherry-T125ax4 (pBH0077)								
mCerulean-TRE-mCherry-T18ax4 (pBH0078)								
mCerulean-TRE-mCherry-T7x4 (pBH0079)								
AmCyan-TRE-DsRed-T30ax4 (pZ146)								
mCerulean-TRE-mCherry-T27bx4 (pBH0076)	25							
AmCyan-TRE-DsRed-T23bx4 (pBH0022)		25						
mCerulean-TRE-mCherry-T16x4 (pBH0083)			25					
mCerulean-TRE-mCherry-T20ax4 (pBH0080)				25				
mCerulean-TRE-mCherry-T122x4 (pBH0112)					25			
mCerulean-TRE-mCherry-T130ax4 (pBH0075)						25		
AmCyan-TRE-DsRed-T17x4 (pZ145)							25	
mCerulean-TRE-mCherry-T21x4 (pBH0081)								25
Junk-DNA, Ubi-empty-NOS (pDT7004)	137.5	137.5	137.5	137.5	137.5	137.5	137.5	137.5
CMV-rtTA-TFF5 (pZ091)	12.5	12.5	12.5	12.5	12.5	12.5	12.5	12.5

Supplementary Table 6 Transfection setup of experiment shown in **Figure 2c, d.** The numbers are the nanogram (ng) plasmid amounts co-transfected per sample in a 24-well setup. In case of LNAs/ mimics, the amounts are reported in nM final concentration.

	LNA-Neg.Ctrl.	LNA-21	LNA-20a	Mim-Neg.Ctrl.	Mim-146a	Mim-141
CMV-rtTA-T21x4 (pZ090)	12.5	12.5	12.5	12.5	12.5	12.5
TRE-LacI-T21x4-miR-FF4 (pZ224)	12.5	12.5	12.5	12.5	12.5	12.5
CMV-rtTA-T20ax4 (pBH0211)	12.5	12.5	12.5	12.5	12.5	12.5
TRE-LacI-T20ax4-miR-FF4 (pBH0212)	12.5	12.5	12.5	12.5	12.5	12.5
CAGop-Citrine-2A-PIT2-T146ax4-T141x4-TFF4x4 (pBH0254)	25	25	25	25	25	25
mCerulean-PRE-mCherry-T122x4 (pBH0182)	100	100	100	100	100	100
CMV-iRFP (pCS0012)	100	100	100	100	100	100
Lac-op free Junk-DNA Ubi-Nos (pBH0265)	425	425	425	425	425	425
LNA-Neg.Ctrl.	5					
LNA-21		5				
LNA-20a			5			
Mim-Neg.Ctrl.				5		
Mim-146a					5	
Mim-141						5

Supplementary Table 7 Transfection setup of experiment shown in **Figure 3e**. The numbers are the nanogram (ng) plasmid amounts co-transfected per sample in a 24-well setup. In case of LNAs/ mimics, the amounts are reported in nM final concentration.

	Parallel assay																	
CMV-rtTA-T21x4 (pZ090)	12.5	12.5	12.5	12.5	12.5	12.5	12.5	12.5	12.5	12.5	12.5	12.5	12.5	12.5	12.5	12.5	12.5	12.5
TRE-LacI-T21x4-miR-FF4 (pZ224)	12.5	12.5	12.5	12.5	12.5	12.5	12.5	12.5	12.5	12.5	12.5	12.5	12.5	12.5	12.5	12.5	12.5	12.5
CMV-rtTA-T20ax4 (pBH0211)	12.5	12.5	12.5	12.5	12.5	12.5	12.5	12.5	12.5	12.5	12.5	12.5	12.5	12.5	12.5	12.5	12.5	12.5
TRE-LacI-T20ax4-miR-FF4 (pBH0212)	12.5	12.5	12.5	12.5	12.5	12.5	12.5	12.5	12.5	12.5	12.5	12.5	12.5	12.5	12.5	12.5	12.5	12.5
CAGop-ZsYellow-T146ax4-T141x4-TFF4x4 (pBH0260)	25	25	25	25	25	25	25	25	25	25	25	25	25	25	25	25	25	25
CMV-PIT2 (pMF206)	50	50	50	50	50	50	50	50	50	50	50	50	50	50	50	50	50	50
mCerulean-PRE-mCherry-T122x4 (pBH0182)	100	100	100	100	100	100	100	100	100	100	100	100	100	100	100	100	100	100
CMV-IFP1.4 (pZ210)	100	100	100	100	100	100	100	100	100	100	100	100	100	100	100	100	100	100
Lac-op free Junk-DNA Ubi-Nos (pBH0265)	375	375	375	375	375	375	375	375	375	375	375	375	375	375	375	375	375	375
LNA-Neg.Ctrl.		5																
LNA-122			5				5	5										
LNA-21				5			5	5										
LNA-20a					5		5	5										
LNA-FF4						5		5										
Mim-Neg.Ctrl.									5									
Mim-122										5						5	5	5
Mim-21											5						5	5
Mim-20a												5					5	5
siFF4													5					5
Mim-141														5		5	5	5
Mim-146a															5	5	5	5

Supplementary Table 7 Continuation

	LFF assay																	
CMV-rtTA-T21x4 (pZ090)	12.5	12.5	12.5	12.5	12.5	12.5	12.5	12.5	12.5	12.5	12.5	12.5	12.5	12.5	12.5	12.5	12.5	12.5
TRE-LacI-T21x4-miR-FF4 (pZ224)	12.5	12.5	12.5	12.5	12.5	12.5	12.5	12.5	12.5	12.5	12.5	12.5	12.5	12.5	12.5	12.5	12.5	12.5
CMV-rtTA-T20ax4 (pBH0211)	12.5	12.5	12.5	12.5	12.5	12.5	12.5	12.5	12.5	12.5	12.5	12.5	12.5	12.5	12.5	12.5	12.5	12.5
TRE-LacI-T20ax4-miR-FF4 (pBH0212)	12.5	12.5	12.5	12.5	12.5	12.5	12.5	12.5	12.5	12.5	12.5	12.5	12.5	12.5	12.5	12.5	12.5	12.5
CAGop-PIT2-T146ax4-T141x4-TFF4x4 (pBH0256)	25	25	25	25	25	25	25	25	25	25	25	25	25	25	25	25	25	25
T141x4-T146ax4-mCerulean-PRE-mCherry-T122x4 (pBH0246)	100	100	100	100	100	100	100	100	100	100	100	100	100	100	100	100	100	100
Ef1α-mCitrine (pKH025)	100	100	100	100	100	100	100	100	100	100	100	100	100	100	100	100	100	100
Lac-op free Junk-DNA Ubi-Nos (pBH0265)	425	425	425	425	425	425	425	425	425	425	425	425	425	425	425	425	425	425
LNA-Neg.Ctrl.		5																
LNA-122			5				5	5										
LNA-21				5			5	5										
LNA-20a					5		5	5										
LNA-FF4						5		5										
Mim-Neg.Ctrl.									5									
Mim-122										5						5	5	5
Mim-21											5						5	5
Mim-20a												5					5	5
siFF4													5					5
Mim-141														5		5	5	5
Mim-146a															5	5	5	5

Supplementary Table 7 Continuation

	CFF assay																	
CMV-rtTA-T21x4 (pZ090)	12.5	12.5	12.5	12.5	12.5	12.5	12.5	12.5	12.5	12.5	12.5	12.5	12.5	12.5	12.5	12.5	12.5	12.5
TRE-LacI-T21x4-miR-FF4 (pZ224)	12.5	12.5	12.5	12.5	12.5	12.5	12.5	12.5	12.5	12.5	12.5	12.5	12.5	12.5	12.5	12.5	12.5	12.5
CMV-rtTA-T20ax4 (pBH0211)	12.5	12.5	12.5	12.5	12.5	12.5	12.5	12.5	12.5	12.5	12.5	12.5	12.5	12.5	12.5	12.5	12.5	12.5
TRE-LacI-T20ax4-miR-FF4 (pBH0212)	12.5	12.5	12.5	12.5	12.5	12.5	12.5	12.5	12.5	12.5	12.5	12.5	12.5	12.5	12.5	12.5	12.5	12.5
CAGop-PIT2-T146ax4-T141x4-TFF4x4 (pBH0256)	25	25	25	25	25	25	25	25	25	25	25	25	25	25	25	25	25	25
TFF4x4-T141x4-T146ax4-mCerulean-PRE-mCherry-T122x4 (pBH0264)	100	100	100	100	100	100	100	100	100	100	100	100	100	100	100	100	100	100
Ef1α-mCitrine (pKH025)	100	100	100	100	100	100	100	100	100	100	100	100	100	100	100	100	100	100
Lac-op free Junk-DNA Ubi-Nos (pBH0265)	425	425	425	425	425	425	425	425	425	425	425	425	425	425	425	425	425	425
LNA-Neg.Ctrl.		5																
LNA-122			5				5	5										
LNA-21				5			5	5										
LNA-20a					5		5	5										
LNA-FF4						5		5										
Mim-Neg.Ctrl.									5									
Mim-122										5						5	5	5
Mim-21											5						5	5
Mim-20a												5					5	5
siFF4													5					5
Mim-141														5		5	5	5
Mim-146a															5	5	5	5

Supplementary Table 8 Transfection setup of experiment shown in **Figure 4b** and **Supplementary Figure 7a, b**. The numbers are the nanogram (ng) plasmid amounts co-transfected per sample in a 24-well setup. In case of LNAs/ mimics, the amounts are reported in nM final concentration.

	CFF assay																	
CMV-rtTA-T21x4 (pZ090)	12.5	12.5	12.5	12.5	12.5	12.5	12.5	12.5	12.5	12.5	12.5	12.5	12.5	12.5	12.5	12.5	12.5	12.5
TRE-LacI-T21x4-miR-FF4 (pZ224)	12.5	12.5	12.5	12.5	12.5	12.5	12.5	12.5	12.5	12.5	12.5	12.5	12.5	12.5	12.5	12.5	12.5	12.5
CMV-rtTA-T20ax4 (pBH0211)	12.5	12.5	12.5	12.5	12.5	12.5	12.5	12.5	12.5	12.5	12.5	12.5	12.5	12.5	12.5	12.5	12.5	12.5
TRE-LacI-T20ax4-miR-FF4 (pBH0212)	12.5	12.5	12.5	12.5	12.5	12.5	12.5	12.5	12.5	12.5	12.5	12.5	12.5	12.5	12.5	12.5	12.5	12.5
CAGop-PIT2-T146ax4-T141x4-TFF4x4 (pBH0256)	25	25	25	25	25	25	25	25	25	25	25	25	25	25	25	25	25	25
TFF4x4-T141x4-T146ax4-mCerulean-PRE-mCherry-T122x4 (pBH0264)	100	100	100	100	100	100	100	100	100	100	100	100	100	100	100	100	100	100
Ef1α-mCitrine (pKH025)	100	100	100	100	100	100	100	100	100	100	100	100	100	100	100	100	100	100
Lac-op free Junk-DNA Ubi-Nos (pBH0265)	425	425	425	425	425	425	425	425	425	425	425	425	425	425	425	425	425	425
LNA-Neg.Ctrl.		5																
LNA-122			5				5	5										
LNA-21				5			5	5										
LNA-20a					5		5	5										
LNA-FF4						5		5										
Mim-Neg.Ctrl.									5									
Mim-122										5						5	5	5
Mim-21											5						5	5
Mim-20a												5					5	5
siFF4													5					5
Mim-141														5		5	5	5
Mim-146a															5	5	5	5

Supplementary Table 9 Transfection setup of experiment shown in **Figure 5**. The numbers are the nanogram (ng) plasmid amounts co-transfected per sample in a 24-well setup. In case of LNAs/ mimics, the amounts are reported in nM final concentration.

	high mimic/LNA																	
CMV-rtTA-T21x4 (pZ090)	12.5	12.5	12.5	12.5	12.5	12.5	12.5	12.5	12.5	12.5	12.5	12.5	12.5	12.5	12.5	12.5	12.5	12.5
TRE-LacI-T21x4-miR-FF4 (pZ224)	12.5	12.5	12.5	12.5	12.5	12.5	12.5	12.5	12.5	12.5	12.5	12.5	12.5	12.5	12.5	12.5	12.5	12.5
CMV-rtTA-T20ax4 (pBH0211)	12.5	12.5	12.5	12.5	12.5	12.5	12.5	12.5	12.5	12.5	12.5	12.5	12.5	12.5	12.5	12.5	12.5	12.5
TRE-LacI-T20ax4-miR-FF4 (pBH0212)	12.5	12.5	12.5	12.5	12.5	12.5	12.5	12.5	12.5	12.5	12.5	12.5	12.5	12.5	12.5	12.5	12.5	12.5
CAGop-PIT2-T146ax4-T141x4-TFF4x4 (pBH0256)	25	25	25	25	25	25	25	25	25	25	25	25	25	25	25	25	25	25
TFF4x4-T141x4-T146ax4-mCerulean-PRE-mCherry-T122x4 (pBH0264)	100	100	100	100	100	100	100	100	100	100	100	100	100	100	100	100	100	100
Ef1α-mCitrine (pKH025)	100	100	100	100	100	100	100	100	100	100	100	100	100	100	100	100	100	100
Lac-op free Junk-DNA Ubi-Nos (pBH0265)	425	425	425	425	425	425	425	425	425	425	425	425	425	425	425	425	425	425
LNA-Neg.Ctrl.		5																
LNA-122			5				5	5										
LNA-21				5			5	5										
LNA-20a					5		5	5										
LNA-FF4						5		5										
Mim-Neg.Ctrl.									5									
Mim-122										5						5	5	5
Mim-21											5						5	5
Mim-20a												5					5	5
siFF4													5					5
Mim-141														5		5	5	5
Mim-146a															5	5	5	5

Supplementary Table 9 Continuation

	medium mimic/LNA																
CMV-rtTA-T21x4 (pZ090)	12.5	12.5	12.5	12.5	12.5	12.5	12.5	12.5	12.5	12.5	12.5	12.5	12.5	12.5	12.5	12.5	12.5
TRE-LacI-T21x4-miR-FF4 (pZ224)	12.5	12.5	12.5	12.5	12.5	12.5	12.5	12.5	12.5	12.5	12.5	12.5	12.5	12.5	12.5	12.5	12.5
CMV-rtTA-T20ax4 (pBH0211)	12.5	12.5	12.5	12.5	12.5	12.5	12.5	12.5	12.5	12.5	12.5	12.5	12.5	12.5	12.5	12.5	12.5
TRE-LacI-T20ax4-miR-FF4 (pBH0212)	12.5	12.5	12.5	12.5	12.5	12.5	12.5	12.5	12.5	12.5	12.5	12.5	12.5	12.5	12.5	12.5	12.5
CAGop-PIT2-T146ax4-T141x4-TFF4x4 (pBH0256)	25	25	25	25	25	25	25	25	25	25	25	25	25	25	25	25	25
TFF4x4-T141x4-T146ax4-mCerulean-PRE-mCherry-T122x4 (pBH0264)	100	100	100	100	100	100	100	100	100	100	100	100	100	100	100	100	100
Ef1α-mCitrine (pKH025)	100	100	100	100	100	100	100	100	100	100	100	100	100	100	100	100	100
Lac-op free Junk-DNA Ubi-Nos (pBH0265)	425	425	425	425	425	425	425	425	425	425	425	425	425	425	425	425	425
LNA-Neg.Ctrl.	1																
LNA-122		1				1	1										
LNA-21			1			1	1										
LNA-20a				1		1	1										
LNA-FF4					1		1										
Mim-Neg.Ctrl.								1									
Mim-122									1						1	1	1
Mim-21										1						1	1
Mim-20a											1					1	1
siFF4												1					1
Mim-141													1		1	1	1
Mim-146a														1	1	1	1

Supplementary Table 9 Continuation

	low mimic/LNA																
CMV-rtTA-T21x4 (pZ090)	12.5	12.5	12.5	12.5	12.5	12.5	12.5	12.5	12.5	12.5	12.5	12.5	12.5	12.5	12.5	12.5	12.5
TRE-LacI-T21x4-miR-FF4 (pZ224)	12.5	12.5	12.5	12.5	12.5	12.5	12.5	12.5	12.5	12.5	12.5	12.5	12.5	12.5	12.5	12.5	12.5
CMV-rtTA-T20ax4 (pBH0211)	12.5	12.5	12.5	12.5	12.5	12.5	12.5	12.5	12.5	12.5	12.5	12.5	12.5	12.5	12.5	12.5	12.5
TRE-LacI-T20ax4-miR-FF4 (pBH0212)	12.5	12.5	12.5	12.5	12.5	12.5	12.5	12.5	12.5	12.5	12.5	12.5	12.5	12.5	12.5	12.5	12.5
CAGop-PIT2-T146ax4-T141x4-TFF4x4 (pBH0256)	25	25	25	25	25	25	25	25	25	25	25	25	25	25	25	25	25
TFF4x4-T141x4-T146ax4-mCerulean-PRE-mCherry-T122x4 (pBH0264)	100	100	100	100	100	100	100	100	100	100	100	100	100	100	100	100	100
Ef1α-mCitrine (pKH025)	100	100	100	100	100	100	100	100	100	100	100	100	100	100	100	100	100
Lac-op free Junk-DNA Ubi-Nos (pBH0265)	425	425	425	425	425	425	425	425	425	425	425	425	425	425	425	425	425
LNA-Neg.Ctrl.	0.1																
LNA-122		0.1				0.1	0.1										
LNA-21			0.1			0.1	0.1										
LNA-20a				0.1		0.1	0.1										
LNA-FF4					0.1		0.1										
Mim-Neg.Ctrl.								0.1									
Mim-122									0.1						0.1	0.1	0.1
Mim-21										0.1						0.1	0.1
Mim-20a											0.1					0.1	0.1
siFF4												0.1					0.1
Mim-141													0.1		0.1	0.1	0.1
Mim-146a														0.1	0.1	0.1	0.1

Supplementary Table 10 Transfection setup of the experiment shown in **Figure 6b** and **Supplementary Figures 8, 9**. The numbers are the nanogram (ng) plasmid amounts co-transfected per sample in a 96-well setup. In case of LNAs/ mimics, the amounts are reported in nM final concentration, for compounds in µM.

	DMSO/ Samples	LNA-122	Mim-122	LNA-21	Mim-146a
CMV-rtTA-T21x4 (pZ090)	3.125	3.125	3.125	3.125	3.125
TRE-LacI-T21x4-miR-FF4 (pZ224)	3.125	3.125	3.125	3.125	3.125
CMV-rtTA-T20ax4 (pBH0211)	3.125	3.125	3.125	3.125	3.125
TRE-LacI-T20ax4-miR-FF4 (pBH0212)	3.125	3.125	3.125	3.125	3.125
CAGop-PIT2-T146ax4-T141x4-TFF4x4 (pBH0256)	6.25	6.25	6.25	6.25	6.25
TFF4x4-T141x4-T146ax4-mCerulean-PRE-mCherry-T122x4 (pBH0264)	25	25	25	25	25
Ef1α-mCitrine (pKH025)	25	25	25	25	25
Lac-op free Junk-DNA Ubi-Nos (pBH0265)	106.25	106.25	106.25	106.25	106.25
DMSO/Compound	1%/10				
LNA-122		5			
Mim-122			5		
LNA-21				5	
Mim-146a					5

Supplementary Table 11 Transfection setup of the experiment shown in **Figure 6c**. The numbers are the nanogram (ng) plasmid amounts co-transfected per sample in a 96-well setup. In case of LNAs/ mimics, the amounts are reported in nM final concentration, for compounds in µM.

	DMSO	Tadalafil				Indinavir				Donepezil			
CMV-rtTA-T21x4 (pZ090)	3.125	3.125				3.125				3.125			
TRE-LacI-T21x4-miR-FF4 (pZ224)	3.125	3.125				3.125				3.125			
CMV-rtTA-T20ax4 (pBH0211)	3.125	3.125				3.125				3.125			
TRE-LacI-T20ax4-miR-FF4 (pBH0212)	3.125	3.125				3.125				3.125			
CAGop-PIT2-T146ax4-T141x4-TFF4x4 (pBH0256)	6.25	6.25				6.25				6.25			
TFF4x4-T141x4-T146ax4-mCerulean-PRE-mCherry-T122x4 (pBH0264)	100	100				100				100			
Ef1α-mCitrine (pKH025)	25	25				25				25			
Lac-op free Junk-DNA Ubi-Nos (pBH0265)	34.375	34.375				34.375				34.375			
Compound	1%	0.4				0.4				0.4			
Compound			2				2				2		
Compound				10				10				10	
Compound					50				50				50
Compound													
		Indatraline				Hexachlorophene				Chloroxine			
CMV-rtTA-T21x4 (pZ090)		3.125				3.125				3.125			
TRE-LacI-T21x4-miR-FF4 (pZ224)		3.125				3.125				3.125			
CMV-rtTA-T20ax4 (pBH0211)		3.125				3.125				3.125			
TRE-LacI-T20ax4-miR-FF4 (pBH0212)		3.125				3.125				3.125			
CAGop-PIT2-T146ax4-T141x4-TFF4x4 (pBH0256)		6.25				6.25				6.25			
TFF4x4-T141x4-T146ax4-mCerulean-PRE-mCherry-T122x4 (pBH0264)		100				100				100			
Ef1α-mCitrine (pKH025)		25				25				25			
Lac-op free Junk-DNA Ubi-Nos (pBH0265)		34.375				34.375				34.375			
Compound		0.4				0.4				0.4			
Compound			2				2				2		
Compound				10				10				10	
Compound					50				50				50

Supplementary Table 11 Continuation.

	Levothyroxine				Lomerizine			
CMV-rtTA-T21x4 (pZ090)	3.125				3.125			
TRE-LacI-T21x4-miR-FF4 (pZ224)	3.125				3.125			
CMV-rtTA-T20ax4 (pBH0211)	3.125				3.125			
TRE-LacI-T20ax4-miR-FF4 (pBH0212)	3.125				3.125			
CAGop-PIT2-T146ax4-T141x4-TFF4x4 (pBH0256)	6.25				6.25			
TFF4x4-T141x4-T146ax4-mCerulean-PRE-mCherry-T122x4 (pBH0264)	100				100			
Ef1α-mCitrine (pKH025)	25				25			
Lac-op free Junk-DNA Ubi-Nos (pBH0265)	34.375				34.375			
Compound	0.4				0.4			
Compound		2				2		
Compound			10				10	
Compound				50				50
Compound								
	Digoxin				Mefloquine			
CMV-rtTA-T21x4 (pZ090)	3.125				3.125			
TRE-LacI-T21x4-miR-FF4 (pZ224)	3.125				3.125			
CMV-rtTA-T20ax4 (pBH0211)	3.125				3.125			
TRE-LacI-T20ax4-miR-FF4 (pBH0212)	3.125				3.125			
CAGop-PIT2-T146ax4-T141x4-TFF4x4 (pBH0256)	6.25				6.25			
TFF4x4-T141x4-T146ax4-mCerulean-PRE-mCherry-T122x4 (pBH0264)	100				100			
Ef1α-mCitrine (pKH025)	25				25			
Lac-op free Junk-DNA Ubi-Nos (pBH0265)	34.375				34.375			
Compound	0.4				0.4			
Compound		2				2		
Compound			10				10	
Compound				50				50

Supplementary Table 12 Transfection setup of the experiment shown in **Figure 6d**. The numbers are the nanogram (ng) plasmid amounts co-transfected per sample in a 96-well setup. In case of LNAs/ mimics, the amounts are reported in nM final concentration, for compounds in µM.

	DMSO	Ritonavir				Saquinavir				Ifenprodil			
CMV-rtTA-T21x4 (pZ090)	3.125	3.125				3.125				3.125			
TRE-LacI-T21x4-miR-FF4 (pZ224)	3.125	3.125				3.125				3.125			
CMV-rtTA-T20ax4 (pBH0211)	3.125	3.125				3.125				3.125			
TRE-LacI-T20ax4-miR-FF4 (pBH0212)	3.125	3.125				3.125				3.125			
CAGop-PIT2-T146ax4-T141x4-TFF4x4 (pBH0256)	6.25	6.25				6.25				6.25			
TFF4x4-T141x4-T146ax4-mCerulean-PRE-mCherry-T122x4 (pBH0264)	100	100				100				100			
Ef1α-mCitrine (pKH025)	25	25				25				25			
Lac-op free Junk-DNA Ubi-Nos (pBH0265)	34.375	34.375				34.375				34.375			
Compound	1%	0.4				0.4				0.4			
Compound			2				2				2		
Compound				10				10				10	
Compound					50				50				50

**Supplementary Table 12** Continuation	Amlodipine				Oxytetracycline				Clobetasol propionate				Rifabutin				Rifapentine			
CMV-rtTA-T21x4 (pZ090)	3.125				3.125				3.125				3.125				3.125			
TRE-LacI-T21x4-miR-FF4 (pZ224)	3.125				3.125				3.125				3.125				3.125			
CMV-rtTA-T20ax4 (pBH0211)	3.125				3.125				3.125				3.125				3.125			
TRE-LacI-T20ax4-miR-FF4 (pBH0212)	3.125				3.125				3.125				3.125				3.125			
CAGop-PIT2-T146ax4-T141x4-TFF4x4 (pBH0256)	6.25				6.25				6.25				6.25				6.25			
TFF4x4-T141x4-T146ax4-mCerulean-PRE-mCherry-T122x4 (pBH0264)	100				100				100				100				100			
Ef1α-mCitrine (pKH025)	25				25				25				25				25			
Lac-op free Junk-DNA Ubi-Nos (pBH0265)	34.375				34.375				34.375				34.375				34.375			
Compound	0.4				0.4				0.4				0.4				0.4			
Compound		2				2				2				2				2		
Compound			10				10				10				10				10	
Compound				50				50				50				50				50

Supplementary Table 13 Transfection setup of the experiment shown in **Figure 7b**. The numbers are the nanogram (ng) plasmid amounts co-transfected per sample in a 96-well setup. In case of LNAs/ mimics, the amounts are reported in nM final concentration, for compounds in µM.

	miR-23b assay																
CMV-rtTA-T21x4 (pZ090)	3.125	3.125	3.125	3.125	3.125	3.125	3.125	3.125	3.125	3.125	3.125	3.125	3.125	3.125	3.125	3.125	3.125
TRE-LacI-T21x4-miR-FF4 (pZ224)	3.125	3.125	3.125	3.125	3.125	3.125	3.125	3.125	3.125	3.125	3.125	3.125	3.125	3.125	3.125	3.125	3.125
CMV-rtTA-T20ax4 (pBH0211)	3.125	3.125	3.125	3.125	3.125	3.125	3.125	3.125	3.125	3.125	3.125	3.125	3.125	3.125	3.125	3.125	3.125
TRE-LacI-T20ax4-miR-FF4 (pBH0212)	3.125	3.125	3.125	3.125	3.125	3.125	3.125	3.125	3.125	3.125	3.125	3.125	3.125	3.125	3.125	3.125	3.125
CAGop-PIT2-T146ax4-T141x4TFF4x4 (pBH0256)	6.25	6.25	6.25	6.25	6.25	6.25	6.25	6.25	6.25	6.25	6.25	6.25	6.25	6.25	6.25	6.25	6.25
TFF4x4-T141x4-T146ax4-mCerulean-PRE-mCherry-T23bx4 (pBH0278)	100	100	100	100	100	100	100	100	100	100	100	100	100	100	100	100	100
Ef1α-mCitrine (pKH025)	25	25	25	25	25	25	25	25	25	25	25	25	25	25	25	25	25
Lac-op free Junk-DNA Ubi-Nos (pBH0265)	43.75	43.75	43.75	43.75	43.75	43.75	43.75	43.75	43.75	43.75	43.75	43.75	43.75	43.75	43.75	43.75	43.75
LNA-Neg.Ctrl.	5																
LNA-23b		5				5	5										
LNA-21			5			5	5										
LNA-20a				5		5	5										
LNA-FF4					5		5										
Mim-Neg.Ctrl.								5									
Mim-23b									5						5	5	5
Mim-21										5						5	5
Mim-20a											5					5	5
siFF4												5					5
Mim-146a													5		5	5	5
Mim-141														5	5	5	5

Supplementary Table 14 Transfection setup of the experiment shown in **Figure 7c**. The numbers are the nanogram (ng) plasmid amounts co-transfected per sample in a 96-well setup. In case of LNAs/ mimics, the amounts are reported in nM final concentration, for compounds in µM.

	miR-145/375 assay																	
CMV-rtTA-T21x4 (pZ090)	3.125	3.125	3.125	3.125	3.125	3.125	3.125	3.125	3.125	3.125	3.125	3.125	3.125	3.125	3.125	3.125	3.125	3.125
TRE-LacI-T21x4-miR-FF4 (pZ224)	3.125	3.125	3.125	3.125	3.125	3.125	3.125	3.125	3.125	3.125	3.125	3.125	3.125	3.125	3.125	3.125	3.125	3.125
CMV-rtTA-T20ax4 (pBH0211)	3.125	3.125	3.125	3.125	3.125	3.125	3.125	3.125	3.125	3.125	3.125	3.125	3.125	3.125	3.125	3.125	3.125	3.125
TRE-LacI-T20ax4-miR-FF4 (pBH0212)	3.125	3.125	3.125	3.125	3.125	3.125	3.125	3.125	3.125	3.125	3.125	3.125	3.125	3.125	3.125	3.125	3.125	3.125
CAGop-PIT2-T146ax4-T375x4-T145x4-TFF4x4 (pBH0255)	6.25	6.25	6.25	6.25	6.25	6.25	6.25	6.25	6.25	6.25	6.25	6.25	6.25	6.25	6.25	6.25	6.25	6.25
TFF4x4-T145x4-T375x4-T146ax4-mCerulean-PRE-mCherry-T122x4 (pBH0263)	100	100	100	100	100	100	100	100	100	100	100	100	100	100	100	100	100	100
Ef1α-mCitrine (pKH025)	25	25	25	25	25	25	25	25	25	25	25	25	25	25	25	25	25	25
Lac-op free Junk-DNA Ubi-Nos (pBH0265)	43.75	43.75	43.75	43.75	43.75	43.75	43.75	43.75	43.75	43.75	43.75	43.75	43.75	43.75	43.75	43.75	43.75	43.75
LNA-Neg.Ctrl.	5																	
LNA-122		5				5	5											
LNA-21			5			5	5											
LNA-20a				5		5	5											
LNA-FF4					5		5											
Mim-Neg.Ctrl.								5										
Mim-122									5							5	5	5
Mim-21										5							5	5
Mim-20a											5						5	5
siFF4												5						5
Mim-146a													5			5	5	5
Mim-141																		
Mim-375														5		5	5	5
Mim-145															5	5	5	5

Supplementary Table 15 Transfection setup of the experiment shown in **Figure 7d**. The numbers are the nanogram (ng) plasmid amounts co-transfected per sample in a 96-well setup. In case of LNAs/ mimics, the amounts are reported in nM final concentration.

Circuit assay

	LNA-Neg.Ctrl.	LNA-122/21/20a
CMV-rtTA-T21x4 (pZ090)	3.125	3.125
TRE-LacI-T21x4-miR-FF4 (pZ224)	3.125	3.125
CMV-rtTA-T20ax4 (pBH0211)	3.125	3.125
TRE-LacI-T20ax4-miR-FF4 (pBH0212)	3.125	3.125
CAGop-PIT2-T146ax4-T141x4-TFF4x4 (pBH0256)	6.25	6.25
TFF4x4-T141x4-T146ax4-mCerulean-PRE-mCherry-T122x4 (pBH0264)	100	100
Ef1α-mCitrine (pKH025)	25	25
Lac-op free Junk-DNA Ubi-Nos (pBH0265)	34.375	34.375
LNA-Neg.Ctrl.	3	
LNA-122		1
LNA-21		1
LNA-20a		1

Bidicrectional reporter assay

	LNA-Neg.Ctrl.	LNA-122/21/20a
CMV-rtTA-TFF5x4 (pZ091)	12.5	12.5
mCerulean-PRE-mCherry-T122x4 (pBH0112)	25	100
Lac-op free Junk-DNA Ubi-Nos (pBH0265)	137.5	137.5
LNA-Neg.Ctrl.	3	
LNA-122		1
LNA-21		1
LNA-20a		1

Supplementary Table 16 Transfection setup of the experiment shown in **Figure 7e**. The numbers are the nanogram (ng) plasmid amounts co-transfected per sample in a 96-well setup. In case of LNAs/ mimics, the amounts are reported in nM final concentration.

Circuit assay

	siNeg.Ctrl.	siDrosha/siDicer
CMV-rtTA-T21x4 (pZ090)	3.125	3.125
TRE-LacI-T21x4-miR-FF4 (pZ224)	3.125	3.125
CMV-rtTA-T20ax4 (pBH0211)	3.125	3.125
TRE-LacI-T20ax4-miR-FF4 (pBH0212)	3.125	3.125
CAGop-PIT2-T146ax4-T141x4-TFF4x4 (pBH0256)	6.25	6.25
TFF4x4-T141x4-T146ax4-mCerulean-PRE-mCherry-Tlet7bx4 (pBH0288)	100	100
Ef1α-mCitrine (pKH025)	25	25
Lac-op free Junk-DNA Ubi-Nos (pBH0265)	34.375	34.375
siNeg.Ctrl.	10	
siDrosha		5
siDicer		5

Bidicrectional reporter assay

	siNeg.Ctrl.	siDrosha/siDicer
CMV-rtTA-TFF5x4 (pZ091)	12.5	12.5
mCerulean-PRE-mCherry-Tlet-7bx4 (pBH0082)	25	100
Lac-op free Junk-DNA Ubi-Nos (pBH0265)	137.5	137.5
siNeg.Ctrl.	10	
siDrosha		5
siDicer		5

Supplementary Table 17 Transfection setup of the experiment shown in **Figure 7f**. The numbers are the nanogram (ng) plasmid amounts co-transfected per sample in a 96-well setup. In case of compounds amounts are reported in µM, for DMSO as percentage of total reaction volume.

Circuit assay

	DMSO	Clobetasol
CMV-rtTA-T21x4 (pZ090)	3.125	3.125
TRE-LacI-T21x4-miR-FF4 (pZ224)	3.125	3.125
CMV-rtTA-T20ax4 (pBH0211)	3.125	3.125
TRE-LacI-T20ax4-miR-FF4 (pBH0212)	3.125	3.125
CAGop-PIT2-T146ax4-T141x4-TFF4x4 (pBH0256)	6.25	6.25
TFF4x4-T141x4-T146ax4-mCerulean-PRE-mCherry-T122x4 (pBH0264)	100	100
Ef1α-mCitrine (pKH025)	25	25
Lac-op free Junk-DNA Ubi-Nos (pBH0265)	34.375	34.375
DMSO	1%	
Clobetasol propriate		10

Bidicrectional reporter assay

	DMSO	Clobetasol
CMV-rtTA-TFF5x4 (pZ091)	12.5	12.5
mCerulean-PRE-mCherry-T122x4 (pBH0112)	25	100
Lac-op free Junk-DNA Ubi-Nos (pBH0265)	137.5	137.5
DMSO	1%	
Clobetasol propriate		10

Supplementary Table 18 Transfection setup of experiment shown in **Supplementary Figure 1a**. The numbers are the nanogram (ng) plasmid amounts co-transfected per sample in a 96-well setup. In case of compounds amounts are reported in µM, for DMSO as percentage of total reaction volume.

	T122					T130a					T27b					T125				
mCerulean-TRE-mCherry-T122x4 (pBH0112)	50																			
mCerulean-TRE-mCherry-T130ax4 (pBH0075)						50														
mCerulean-TRE-mCherry-T27bx4 (pBH0076)											50									
mCerulean-TRE-mCherry-T125ax4 (pBH0077)																50				
mCerulean-TRE-mCherry-T18ax4 (pBH0078)																				
mCerulean-TRE-mCherry-T7x4 (pBH0079)																				
mCerulean-TRE-mCherry-T20ax4 (pBH0080)																				
mCerulean-TRE-mCherry-T21x4 (pBH0081)																				
mCerulean-TRE-mCherry-Tlet7bx4 (pBH0082)																				
mCerulean-TRE-mCherry-T16x4 (pBH0083)																				
mCerulean-TRE-mCherry-T24x4 (pBH0084)																				
mCerulean-TRE-mCherry-TFF5x4 (pBH0091)																				
CMV-tTA (pBA166)	25					25					25					25				
Junk-DNA, Ubi-empty-NOS (pDT7004)	100					100					100					100				
DMSO	1					1					1					1				
Enoxacin		10					10					10					10			
PLL			10					10					10					10		
NSC158959				10					10					10					10	
NSC308847					10					10					10					10

Supplementary Table 18 Continuation

	T18a					T7					T20a					T21				
mCerulean-TRE-mCherry-T122x4 (pBH0112)																				
mCerulean-TRE-mCherry-T130ax4 (pBH0075)																				
mCerulean-TRE-mCherry-T27bx4 (pBH0076)																				
mCerulean-TRE-mCherry-T125ax4 (pBH0077)																				
mCerulean-TRE-mCherry-T18ax4 (pBH0078)	50																			
mCerulean-TRE-mCherry-T7x4 (pBH0079)						50														
mCerulean-TRE-mCherry-T20ax4 (pBH0080)											50									
mCerulean-TRE-mCherry-T21x4 (pBH0081)																50				
mCerulean-TRE-mCherry-Tlet7bx4 (pBH0082)																				
mCerulean-TRE-mCherry-T16x4 (pBH0083)																				
mCerulean-TRE-mCherry-T24x4 (pBH0084)																				
mCerulean-TRE-mCherry-TFF5x4 (pBH0091)																				
CMV-tTA (pBA166)	25					25					25					25				
Junk-DNA, Ubi-empty-NOS (pDT7004)	100					100					100					100				
DMSO	1					1					1					1				
Enoxacin		10					10					10					10			
PLL			10					10					10					10		
NSC158959				10					10					10					10	
NSC308847					10					10					10					10

Supplementary Table 18 Continuation

	Tlet7					T16					T24					TFF5				
mCerulean-TRE-mCherry-T122x4 (pBH0112)																				
mCerulean-TRE-mCherry-T130ax4 (pBH0075)																				
mCerulean-TRE-mCherry-T27bx4 (pBH0076)																				
mCerulean-TRE-mCherry-T125ax4 (pBH0077)																				
mCerulean-TRE-mCherry-T18ax4 (pBH0078)																				
mCerulean-TRE-mCherry-T7x4 (pBH0079)																				
mCerulean-TRE-mCherry-T20ax4 (pBH0080)																				
mCerulean-TRE-mCherry-T21x4 (pBH0081)																				
mCerulean-TRE-mCherry-Tlet7bx4 (pBH0082)	50																			
mCerulean-TRE-mCherry-T16x4 (pBH0083)						50														
mCerulean-TRE-mCherry-T24x4 (pBH0084)											50									
mCerulean-TRE-mCherry-TFF5x4 (pBH0091)																50				
CMV-tTA (pBA166)	25					25					25					25				
Junk-DNA, Ubi-empty-NOS (pDT7004)	100					100					100					100				
DMSO	1					1					1					1				
Enoxacin		10					10					10					10			
PLL			10					10					10					10		
NSC158959				10					10					10					10	
NSC308847					10					10					10					10

Supplementary Table 19 Transfection setup of experiment shown in **Supplementary Figure 1b**. The numbers are the nanogram (ng) plasmid amounts co-transfected per sample in a 24-well setup. In case of compounds amounts are reported in µM, for DMSO as percentage of total reaction volume.

	T122									w/o target								
	DMSO			NSC158959			NSC308847			DMSO			NSC158959			NSC308847		
	low	med	high	low	med	high	low	med	high	low	med	high	low	med	high	low	med	high
PpLuc-TRE-RrLuc-T122x4 (pBH0161)	100	100	100	100	100	100	100	100	100									
PpLuc-TRE-RrLuc (pBH0157)										100	100	100	100	100	100	100	100	100
CMV-tTA (pBA166)	50	50	50	50	50	50	50	50	50	50	50	50	50	50	50	50	50	50
Junk-DNA, Ubi-empty-NOS (pDT7004)	650	650	650	650	650	650	650	650	650	650	650	650	650	650	650	650	650	650
DMSO	0.1	0.5	1							0.1	0.5	1						
NSC158959				1	5	10							1	5	10			
NSC308847							1	5	10							1	5	10

Supplementary Table 20 Transfection setup of the experiment shown in **Supplementary Figure 1c**. The numbers are the nanogram (ng) plasmid amounts co-transfected per sample in a 96-well setup. In case of compounds amounts are reported in µM, for DMSO as percentage of total reaction volume.

	Ubx4-mcherry-T122
mCerulean-TRE- Ubiqutinx4-mCherry-PEST-T122x4 (pBH0281)	25
CMV-rtTA-TFF5x4 (pZ091)	12.5
Lac-op free Junk-DNA Ubi-Nos (pBH0265)	137.5
DMSO	1%

Supplementary Table 21 Transfection setup of the experiment shown in **Supplementary Figure 1d**. The numbers are the nanogram (ng) plasmid amounts co-transfected per sample in a 96-well setup. In case of compounds amounts are reported in µM, for DMSO as percentage of total reaction volume.

	PEST-TFF5x4						PEST-T122x4					
mCerulean-TRE-mCherry-PEST-TFF5x4 (pBH0287)	25	25	25	25	25	25						
mCerulean-TRE-mCherry-PEST-T122x4 (pBH0286)							25	25	25	25	25	25
CMV-rtTA-TFF5x4 (pZ091)	12.5	12.5	12.5	12.5	12.5	12.5	12.5	12.5	12.5	12.5	12.5	12.5
Lac-op free Junk-DNA Ubi-Nos (pBH0265)	137.5	137.5	137.5	137.5	137.5	137.5	137.5	137.5	137.5	137.5	137.5	137.5
DMSO	0.1%	0.5%	1.0%				0.1%	0.5%	1.0%			
NSC308847				1	5	10				1	5	10

Supplementary Table 22 Transfection setup of the experiment shown in **Supplementary Figure 1e**. The numbers are the nanogram (ng) plasmid amounts co-transfected per sample in a 96-well setup. In case of compounds amounts are reported in µM, for DMSO as percentage of total reaction volume.

	DMSO	NSC308847
mCerulean-TRE-mCherry-PEST-T122x4 (pBH0286)	25	25
CMV-rtTA-TFF5x4 (pZ091)	12.5	12.5
Lac-op free Junk-DNA Ubi-Nos (pBH0265)	137.5	137.5
DMSO	1.0%	
NSC308847		10

Supplementary Table 23 Transfection setup of experiment shown in **Supplementary Figure 1f** The numbers are the nanogram (ng) plasmid amounts co-transfected per sample in a 24-well setup. In case of compounds amounts are reported in μM, for DMSO as percentage of total reaction volume.

			4h post transfection					
	Reference		DMSO		NSC158959		NSC308847	
CMV-PpLuc (pZ003)	100		100		100		100	
CMV-RrLuc (pZ005)		100		100		100		100
Junk-DNA, Ubi-empty-NOS (pDT7004)	500	500	500	500	500	500	500	500
DMSO			1	1				
NSC158959					10	10		
NSC308847							10	10
Addition time post transfection			4h	4h	4h	4h	4h	4h

	48h post transfection						Direct to lysate					
	DMSO		NSC158959		NSC308847		DMSO		NSC158959		NSC308847	
CMV-PpLuc (pZ003)	100		100		100		100		100		100	
CMV-RrLuc (pZ005)		100		100		100		100		100		100
Junk-DNA, Ubi-empty-NOS (pDT7004)	500	500	500	500	500	500	500	500	500	500	500	500
DMSO	1	1					1	1				
NSC158959			10	10					10	10		
NSC308847					10	10					10	10
Addition time post transfection	48h	48h	48h	48h	48h	48h	48.5h	48.5h	48.5h	48.5h	48.5h	48.5h

Supplementary Table 24 Transfection setup of the experiment shown in **Supplementary Figure 1g**. The numbers are the nanogram (ng) plasmid amounts co-transfected per sample in a 24-well setup. In case of compounds amounts are reported in µM, for DMSO as percentage of total reaction volume.

	TFF5x4				T122x1				T122x4			
PpLuc-TRE-RrLuc-TFF5x4 (pBH0159)	100	100	100	100								
PpLuc-TRE-RrLuc-T122x1 (pBH0160)					100	100	100	100				
PpLuc-TRE-RrLuc-T122x4 (pBH0161)									100	100	100	100
CMV-rtTA-TFF5x4 (pZ091)	50	50	50	50	50	50	50	50	50	50	50	50
Lac-op free Junk-DNA Ubi-Nos (pBH0265)	550	550	550	550	550	550	550	550	550	550	550	550
DMSO	1.0%				1.0%				1.0%			
NSC5476		0.1	1	10		0.1	1	10		0.1	1	10

Supplementary Table 25 Transfection setup of the experiment shown in **Supplementary Figure 1h**. The numbers are the nanogram (ng) plasmid amounts co-transfected per sample in a 24-well setup. In case of compounds amounts are reported in µM, for DMSO as percentage of total reaction volume.

	Reference		direct additon to lysate				addtion 48 h post TF				addtion 4 h post TF			
CMV-PpLuc (pZ003)	100		100	100			100	100			100	100		
CMV-RrLuc (pZ005)		100			100	100			100	100			100	100
Lac-op free Junk-DNA Ubi-Nos (pBH0265)	600	600	600	600	600	600	600	600	600	600	600	600	600	600
DMSO			1.0%		1.0%		1.0%		1.0%		1.0%		1.0%	
NSC5476				10		10		10		10		10		10

Supplementary Table 26 Transfection setup of experiment shown in **Supplementary Figure 1i**. The numbers are the nanogram (ng) plasmid amounts co-transfected per sample in a 24-well setup. In case of LNAs/ mimics, the amounts are reported in nM final concentration.

	Reference				Exp-5, 200 ng				Exp-5, 500 ng				miR-DGCR8, 200 ng			
	let-7b	122	146a	FF5	let-7b	122	146a	FF5	let-7b	122	146a	FF5	let-7b	122	146a	FF5
mCerulean-TRE-mCherry-Tlet7bx4 (pBH0082)	100				100				100				100			
mCerulean-TRE-mCherry-T122x4 (pBH0112)		100				100				100				100		
mCerulean-TRE-mCherry-T146ax4 (pBH0195)			100				100				100				100	
mCerulean-TRE-mCherry-TFF4x4 (pBH0091)				100				100				100				100
CMV-Exportin-5 (pBH0151)					200	200	200	200	550	550	550	550				
CMV-Neo-miR-30 Stem loop- anti-DGCR8 miRNA (pBH0175)													200	200	200	200
siNegCtrl																
siDicer																
CMV-rtTA-TFF5 (pZ091)	50	50	50	50	50	50	50	50	50	50	50	50	50	50	50	50
Junk-DNA, Ubi-empty-NOS (pDT7004)	550	550	550	550	350	350	350	350					350	350	350	350

	miR-DGCR8, 500 ng				siDicer0, 0 nM				siDicer0, 10 nM				siDicer0, 30 nM			
	let-7b	122	146a	FF5	let-7b	122	146a	FF5	let-7b	122	146a	FF5	let-7b	122	146a	FF5
mCerulean-TRE-mCherry-Tlet7bx4 (pBH0082)	100				100				100				100			
mCerulean-TRE-mCherry-T122x4 (pBH0112)		100				100				100				100		
mCerulean-TRE-mCherry-T146ax4 (pBH0195)			100				100				100				100	
mCerulean-TRE-mCherry-TFF4x4 (pBH0091)				100				100				100				100
CMV-Exportin-5 (pBH0151)																
CMV-Neo-miR-30 Stem loop- anti-DGCR8 miRNA (pBH0175)	550	550	550	550												
siNegCtrl					30	30	30	30	20	20	20	20				
siDicer									10	10	10	10	30	30	30	30
CMV-rtTA-TFF5 (pZ091)	50	50	50	50	50	50	50	50	50	50	50	50	50	50	50	50
Junk-DNA, Ubi-empty-NOS (pDT7004)					550	550	550	550	550	550	550	550	550	550	550	550

Supplementary Table 27 Transfection setup of the experiment shown in **Supplementary Figure 1j**. The numbers are the nanogram (ng) plasmid amounts co-transfected per sample in a 96-well setup. In case of LNAs/ mimics, the amounts are reported in nM final concentration.

	siNeg.Ctrl											
mCerulean-TRE-mCherry-T18ax4 (pBH0078)	25											
mCerulean-TRE-mCherry-T7x4 (pBH0079)		25										
mCerulean-TRE-mCherry-T20ax4 (pBH0080)			25									
mCerulean-TRE-mCherry-T21x4 (pBH0081)				25								
mCerulean-TRE-mCherry-T21x4 (pBH0082)					25							
mCerulean-TRE-mCherry-T16x4 (pBH0083)						25						
mCerulean-TRE-mCherry-TFF5x4 (pBH0091)							25					
mCerulean-TRE-mCherry-T122x4 (pBH0112)								25				
mCerulean-TRE-mCherry-T146ax4 (pBH0195)									25			
mCerulean-TRE-mCherry-T141x4 (pBH0267)										25		
mCerulean-TRE-Ubx4-mCherry-T122x4 (pBH0281)											25	
AmCyan-TRE-DsRed-T17x4 (pZ145)												25
CMV-rtTA-TFF5x4 (pZ091)	12.5	12.5	12.5	12.5	12.5	12.5	12.5	12.5	12.5	12.5	12.5	12.5
Lac-op free Junk-DNA Ubi-Nos (pBH0265)	137.5	137.5	137.5	137.5	137.5	137.5	137.5	137.5	137.5	137.5	137.5	137.5
siNeg.Ctrl.	20	20	20	20	20	20	20	20	20	20	20	20
siDrosha												
siDGCR8												
siDicer												
siTRBP4												

Supplementary Table 27 Continuation

	siDrosha											
mCerulean-TRE-mCherry-T18ax4 (pBH0078)	25											
mCerulean-TRE-mCherry-T7x4 (pBH0079)		25										
mCerulean-TRE-mCherry-T20ax4 (pBH0080)			25									
mCerulean-TRE-mCherry-T21x4 (pBH0081)				25								
mCerulean-TRE-mCherry-T21x4 (pBH0082)					25							
mCerulean-TRE-mCherry-T16x4 (pBH0083)						25						
mCerulean-TRE-mCherry-TFF5x4 (pBH0091)							25					
mCerulean-TRE-mCherry-T122x4 (pBH0112)								25				
mCerulean-TRE-mCherry-T146ax4 (pBH0195)									25			
mCerulean-TRE-mCherry-T141x4 (pBH0267)										25		
mCerulean-TRE-Ubx4-mCherry-T122x4 (pBH0281)											25	
AmCyan-TRE-DsRed-T17x4 (pZ145)												25
CMV-rtTA-TFF5x4 (pZ091)	12.5	12.5	12.5	12.5	12.5	12.5	12.5	12.5	12.5	12.5	12.5	12.5
Lac-op free Junk-DNA Ubi-Nos (pBH0265)	137.5	137.5	137.5	137.5	137.5	137.5	137.5	137.5	137.5	137.5	137.5	137.5
siNeg.Ctrl.												
siDrosha	20	20	20	20	20	20	20	20	20	20	20	20
siDGCR8												
siDicer												
siTRBP4												

Supplementary Table 27 Continuation

	siDGCR8											
mCerulean-TRE-mCherry-T18ax4 (pBH0078)	25											
mCerulean-TRE-mCherry-T7x4 (pBH0079)		25										
mCerulean-TRE-mCherry-T20ax4 (pBH0080)			25									
mCerulean-TRE-mCherry-T21x4 (pBH0081)				25								
mCerulean-TRE-mCherry-T21x4 (pBH0082)					25							
mCerulean-TRE-mCherry-T16x4 (pBH0083)						25						
mCerulean-TRE-mCherry-TFF5x4 (pBH0091)							25					
mCerulean-TRE-mCherry-T122x4 (pBH0112)								25				
mCerulean-TRE-mCherry-T146ax4 (pBH0195)									25			
mCerulean-TRE-mCherry-T141x4 (pBH0267)										25		
mCerulean-TRE-Ubx4-mCherry-T122x4 (pBH0281)											25	
AmCyan-TRE-DsRed-T17x4 (pZ145)												25
CMV-rtTA-TFF5x4 (pZ091)	12.5	12.5	12.5	12.5	12.5	12.5	12.5	12.5	12.5	12.5	12.5	12.5
Lac-op free Junk-DNA Ubi-Nos (pBH0265)	137.5	137.5	137.5	137.5	137.5	137.5	137.5	137.5	137.5	137.5	137.5	137.5
siNeg.Ctrl.												
siDrosha												
siDGCR8	20	20	20	20	20	20	20	20	20	20	20	20
siDicer												
siTRBP4												

Supplementary Table 27 Continuation

	siDicer											
mCerulean-TRE-mCherry-T18ax4 (pBH0078)	25											
mCerulean-TRE-mCherry-T7x4 (pBH0079)		25										
mCerulean-TRE-mCherry-T20ax4 (pBH0080)			25									
mCerulean-TRE-mCherry-T21x4 (pBH0081)				25								
mCerulean-TRE-mCherry-T21x4 (pBH0082)					25							
mCerulean-TRE-mCherry-T16x4 (pBH0083)						25						
mCerulean-TRE-mCherry-TFF5x4 (pBH0091)							25					
mCerulean-TRE-mCherry-T122x4 (pBH0112)								25				
mCerulean-TRE-mCherry-T146ax4 (pBH0195)									25			
mCerulean-TRE-mCherry-T141x4 (pBH0267)										25		
mCerulean-TRE-Ubx4-mCherry-T122x4 (pBH0281)											25	
AmCyan-TRE-DsRed-T17x4 (pZ145)												25
CMV-rtTA-TFF5x4 (pZ091)	12.5	12.5	12.5	12.5	12.5	12.5	12.5	12.5	12.5	12.5	12.5	12.5
Lac-op free Junk-DNA Ubi-Nos (pBH0265)	137.5	137.5	137.5	137.5	137.5	137.5	137.5	137.5	137.5	137.5	137.5	137.5
siNeg.Ctrl.												
siDrosha												
siDGCR8												
siDicer	20	20	20	20	20	20	20	20	20	20	20	20
siTRBP4												

Supplementary Table 27 Continuation

	siTRBP4											
mCerulean-TRE-mCherry-T18ax4 (pBH0078)	25											
mCerulean-TRE-mCherry-T7x4 (pBH0079)		25										
mCerulean-TRE-mCherry-T20ax4 (pBH0080)			25									
mCerulean-TRE-mCherry-T21x4 (pBH0081)				25								
mCerulean-TRE-mCherry-T21x4 (pBH0082)					25							
mCerulean-TRE-mCherry-T16x4 (pBH0083)						25						
mCerulean-TRE-mCherry-TFF5x4 (pBH0091)							25					
mCerulean-TRE-mCherry-T122x4 (pBH0112)								25				
mCerulean-TRE-mCherry-T146ax4 (pBH0195)									25			
mCerulean-TRE-mCherry-T141x4 (pBH0267)										25		
mCerulean-TRE-Ubx4-mCherry-T122x4 (pBH0281)											25	
AmCyan-TRE-DsRed-T17x4 (pZ145)												25
CMV-rtTA-TFF5x4 (pZ091)	12.5	12.5	12.5	12.5	12.5	12.5	12.5	12.5	12.5	12.5	12.5	12.5
Lac-op free Junk-DNA Ubi-Nos (pBH0265)	137.5	137.5	137.5	137.5	137.5	137.5	137.5	137.5	137.5	137.5	137.5	137.5
siNeg.Ctrl.												
siDrosha												
siDGCR8												
siDicer												
siTRBP4	20	20	20	20	20	20	20	20	20	20	20	20

Supplementary Table 27 Continuation

	all											
mCerulean-TRE-mCherry-T18ax4 (pBH0078)	25											
mCerulean-TRE-mCherry-T7x4 (pBH0079)		25										
mCerulean-TRE-mCherry-T20ax4 (pBH0080)			25									
mCerulean-TRE-mCherry-T21x4 (pBH0081)				25								
mCerulean-TRE-mCherry-T21x4 (pBH0082)					25							
mCerulean-TRE-mCherry-T16x4 (pBH0083)						25						
mCerulean-TRE-mCherry-TFF5x4 (pBH0091)							25					
mCerulean-TRE-mCherry-T122x4 (pBH0112)								25				
mCerulean-TRE-mCherry-T146ax4 (pBH0195)									25			
mCerulean-TRE-mCherry-T141x4 (pBH0267)										25		
mCerulean-TRE-Ubx4-mCherry-T122x4 (pBH0281)											25	
AmCyan-TRE-DsRed-T17x4 (pZ145)												25
CMV-rtTA-TFF5x4 (pZ091)	12.5	12.5	12.5	12.5	12.5	12.5	12.5	12.5	12.5	12.5	12.5	12.5
Lac-op free Junk-DNA Ubi-Nos (pBH0265)	137.5	137.5	137.5	137.5	137.5	137.5	137.5	137.5	137.5	137.5	137.5	137.5
siNeg.Ctrl.												
siDrosha	5	5	5	5	5	5	5	5	5	5	5	5
siDGCR8	5	5	5	5	5	5	5	5	5	5	5	5
siDicer	5	5	5	5	5	5	5	5	5	5	5	5
siTRBP4	5	5	5	5	5	5	5	5	5	5	5	5

Supplementary Table 28 Transfection setup of experiment shown in **Supplementary Figure 2a**. The numbers are the nanogram (ng) plasmid amounts co-transfected per sample in a 96-well setup. In case of LNAs/ mimics, the amounts are reported in nM final concentration.

	T145		T375		T146a		141		T122	
mCerulean-TRE-mCherry-T145x4 (pBH0193)	25	25								
mCerulean-TRE-mCherry-T375x4 (pBH0194)			25	25						
mCerulean-TRE-mCherry-T146ax4 (pBH0195)					25	25				
mCerulean-TRE-mCherry-141x4 (pBH0267)							25	25		
mCerulean-TRE-mCherry-122x4 (pBH0112)										
CMV-rtTA-TFF5 (pZ091)	12.5	12.5	12.5	12.5	12.5	12.5	12.5	12.5	12.5	
Junk-DNA, Ubi-empty-NOS (pDT7004)	137.5	137.5	137.5	137.5	137.5	137.5	137.5	137.5	137.5	
Mim-Neg.Ctrl.	5		5		5		5		5	
Mim-145		5								
Mim-375				5						
Mim-146a						5				
Mim-141								5		
Mim-122										5

Supplementary Table 29 Transfection setup of experiment shown in **Supplementary Figure 2b**. The numbers are the nanogram (ng) plasmid amounts co-transfected per sample in a 96-well setup. In case of LNAs/ mimics, the amounts are reported in nM final concentration.

	TFF5	T21		T20a		T122	
mCerulean-TRE-mCherry-TFF5x4 (pBH0091)	25						
mCerulean-TRE-mCherry-T21x4 (pBH0081)		25	25				
mCerulean-TRE-mCherry-T20ax4 (pBH0080)				25	25		
mCerulean-TRE-mCherry-T122x4 (pBH0112)						25	25
CMV-rtTA-TFF5 (pZ091)	12.5	12.5	12.5	12.5	12.5	12.5	
Junk-DNA, Ubi-empty-NOS (pDT7004)	137.5	137.5	137.5	137.5	137.5	137.5	
LNA-Neg.Ctrl.	5	5		5		5	
LNA-21			5				
LNA-21					5		
LNA-122							5

Supplementary Table 30 Transfection setup of experiment shown in **Supplementary Figure 2c**. The numbers are the nanogram (ng) plasmid amounts co-transfected per sample in a 96-well setup. In case of LNAs/ mimics, the amounts are reported in nM final concentration.

<table>
<tr><td></td><td colspan="8">T21</td><td colspan="8">TFF4</td><td colspan="8">T141</td><td colspan="8">T146a</td></tr>
<tr><td>mCerulean-TRE-mCherry-T21x4 (pBH0082)</td><td colspan="8">25</td><td colspan="8"></td><td colspan="8"></td><td colspan="8"></td></tr>
<tr><td>mCerulean-TRE-mCherry-TFF4x4 (pBH0266)</td><td colspan="8"></td><td colspan="8">25</td><td colspan="8"></td><td colspan="8"></td></tr>
<tr><td>mCerulean-TRE-mCherry-T141x4 (pBH0267)</td><td colspan="8"></td><td colspan="8"></td><td colspan="8">25</td><td colspan="8"></td></tr>
<tr><td>mCerulean-TRE-mCherry-T146ax4 (pBH0195)</td><td colspan="8"></td><td colspan="8"></td><td colspan="8"></td><td colspan="8">25</td></tr>
<tr><td>mCerulean-TRE-mCherry-T122x4 (pBH0112)</td><td colspan="8"></td><td colspan="8"></td><td colspan="8"></td><td colspan="8"></td></tr>
<tr><td>mCerulean-TRE-mCherry-T20ax4 (pBH0080)</td><td colspan="8"></td><td colspan="8"></td><td colspan="8"></td><td colspan="8"></td></tr>
<tr><td>mCerulean-TRE-mCherry-23bx4 (pBH0273)</td><td colspan="8"></td><td colspan="8"></td><td colspan="8"></td><td colspan="8"></td></tr>
<tr><td>CMV-rtTA-TFF5x4 (pZ091)</td><td colspan="8">12.5</td><td colspan="8">12.5</td><td colspan="8">12.5</td><td colspan="8">12.5</td></tr>
<tr><td>Junk-DNA, Ubi-empty-NOS (pDT7004)</td><td colspan="8">137.5</td><td colspan="8">137.5</td><td colspan="8">137.5</td><td colspan="8">137.5</td></tr>
<tr><td>Mim-Neg.Ctrl.</td><td>5</td><td></td><td></td><td></td><td></td><td></td><td></td><td></td><td>5</td><td></td><td></td><td></td><td></td><td></td><td></td><td></td><td>5</td><td></td><td></td><td></td><td></td><td></td><td></td><td></td><td>5</td><td></td><td></td><td></td><td></td><td></td><td></td><td></td></tr>
<tr><td>Mim-21</td><td></td><td>5</td><td></td><td></td><td></td><td></td><td></td><td></td><td></td><td>5</td><td></td><td></td><td></td><td></td><td></td><td></td><td></td><td>5</td><td></td><td></td><td></td><td></td><td></td><td></td><td></td><td>5</td><td></td><td></td><td></td><td></td><td></td><td></td></tr>
<tr><td>siFF4</td><td></td><td></td><td>5</td><td></td><td></td><td></td><td></td><td></td><td></td><td></td><td>5</td><td></td><td></td><td></td><td></td><td></td><td></td><td></td><td>5</td><td></td><td></td><td></td><td></td><td></td><td></td><td></td><td>5</td><td></td><td></td><td></td><td></td><td></td></tr>
<tr><td>Mim-141</td><td></td><td></td><td></td><td>5</td><td></td><td></td><td></td><td></td><td></td><td></td><td></td><td>5</td><td></td><td></td><td></td><td></td><td></td><td></td><td></td><td>5</td><td></td><td></td><td></td><td></td><td></td><td></td><td></td><td>5</td><td></td><td></td><td></td><td></td></tr>
<tr><td>Mim-146a</td><td></td><td></td><td></td><td></td><td>5</td><td></td><td></td><td></td><td></td><td></td><td></td><td></td><td>5</td><td></td><td></td><td></td><td></td><td></td><td></td><td></td><td>5</td><td></td><td></td><td></td><td></td><td></td><td></td><td></td><td>5</td><td></td><td></td><td></td></tr>
<tr><td>Mim-122</td><td></td><td></td><td></td><td></td><td></td><td>5</td><td></td><td></td><td></td><td></td><td></td><td></td><td></td><td>5</td><td></td><td></td><td></td><td></td><td></td><td></td><td></td><td>5</td><td></td><td></td><td></td><td></td><td></td><td></td><td></td><td>5</td><td></td><td></td></tr>
<tr><td>Mim-20a</td><td></td><td></td><td></td><td></td><td></td><td></td><td>5</td><td></td><td></td><td></td><td></td><td></td><td></td><td></td><td>5</td><td></td><td></td><td></td><td></td><td></td><td></td><td></td><td>5</td><td></td><td></td><td></td><td></td><td></td><td></td><td></td><td>5</td><td></td></tr>
<tr><td>Mim-23b</td><td></td><td></td><td></td><td></td><td></td><td></td><td></td><td>5</td><td></td><td></td><td></td><td></td><td></td><td></td><td></td><td>5</td><td></td><td></td><td></td><td></td><td></td><td></td><td></td><td>5</td><td></td><td></td><td></td><td></td><td></td><td></td><td></td><td>5</td></tr>
</table>

Supplementary Table 30 Continuation

	T122								T20a								T23b							
mCerulean-TRE-mCherry-T21x4 (pBH0082)																								
mCerulean-TRE-mCherry-TFF4x4 (pBH0266)																								
mCerulean-TRE-mCherry-T141x4 (pBH0267)																								
mCerulean-TRE-mCherry-T146ax4 (pBH0195)																								
mCerulean-TRE-mCherry-T122x4 (pBH0112)	25																							
mCerulean-TRE-mCherry-T20ax4 (pBH0080)									25															
mCerulean-TRE-mCherry-23bx4 (pBH0273)																	25							
CMV-rtTA-TFF5x4 (pZ091)	12.5								12.5								12.5							
Junk-DNA, Ubi-empty-NOS (pDT7004)	137.5								137.5								137.5							
Mim-Neg.Ctrl.	5								5								5							
Mim-21		5								5								5						
siFF4			5								5								5					
Mim-141				5								5								5				
Mim-146a					5								5								5			
Mim-122						5								5								5		
Mim-20a							5								5								5	
Mim-23b								5								5								5

Supplementary Table 31 Transfection setup of experiment shown in **Supplementary Figure 2d**. The numbers are the nanogram (ng) plasmid amounts co-transfected per sample in a 96-well setup. In case of LNAs/ mimics, the amounts are reported in nM final concentration.

	T21								T20a								TFF4							
mCerulean-TRE-mCherry-T21x4 (pBH0082)	25																							
mCerulean-TRE-mCherry-T20ax4 (pBH0080)									25															
mCerulean-TRE-mCherry-TFF4x4 (pBH0266)																	25							
mCerulean-TRE-mCherry-T122x4 (pBH0112)																								
mCerulean-TRE-mCherry-23bx4 (pBH0273)																								
CMV-rtTA-TFF5x4 (pZ091)	12.5								12.5								12.5							
TRE-LacI-TFF5x4-miR-FF4 (pZ225)									12.5															
Junk-DNA, Ubi-empty-NOS (pDT7004)	137.5								125								137.5							
LNA-Neg.Ctrl.	5								5								5							
LNA-21		5								5								5						
LNA-20a			5								5								5					
LNA-FF4				5								5								5				
LNA-122					5								5								5			
LNA-23b						5								5								5		

Supplementary Table 31 Continuation

	T122								T23b							
mCerulean-TRE-mCherry-T21x4 (pBH0082)																
mCerulean-TRE-mCherry-T20ax4 (pBH0080)																
mCerulean-TRE-mCherry-TFF4x4 (pBH0266)																
mCerulean-TRE-mCherry-T122x4 (pBH0112)	25															
mCerulean-TRE-mCherry-23bx4 (pBH0273)									25							
CMV-rtTA-TFF5x4 (pZ091)	12.5								12.5							
TRE-LacI-TFF5x4-miR-FF4 (pZ225)																
Junk-DNA, Ubi-empty-NOS (pDT7004)	137.5								137.5							
LNA-Neg.Ctrl.	5								5							
LNA-21		5								5						
LNA-20a			5								5					
LNA-FF4				5								5				
LNA-122					5								5			
LNA-23b						5								5		

Supplementary Table 32 Transfection setup of experiment shown in **Supplementary Figure 2e**. The numbers are the nanogram (ng) plasmid amounts co-transfected per sample in a 24-well setup.

	TFF5						T20a						T21					
CMV-rtTA-TFF5x4 (pZ091)	0	0.93	2.78	8.33	25	75												
CMV-rtTA-T20ax4 (pBH0211)							0	0.93	2.78	8.33	25	75						
CMV-rtTA-T21x4 (pZ090)													0	0.93	2.78	8.33	25	75
mCerulean-TRE-MCS (pIM015)	100	100	100	100	100	100	100	100	100	100	100	100	100	100	100	100	100	100
Ef1α-mCherry (pKH026)	100	100	100	100	100	100	100	100	100	100	100	100	100	100	100	100	100	100
Lac-op free Junk-DNA Ubi-Nos (pBH0265)	500	499	497	491	475	425	500	499	497	491	475	425	500	499	497	491	475	425

Supplementary Table 33 Transfection setup of experiment shown in **Supplementary Figure 2f**. The numbers are the nanogram (ng) plasmid amounts co-transfected per sample in a 24-well setup.

	rtTA							PIT2						
mCerulean-TRE-mCherry-Spacer (pBH0074)	100	100	100	100	100	100	100							
CMV-rtTA-TFF5x4 (pZ091)	50	25	12.5	6.25	3.13	1.56	0							
mCerulean-PRE-mCherry-800 bp Spacer (pBH0107)								100	100	100	100	100	100	100
CAGop-PIT2-T146ax4-T141x4-TFF4x4 (pBH0256)								50	25	12.5	6.25	3.13	1.56	0
mCerulean-ERE-mCherry-800 bp Spacer (pBH0111)														
CAGop-ET-TFF4x4-T146ax4-T375x4-T145x4 (pBH0229)														
Junk-DNA, Ubi-empty-NOS (pDT7004)	450	475	488	494	497	498	500	450	475	488	494	497	498	500
Ef1α-mCitrine (pKH025):	100	100	100	100	100	100	100	100	100	100	100	100	100	100

	ET						
mCerulean-TRE-mCherry-Spacer (pBH0074)							
CMV-rtTA-TFF5x4 (pZ091)							
mCerulean-PRE-mCherry-800 bp Spacer (pBH0107)							
CAGop-PIT2-T146ax4-T141x4-TFF4x4 (pBH0256)							
mCerulean-ERE-mCherry-800 bp Spacer (pBH0111)	100	100	100	100	100	100	100
CAGop-ET-TFF4x4-T146ax4-T375x4-T145x4 (pBH0229)	50	25	12.5	6.25	3.13	1.56	0
Junk-DNA, Ubi-empty-NOS (pDT7004)	450	475	488	494	497	498	500
Ef1α-mCitrine (pKH025):	100	100	100	100	100	100	100

Supplementary Table 34 Transfection setup of the experiment in **Supplementary Figure 2g**. The numbers are the nanogram (ng) plasmid amounts co-transfected per sample in a 24-well setup.

	rtTA	PIT2	ET
mCerulean-TRE-mCherry-Spacer (pBH0074)	100		
CMV-rtTA-TFF5x4 (pZ091)	25		
mCerulean-PRE-mCherry-800 bp Spacer (pBH0107)		100	
CAGop-PIT2-T146ax4-T141x4-TFF4x4 (pBH0256)		25	
mCerulean-ERE-mCherry-800 bp Spacer (pBH0111)			100
CAGop-ET-TFF4x4-T146ax4-T375x4-T145x4 (pBH0229)			25
Junk-DNA, Ubi-empty-NOS (pDT7004)	475	475	475
Ef1α-mCitrine (pKH025):	100	100	100

Supplementary Table 35 Transfection setup of experiment shown in **Supplementary Figure 2h**. The numbers are the nanogram (ng) plasmid amounts co-transfected per sample in a 24-well setup. In case of LNAs/ mimics, the amounts are reported in nM final concentration.

	PIT								ET							
	TFF4-146a-141				T146a-141-TFF4				TFF4-146a-141				T146a-141-TFF4			
CAGop-Citrine-2A-PIT2-TFF4x4-T146ax4-T141x4 (pBH0232)	25	25	25	25												
CAGop-Citrine-2A-PIT2-T146ax4-T141x4-TFF4x4 (pBH0254)					25	25	25	25								
mCerulean-PRE-mCherry-T122x4 (pBH0182)	100	100	100	100	100	100	100	100								
CAGop-ET-TFF4x4-T146ax4-T141x4 (pBH0231)									25	25	25	25				
CAGop-ET-2A-Citrine-T146ax4-T141x4-TFF4x4 (pBH0250)													25	25	25	25
mCerulean-ERE-mCherry-T122x4 (pBH0177)									100	100	100	100	100	100	100	100
CMV-iRFP (pCS0012)	100	100	100	100	100	100	100	100	100	100	100	100	100	100	100	100
Junk-DNA	475	475	475	475	475	475	475	475	475	475	475	475	475	475	475	475
Mim-Neg.Ctrl.	5				5				5				5			
siFF4		5				5				5				5		
Mim-146a			5				5				5				5	
Mim-141				5				5				5				5

Supplementary Table 36 Transfection setup of experiment shown in **Supplementary Figure 2i**. The numbers are the nanogram (ng) plasmid amounts co-transfected per sample in a 24-well setup.

	T21					T20a					TFF5				
CMV-rtTA-T21x4 (pZ090)	25	25	25	25	25										
TRE-LacI-T21x4-miR-FF4 (pZ224)	0	25	50	100	150										
CMV-rtTA-T20ax4 (pBH0211)						25	25	25	25	25					
TRE-LacI-T20ax4-miR-FF4 (pBH0212)						0	25	50	100	150					
CMV-rtTA-TFF5x4 (pZ091)											25	25	25	25	25
TRE-LacI-TFF5x4-miR-FF4 (pZ225)											0	25	50	100	150
CAGop-Citrine-2A-PIT2-T146ax4-T141x4-TFF4x4 (pBH0254)	25	25	25	25	25	25	25	25	25	25	25	25	25	25	25
mCerulean-PRE-mCherry-T122x4 (pBH0182)	100	100	100	100	100	100	100	100	100	100	100	100	100	100	100
CMV-iRFP (pCS0012)	100	100	100	100	100	100	100	100	100	100	100	100	100	100	100
Lac-op free Junk-DNA Ubi-Nos (pBH0265)	450	425	400	350	300	450	425	400	350	300	450	425	400	350	300

Supplementary Table 37 Transfection setup of experiment shown in **Supplementary Figure 12**. The numbers are the nanogram (ng) plasmid amounts co-transfected per sample in a 24-well setup. In case of LNAs/ mimics, the amounts are reported in nM final concentration.

	DMSO	Tadalafil				Indinavir				Donepezil			
CMV-rtTA-T21x4 (pZ090)	3.125	3.125				3.125				3.125			
TRE-LacI-T21x4-miR-FF4 (pZ224)	3.125	3.125				3.125				3.125			
CMV-rtTA-T20ax4 (pBH0211)	3.125	3.125				3.125				3.125			
TRE-LacI-T20ax4-miR-FF4 (pBH0212)	3.125	3.125				3.125				3.125			
CAGop-PIT2-T146ax4-T141x4-TFF4x4 (pBH0256)	6.25	6.25				6.25				6.25			
TFF4x4-T141x4-T146ax4-mCerulean-PRE-mCherry-T122x4 (pBH0264)	100	100				100				100			
Ef1α-mCitrine (pKH025)	25	25				25				25			
Lac-op free Junk-DNA Ubi-Nos (pBH0265)	34.375	34.375				34.375				34.375			
Compound	1%	0.4				0.4				0.4			
Compound			2				2				2		
Compound				10				10				10	
Compound					50				50				50
Compound													
		Indatraline				Hexachlorophene				Chloroxine			
CMV-rtTA-T21x4 (pZ090)		3.125				3.125				3.125			
TRE-LacI-T21x4-miR-FF4 (pZ224)		3.125				3.125				3.125			
CMV-rtTA-T20ax4 (pBH0211)		3.125				3.125				3.125			
TRE-LacI-T20ax4-miR-FF4 (pBH0212)		3.125				3.125				3.125			
CAGop-PIT2-T146ax4-T141x4-TFF4x4 (pBH0256)		6.25				6.25				6.25			
TFF4x4-T141x4-T146ax4-mCerulean-PRE-mCherry-T122x4 (pBH0264)		100				100				100			
Ef1α-mCitrine (pKH025)		25				25				25			
Lac-op free Junk-DNA Ubi-Nos (pBH0265)		34.375				34.375				34.375			
Compound		0.4				0.4				0.4			
Compound			2				2				2		
Compound				10				10				10	
Compound					50				50				50

Supplementary Table 37 Continuation

	Levothyroxine				Lomerizine			
CMV-rtTA-T21x4 (pZ090)	3.125				3.125			
TRE-LacI-T21x4-miR-FF4 (pZ224)	3.125				3.125			
CMV-rtTA-T20ax4 (pBH0211)	3.125				3.125			
TRE-LacI-T20ax4-miR-FF4 (pBH0212)	3.125				3.125			
CAGop-PIT2-T146ax4-T141x4-TFF4x4 (pBH0256)	6.25				6.25			
TFF4x4-T141x4-T146ax4-mCerulean-PRE-mCherry-T122x4 (pBH0264)	100				100			
Ef1α-mCitrine (pKH025)	25				25			
Lac-op free Junk-DNA Ubi-Nos (pBH0265)	34.375				34.375			
Compound	0.4				0.4			
Compound		2				2		
Compound			10				10	
Compound				50				50
Compound								
	Digoxin				Mefloquine			
CMV-rtTA-T21x4 (pZ090)	3.125				3.125			
TRE-LacI-T21x4-miR-FF4 (pZ224)	3.125				3.125			
CMV-rtTA-T20ax4 (pBH0211)	3.125				3.125			
TRE-LacI-T20ax4-miR-FF4 (pBH0212)	3.125				3.125			
CAGop-PIT2-T146ax4-T141x4-TFF4x4 (pBH0256)	6.25				6.25			
TFF4x4-T141x4-T146ax4-mCerulean-PRE-mCherry-T122x4 (pBH0264)	100				100			
Ef1α-mCitrine (pKH025)	25				25			
Lac-op free Junk-DNA Ubi-Nos (pBH0265)	34.375				34.375			
Compound	0.4				0.4			
Compound		2				2		
Compound			10				10	
Compound				50				50

Supplementary Table 38 Transfection setup of the experiment shown in **Supplementary Figure 12**. The numbers are the nanogram (ng) plasmid amounts co-transfected per sample in a 96-well setup. In case of compounds amounts are reported in µM, for DMSO as percentage of total reaction volume.

	DMSO	Tadalafil				Indinavir				Donepezil				Indatraline				Hexachlorophene			
CMV-rtTA-TFF5x4 (pZ091)	12.5	12.5				12.5				12.5				12.5				12.5			
mCerulean-TRE-mCherry-T122x4 (pBH0112)	25	25				25				25				25				25			
Lac-op free Junk-DNA Ubi-Nos (pBH0265)	137.5	137.5				137.5				137.5				137.5				137.5			
Compound	1%	0.4				0.4				0.4				0.4				0.4			
Compound			2				2				2				2				2		
Compound				10				10				10				10				10	
Compound					50				50				50				50				50
Compound																					
		Chloroxine				Levothyroxine				Lomerizine				Digoxin				Mefloquine			
CMV-rtTA-TFF5x4 (pZ091)		12.5				12.5				12.5				12.5				12.5			
mCerulean-TRE-mCherry-T122x4 (pBH0112)		25				25				25				25				25			
Lac-op free Junk-DNA Ubi-Nos (pBH0265)		137.5				137.5				137.5				137.5				137.5			
Compound		0.4				0.4				0.4				0.4				0.4			
Compound			2				2				2				2				2		
Compound				10				10				10				10				10	
Compound					50				50				50				50				50

Supplementary Table 39 Transfection setup of the experiment shown in **Supplementary Figure 14**. The numbers are the nanogram (ng) plasmid amounts co-transfected per sample in a 96-well setup. In case of compounds amounts are reported in µM, for DMSO as percentage of total reaction volume.

	DMSO	Finasteride				2−Chloro-adenosine				Mestranol				Azasetron				Droperidol			
CMV-rtTA-TFF5x4 (pZ091)	12.5	12.5				12.5				12.5				12.5				12.5			
mCerulean-TRE-mCherry-T122x4 (pBH0112)	25	25				25				25				25				25			
Lac-op free Junk-DNA Ubi-Nos (pBH0265)	137.5	137.5				137.5				137.5				137.5				137.5			
Compound	1%	0.4				0.4				0.4				0.4				0.4			
Compound			2				2				2				2				2		
Compound				10				10				10				10				10	
Compound					50				50				50				50				50
Compound																					

		Diclofenac				Telithromycin			
CMV-rtTA-TFF5x4 (pZ091)		12.5				12.5			
mCerulean-TRE-mCherry-T122x4 (pBH0112)		25				25			
Lac-op free Junk-DNA Ubi-Nos (pBH0265)		137.5				137.5			
Compound		0.4				0.4			
Compound			2				2		
Compound				10				10	
Compound					50				50

Supplementary Table 39 Continuation

	DMSO	Finasteride				2−Chloro-adenosine				Mestranol			
CMV-rtTA-T21x4 (pZ090)	3.125	3.125				3.125				3.125			
TRE-LacI-T21x4-miR-FF4 (pZ224)	3.125	3.125				3.125				3.125			
CMV-rtTA-T20ax4 (pBH0211)	3.125	3.125				3.125				3.125			
TRE-LacI-T20ax4-miR-FF4 (pBH0212)	3.125	3.125				3.125				3.125			
CAGop-PIT2-T146ax4-T141x4-TFF4x4 (pBH0256)	6.25	6.25				6.25				6.25			
TFF4x4-T141x4-T146ax4-mCerulean-PRE-mCherry-T122x4 (pBH0264)	100	100				100				100			
Ef1α-mCitrine (pKH025)	25	25				25				25			
Lac-op free Junk-DNA Ubi-Nos (pBH0265)	34.375	34.375				34.375				34.375			
Compound	1%	0.4				0.4				0.4			
Compound			2				2				2		
Compound				10				10				10	
Compound					50				50				50

	Azasetron				Droperidol				Diclofenac				Telithromycin			
CMV-rtTA-T21x4 (pZ090)	3.125				3.125				3.125				3.125			
TRE-LacI-T21x4-miR-FF4 (pZ224)	3.125				3.125				3.125				3.125			
CMV-rtTA-T20ax4 (pBH0211)	3.125				3.125				3.125				3.125			
TRE-LacI-T20ax4-miR-FF4 (pBH0212)	3.125				3.125				3.125				3.125			
CAGop-PIT2-T146ax4-T141x4-TFF4x4 (pBH0256)	6.25				6.25				6.25				6.25			
TFF4x4-T141x4-T146ax4-mCerulean-PRE-mCherry-T122x4 (pBH0264)	100				100				100				100			
Ef1α-mCitrine (pKH025)	25				25				25				25			
Lac-op free Junk-DNA Ubi-Nos (pBH0265)	34.375				34.375				34.375				34.375			
Compound	0.4				0.4				0.4				0.4			
Compound		2				2				2				2		
Compound			10				10				10				10	
Compound				50				50				50				50

Supplementary Table 40 List of mimics, LNAs and siRNAs used

LNAs

Neg.Ctrl.	Commercial: Cat # 199020-00	Exiqon
miR-20a	Commercial: Cat # 426943-00	Exiqon
miR-21	Commercial: Cat # 426947-00	Exiqon
miR-23b	Commercial: Cat # 4101534-101	Exiqon
miR-122	Commercial: Cat # 426674-00	Exiqon
miR-FF4	Commercial: Custom order	Exiqon

Mimics

Neg.Ctrl.	Commercial: Cat # CN-001000-01-05	Thermo Scientific (GE Healthcare)
miR-20a	Commercial: Cat # C-300491-03	Thermo Scientific (GE Healthcare)
miR-21	Commercial: Cat # C-300492-03-0005	Thermo Scientific (GE Healthcare)
miR-23b	Commercial: Cat # C-300588-05-0005	Thermo Scientific (GE Healthcare)
miR-122	Commercial: Cat # C-300591-05	Thermo Scientific (GE Healthcare)
miR-141	Commercial: Cat # C-300608-03	Thermo Scientific (GE Healthcare)
miR-145	Commercial: Cat # C-300613-05	Thermo Scientific (GE Healthcare)
miR-146a	Commercial: Cat-# C-300630-03	Thermo Scientific (GE Healthcare)
miR-375	Commercial: Cat-# C-300683-05	Thermo Scientific (GE Healthcare)

siRNAs

siNeg.Ctrl.	Commercial: No Cat #:<br>5'-AGGUAGUGUAAUCGCCUUGtt-3', 3'-ttUCCAUCACAUUAGCGGAAC-5'	Microsynth
siDicer0[3]	5'-CAGCAUACUUUAUCGCCUUtt-3',<br>5'-AAGGCGAUAAAGUAUGCUGgg-3'	Microsynth
siDrosha[1]	5’-CGAGUAGGCUUCGUGACUUau-3',<br>3’-uuGCUCAUCCGAAGCACUGAA-5’	Microsynth
siDGCR8[1]	5’-GGAUGUAAAGAUUAGCGUGag-3’,<br>3’-uuCCUACAUUUCUAAUCGCAC-5’	Microsynth
siDicer[1]	5’-UGCUUGAAGCAGCUCUGGAuc-3',<br>3’-ugACGAACUUCGUCGAGACCU-5’	Microsynth
siTRBP4[1]	5’-GCUGCCUAGUAUAGAGCAAau-3',<br>3’-ccCGACGGAUCAUAUCUCGUU-5’	Microsynth
siFF4[4]	5'-GCUUGAAGUCUUUAAUUAAAuu-3',<br>3'-ggCGAACUUCAGAAAUUAAUUU-5'	Microsynth

Supplementary Table 41 Compounds of NIH clinical collection 1 & 2

#	Name	Trivial name
1	CPD000058423	BUPROPION HYDROCHLORIDE
2	CPD000471621	IRSOGLADINE MALEATE
3	CPD000466376	ACARBOSE
4	CPD000469294	BENPROPERINE PHOSPHATE
5	CPD000466378	
6	CPD000058926	
7	CPD000449280	Carvedilol
8	CPD000466379	LOMIFYLLINE
9	CPD000466380	PAZUFLOXACIN
10	CPD000466381	MIGLITOL
11	CPD000058373	
12	CPD000466345	OLANZAPINE
13	CPD000449297	Nefazodone
14	CPD000469185	Moxifloxacin hydrochloride
15	CPD000469186	NELFINAVIR MESYLATE
16	CPD000469187	PRAVASTATIN Sodium
17	CPD000466344	Topotecan Hydrochloride
18	CPD000466303	LEVETIRACETAM
19	CPD000469142	PRAMIPEXOLE HCl
20	CPD000466323	RISPERIDONE
21	CPD000469167	pioglitazone hydrochloride
22	CPD000469147	Cilastatin sodium
23	CPD000466348	ARGATROBAN
24	CPD000466327	VALDECOXIB
25	CPD000466346	NAFTOPIDIL
26	CPD000156231	Nobiletin
27	CPD000466304	FINASTERIDE
28	CPD000469145	ZOLPIDEM TARTRATE
29	CPD000048458	Viramune
30	CPD000466325	TOPIRAMATE
31	CPD000466350	VORICONAZOLE
32	CPD000469190	FENOLDOPAM MESYLATE
33	CPD000471612	ROSIGLITAZONE MALEATE
34	CPD000469191	ESCITALOPRAM OXALATE
35	CPD000058866	
36	CPD000466354	LATANOPROST
37	CPD000058576	
38	CPD000466298	Sertraline
39	CPD000466353	CALCIPOTRIOL
40	CPD000466308	EPIRUBICIN HYDROCHLORIDE
41	CPD000466329	BICALUTAMIDE
42	CPD000469192	BENIDIPINE HCl
43	CPD000466352	AMLEXANOX
44	CPD000469148	CERIVASTATIN SODIUM
45	CPD000466309	ICARIIN
46	CPD000466310	METHYLANDROSTENEDIOL
47	CPD000466307	TRIPTOLIDE
48	CPD000469170	ROSIGLITAZONE HCl
49	CPD000059106	
50	CPD000466392	OLIGOMYCIN C
51	CPD000469199	BENAZEPRIL HYDROCHLORIDE
52	CPD000058877	
53	CPD000059060	35212-22-7

54	CPD000058286	OXAPROZIN
55	CPD000058510	
56	CPD000469200	MOSAPRIDE CITRATE
57	CPD000466391	Isoquercitrin
58	CPD000058450	
59	CPD000469164	
60	CPD000466394	HYPEROSIDE
61	CPD000466322	RIFABUTIN
62	CPD000469141	ESMOLOL HYDROCHLORIDE
63	CPD000466321	TADALAFIL
64	CPD000058957	
65	CPD000058570	DOXORUBICIN HYDROCHLORIDE
66	CPD000469209	MOXONIDINE HCl
67	CPD000058302	
68	CPD000387024	PEFLOXACIN MESYLATE
69	CPD000469154	Venlafaxine hydrochloride
70	CPD000469592	Pantoprazole Sodium
71	CPD000469159	FLUTICASONE PROPIONATE
72	CPD000469161	Indinavir Sulfate
73	CPD000469160	Midazolam Hydrochloride
74	CPD000466319	LAMIVUDINE
75	CPD000469151	366-70-1
76	CPD000469280	ESOMEPRAZOLE Mg
77	CPD000059146	SULFASALAZINE
78	CPD000466313	TORASEMIDE
79	CPD000469156	tropisetronŒæhydrochloride
80	CPD000326795	Ranolazine dihydrochloride
81	CPD000338536	
82	CPD000466390	PIDOTIMOD
83	CPD000466386	RAMIPRIL
84	CPD000469284	FENPIVERINIUM BROMIDE
85	CPD000058610	19-Nortestosterone
86	CPD000466384	
87	CPD000059047	
88	CPD000048684	
89	CPD000466385	TROXIPIDE
90	CPD000466341	ACTARIT
91	CPD000469183	azelastine hydrochloride
92	CPD000466388	TOCAINIDE
93	CPD000499525	TAXIFOLIN-(+/-)
94	CPD000466387	LEVOFLOXACIN
95	CPD000469182	CEFATRIZINE PROPYLENE GLYCOL
96	CPD000466364	IDEBENONE
97	CPD000466366	LEVOSULPIRIDE
98	CPD000238142	
99	CPD000466343	LETROZOLE
100	CPD000469184	MEROPENEM
101	CPD000466339	ORLISTAT
102	CPD000469179	
103	CPD000059117	
104	CPD000469197	CETRAXATE HCl
105	CPD000149316	
106	CPD000058464	
107	CPD000059145	N-Ethyl-o-crotonotoluidide
108	CPD000472526	Amfebutamone
109	CPD000466340	ALFUZOSIN

110	CPD000449309	Amisulpride
111	CPD000469292	LOFEPRAMINE
112	CPD000466362	PEROSPIRONE HCl
113	CPD000059010	DOCETAXEL
114	CPD000387107	HONOKIOL
115	CPD000469196	TOLTERODINE TARTRATE
116	CPD000466363	CARMOFUR
117	CPD000469181	PAROXETINE
118	CPD000466337	OLMESARTAN MEDOXOMIL
119	CPD000469593	LOSARTAN Potassium
120	CPD000466338	TEMOZOLOMIDE
121	CPD000058528	
122	CPD000469195	tosufloxacin tosilate
123	CPD000466361	MECILLINAM
124	CPD000469177	Atomoxetine hydrochloride
125	CPD000466336	ARTESUNATE
126	CPD000058959	
127	CPD000469193	CEFPODOXIME PROXETIL
128	CPD000058803	Buflomedil HCl
129	CPD000012114	4-Chloro-N-(2-morpholin-4-yl-ethyl)-benzamide
130	CPD000466330	HALOMETASONE MONOHYDRATE
131	CPD000466357	TRICLABENDAZOLE
132	CPD000466331	ROFECOXIB
133	CPD000471619	BISOPROLOL FUMARATE
134	CPD000466334	EZETIMIBE
135	CPD000469176	TIAGABINE HCl
136	CPD000466355	idarubicin hydrochloride
137	CPD000466360	FLUBENDAZOLE
138	CPD000466356	TACROLIMUS
139	CPD000469208	VALACICLOVIR HYDROCHLORIDE
140	CPD000466382	CLARITHROMYCIN
141	CPD000466383	ARIPIPRAZOLE
142	CPD000471622	TRIMEBUTINE MALEATE
143	CPD000238198	
144	CPD000466370	NISOLDIPINE
145	CPD000466371	PICEID
146	CPD000149359	1-(2-Methyl-5-nitro-imidazol-1-yl)-propan-2-ol
147	CPD000466369	Nifekalant hydrochloride
148	CPD000466372	NATEGLINIDE
149	CPD000058691	
150	CPD000466374	ORMETOPRIM
151	CPD000466377	ZILEUTON
152	CPD000058350	
153	CPD000058918	
154	CPD000469293	OXICONAZOLE NITRATE
155	CPD000469235	KITASAMYCIN
156	CPD000466375	FAMCICLOVIR
157	CPD000326828	
158	CPD000466373	rufloxacin monohydrochloride
159	CPD000466389	TAXIFOLIN-(+)
160	CPD000469211	alosetron-monohydrochloride
161	CPD000059165	BESTATIN
162	CPD000469213	TOREMIFENE CITRATE
163	CPD000469214	GOSERELIN ACETATE
164	CPD000469212	SECOISOLARICIRESINOL
165	CPD000469217	RALTITREXED

166	CPD000469229	DOXAPRAM HYDROCHLORIDE
167	CPD000466294	RU 24969
168	CPD000112281	Brucine
169	CPD000059115	16502-01-5
170	CPD000058411	
171	CPD000469233	Palonosetron hydrochloride
172	CPD000058746	NAPROXEN SODIUM
173	CPD000058904	
174	CPD000058310	3-[3,5-DIBROMO-4-HYDROXYBENZOYL]-2-ETHYLBENZOFURAN
175	CPD000058300	
176	CPD000058701	
177	CPD000058715	
178	CPD000058273	
179	CPD000466922	Reichsteins substance S
180	CPD000059086	3-PYRIDINEMETHANOL
181	CPD000449283	Haloperidol
182	CPD000449279	Stiripentol
183	CPD000449303	Fluperlapine
184	CPD000058660	
185	CPD000112358	Homoveratrylamine
186	CPD000058194	
187	CPD000058741	XANTHINOL NICOTINATE
188	CPD000059111	SYNEPHRINE
189	CPD000058206	501-36-0
190	CPD000059093	118-71-8
191	CPD000059077	
192	CPD000059011	ENROFLOXACIN
193	CPD000058603	
194	CPD000058250	
195	CPD000059044	
196	CPD000469136	duloxetine hydrochloride
197	CPD000469155	VARDENAFIL CITRATE
198	CPD000469137	Ropivacaine hydrochloride
199	CPD000466301	ANASTROZOLE
200	CPD000058462	KETOTIFEN FUMARATE
201	CPD000058769	
202	CPD000466919	Pinacidil monohydrate
203	CPD000058266	
204	CPD000112269	
205	CPD000059045	92-84-2
206	CPD000058553	
207	CPD000469138	GranisetronŒæHydrochloride
208	CPD000466293	Rimcazole
209	CPD000466292	Nafadotride
210	CPD000058856	
211	CPD000471617	DEXCHLORPHENIRAMINE MALEATE
212	CPD000466288	Guanidine
213	CPD000466290	L-694,247
214	CPD000466284	AM-251
215	CPD000466289	HTMT
216	CPD000466286	Benzo[a]phenanthridine-10,11-diol, 5,6,6a,7,8,12b-hexahydro-, trans- [CAS]
217	CPD000466291	Methanesulfonamide, N-[4-[[1-[2-(6-methyl-2-pyridinyl)ethyl]-4-piperidinyl]carbonyl]phenyl]-, dihydrochloride [CAS]
218	CPD000466279	2H-Indol-2-one, 1,3-dihydro-1-phenyl-3,3-bis(4-pyridinylmethyl)- [CAS]
219	CPD000466920	Beclomethasone
220	CPD000058847	73590-58-6

221	CPD000469228	DOLASETRON MESYLATE
222	CPD000449310	Zolmitriptan
223	CPD000469223	TREMULACIN
224	CPD000469227	DACTINOMYCIN
225	CPD000449308	Tramadol
226	CPD000469226	CHLORDIAZEPOXIDE
227	CPD000469225	CEFIXIME TRIHYDRATE
228	CPD000469224	
229	CPD000469232	Lofexidine hydrochloride
230	CPD000469221	BALSALAZIDE
231	CPD000469220	OLOPATADINE HYDROCHLORIDE
232	CPD000469287	ITAVASTATIN Ca
233	CPD000058334	
234	CPD000058431	
235	CPD000469230	HOMOHARRINGTONINE
236	CPD000058318	50-22-6
237	CPD000471625	VECURONIUM BROMIDE
238	CPD000469219	TIBOLONE
239	CPD000058212	98-92-0
240	CPD000059131	
241	CPD000058612	
242	CPD000058726	
243	CPD000058572	1,1-DIMETHYL-4-PHENYLPIPERAZINIUM IODIDE
244	CPD000058507	
245	CPD000059128	72-33-3
246	CPD000059142	BENACTYZINE HYDROCHLORIDE
247	CPD000059100	
248	CPD000059158	79-43-6
249	CPD000466283	Altanserin
250	CPD000466281	Acetamide, 2-amino-N-(1-methyl-1,2-diphenylethyl)-, (+/-)- [CAS]
251	CPD000058420	
252	CPD000466311	
253	CPD000466285	Azasetron
254	CPD000466287	GR 89696
255	CPD000058773	DELTA1-HYDROCORTISONE 21-HEMISUCCINATE SODIUM SALT
256	CPD000058392	
257	CPD000058366	
258	CPD000469290	SAQUINAVIR MESYLATE
259	CPD000058970	60628-96-8
260	CPD000469158	SUMATRIPTAN SUCCINATE
261	CPD000466314	EXEMESTANE
262	CPD000466367	NITAZOXANIDE
263	CPD000058398	
264	CPD000471623	QUETIAPINE HEMIFUMARATE
265	CPD000112560	RUTIN
266	CPD000466317	PENCICLOVIR
267	CPD000466393	CALCITRIOL
268	CPD000469140	DIPHENOXYLATE
269	CPD000449307	Felbamate
270	CPD000058855	
271	CPD000035998	
272	CPD000466277	1H-Imidazole-5-carboxylic acid, 1-(1-phenylethyl)-, ethyl ester, (R)- [CAS]
273	CPD000466395	RITONAVIR
274	CPD000469210	vinorelbineŒætartrate
275	CPD000466335	LINEZOLID
276	CPD000469203	LOMERIZINE DiHCl

277	CPD000466351	EFAVIRENZ
278	CPD000466306	IRBESARTAN
279	CPD000466305	
280	CPD000238204	
281	CPD000440694	
282	CPD000469144	roxatidine acetateŒæhydrochloride
283	CPD000471616	DEXBROMPHENIRAMINE MALEATE
284	CPD000469168	anagrelide hydrochloride
285	CPD000471618	TEGASEROD MALEATE
286	CPD000058475	MILRINONE
287	CPD000466315	LEVOCETIRIZINE
288	CPD000326936	Citalopram
289	CPD000048468	Ticlopidine Hydrochloride
290	CPD000469165	sodiumŒæloxoprofen
291	CPD000466316	ZAFIRLUKAST
292	CPD000469152	Terbinafine hydrochloride
293	CPD000466320	ISRADIPINE
294	CPD000466318	VALSARTAN
295	CPD000449291	Piroxicam
296	CPD000469282	
297	CPD000449286	Physostigmine
298	CPD000466278	1H-Indole-2-propanoic acid, 1-[(4-chlorophenyl)methyl]-3-[(1,1-dimethylethyl)thio]-Alpha,Alpha-dimethyl-5-(1-methylethyl)- [CAS]
299	CPD000058436	562-10-7
300	CPD000449266	Milnacipran
301	CPD000449315	5-fluoro-2-pyrimidone
302	CPD000466271	Chlorpheniramine
303	CPD000466333	DOFETILIDE
304	CPD000471620	FORMOTEROL FUMARATE DIHYDRATE
305	CPD000525252	RIZATRIPTAN BENZOATE
306	CPD000466332	RIFAPENTINE
307	CPD000469178	LOTEPREDNOL ETABONATE
308	CPD000466359	ENALAPRILAT
309	CPD000449292	Donepezil
310	CPD000238177	
311	CPD000466365	
312	CPD000466326	
313	CPD000469143	ITOPRIDE HCl
314	CPD000466324	RIFAXIMIN
315	CPD000469188	MONTELUKAST SODIUM
316	CPD000058253	2',3'-DIDEOXYCYTIDINE
317	CPD000466276	1H-Imidazol-2-amine, N-(2,6-dichlorophenyl)-4,5-dihydro- [CAS]
318	CPD000466280	6H-Pyrido[2,3-b][1,4]benzodiazepin-6-one, 11-[[2-[(diethylamino)methyl]-1-piperidinyl]acetyl]-5,11-dihydro- [CAS]
319	CPD000449316	3'-deoxydenosine
320	CPD000449296	Ifenprodil
321	CPD000145728	5-Amino-2-hydroxy-benzoic acid
322	CPD000466269	Paroxetine
323	CPD000058465	LOBELINE HYDROCHLORIDE
324	CPD000449329	L-Ornithine, N5-[imino(methylamino)methyl]-[CAS]
325	CPD000058461	
326	CPD000449321	Oxiranecarboxylic acid, 2-[6-(4-chlorophenoxy)hexyl]-, ethyl ester- [CAS]
327	CPD000449288	Epigallocatechin gallate
328	CPD000449275	Raclopride
329	CPD000449271	Zacopride
330	CPD000449276	SKF 83566
331	CPD000449274	AM 404

332	CPD000449281	Nalbuphine
333	CPD000059053	PILOCARPINE HYDROCHLORIDE
334	CPD000058291	
335	CPD000042823	Flurbiprofen
336	CPD000059136	3-HYDROXY-1,2-DIMETHYL-4(1H)-PYRIDONE
337	CPD000058470	Loxapine
338	CPD000326694	d-3-Methoxy-N-methylmorphinan hydrobromide
339	CPD000449282	Duloxetine
340	CPD000449320	Glycine, N-[2-[(acetylthio)methyl]-1-oxo-3-phenylpropyl]-,phenylmethyl ester [CAS]
341	CPD000449318	Benzeneacetic acid, 2-[(2,6-dichlorophenyl)amino]-, monosodium salt [CAS]
342	CPD000058345	
343	CPD000058961	FAMOTIDINE
344	CPD000449299	SR 57227A
345	CPD000466270	Pancuronium
346	CPD000058175	443-48-1
347	CPD000449327	Benzeneacetic acid, Alpha-(hydroxymethyl)-, 9-methyl-3-oxa-9-azatricyclo[3.3.1.02,4]non-7-yl ester, [7(S)-(1Alpha,2 ,4 ,5Alpha,7)]- [CAS]
348	CPD000449323	Benzeneacetonitrile, Alpha-[3-[[2-(3,4-dimethoxyphenyl)ethyl]methylamino]propyl]-3,4-dimethoxy-Alpha-(1-methylethyl)-, (R)- [CAS]
349	CPD000449328	
350	CPD000449294	zucapsaicin
351	CPD000058513	SALBUTAMOL SULFATE
352	CPD000057879	(+/-)-Vesamicol hydrochloride
353	CPD000469289	Picrotin - Picrotoxinin
354	CPD000449268	Terazosin
355	CPD000449319	diphenylcyclopropenone
356	CPD000449326	4-Thiazolidinecarboxylic acid, 2-oxo-, (R)- [CAS]
357	CPD000466274	Mesoridazine
358	CPD000449313	3(2H)-Pyridazinone, 6-[4-(difluoromethoxy)-3-methoxyphenyl]- [CAS]
359	CPD000466275	10H-Phenothiazine, 2-chloro-10-[3-(4-methyl-1-piperazinyl)propyl- [CAS]
360	CPD000449322	1H-Cyclopenta[b]quinolin-9-amine, 2,3,5,6,7,8-hexahydro-, monohydrochloride- [CAS]
361	CPD000058306	CLOTRIMAZOLE
362	CPD000058255	79794-75-5
363	CPD000058500	Phenelzine sulfate
364	CPD000449311	Riluzole
365	CPD000449312	Naltrindole
366	CPD000449277	Nornicotine
367	CPD000449269	Bifemelane
368	CPD000449284	CGS 15943
369	CPD000449287	Cinanserin
370	CPD000449272	Cisapride
371	CPD000449273	Indatraline
372	CPD000058520	25332-39-2
373	CPD000449301	Prazosin
374	CPD000058525	URAPIDIL HYDROCHLORIDE
375	CPD000449278	(-)-Cotinine
376	CPD000058313	D-CYCLOSERINE
377	CPD000466268	Fluvoxamine
378	CPD000449270	Doxepin
379	CPD000059133	
380	CPD000058908	(+)-3-HYDROXY-N-METHYLMORPHINAN D-TARTRATE
381	CPD000058555	LY 171883
382	CPD000148117	Maprotiline hydrochloride
383	CPD000466272	Pizotyline

384	CPD000059126	BETA-ESTRADIOL
385	CPD000059046	N,N'-DIACETYL-1,6-DIAMINOHEXANE
386	CPD000058353	147-24-0
387	CPD000449267	Galanthamine
388	CPD000449290	Indomethacin
389	CPD000059171	TETRAETHYLTHIURAM DISULFIDE
390	CPD000449302	Piribedil
391	CPD000058460	
392	CPD000058623	
393	CPD000449325	Pyrazinecarboxamide, 3,5-diamino-N-(aminoiminomethyl)-6-chloro- [CAS]
394	CPD000059105	9-AMINO-1,2,3,4-TETRAHYDROACRIDINE HYDROCHLORIDE
395	CPD000058319	ETHYNYLESTRADIOL
396	CPD000449317	2(1H)-Pyrimidinone, 4-amino-1-y-D-arabinofuranosyl- [CAS]
397	CPD000449324	L-Glutamic acid, N-[4-[[(2,4-diamino-6-pteridinyl)methyl]methylamino]benzoyl]- [CAS]
398	CPD000449305	TFMPP
399	CPD000449298	Pramipexole
400	CPD000058189	
401	CPD000466297	SDM25N
402	CPD000466300	5-Nonyloxytryptamine
403	CPD000466296	SB 205607
404	CPD000058344	
405	CPD000238180	
406	CPD000468734	PD 81723
407	CPD000469222	
408	CPD000058445	
409	CPD000466299	Thiophene, 5-bromo-2-(4-fluorophenyl)-3-[4-(methylsulfonyl)phenyl]- [CAS]
410	CPD000466295	Salmeterol
411	CPD000326935	R(+)-SCH-23390 hydrochloride
412	CPD000059075	DEHYDROEPIANDROSTERONE
413	CPD000112594	Prostaglandin E1
414	CPD000058878	
415	CPD000468732	CCPA
416	CPD000468733	CGS 12066B
417	CPD000469153	VINDESINE SULFATE
418	CPD000058540	VINCRISTINE SULFATE
419	CPD000466342	LACIDIPINE
420	CPD000466347	
421	CPD000469285	AMPIROXICAM
422	CPD000466368	GLIMEPIRIDE
423	CPD000469198	Amlodipine
424	CPD000469174	RABEPRAZOLE
425	CPD000058704	CLOFAZIMINE
426	CPD000469166	Irinotecan hydrochloride
427	CPD000058469	103577-45-3
428	CPD000149358	8-Chloro-11-piperidin-4-ylidene-6,11-dihydro-5H-benzo[5,6]cyclohepta[1,2-b]pyridine
429	CPD000058772	1,3,5(10)-ESTRATRIEN-3-OL-17-ONE SULPHATE, SODIUM SALT
430	CPD000058481	
431	CPD000112002	
432	CPD000238156	Sibutramine
433	CPD000469632	
434	CPD000469231	
435	CPD000472527	Sibutramine hydrochloride
436	CPD000058410	
437	CPD000469633	8-Azaspiro[4.5]decane-7,9-dione, 8-[2-[[(2,3-dihydro-1,4-benzodioxin-2-yl)methyl]amino]ethyl]-, monomethanesulfonate [CAS]

438	CPD000469631	Adenosine, N-(2-hydroxycyclopentyl)-, (1S-trans)- [CAS]
439	CPD000058296	19774-82-4
440	CPD000336944	
441	CPD000469175	IMATINIB MESYLATE
442	CPD000468736	Metylperon
443	CPD000469594	Parecoxib sodium
444	CPD000058504	
445	CPD000471626	ATRACURIUM BESYLATE
446	CPD000469218	ARTEMETHER
447	CPD000058230	
448	CPD000058382	
449	CPD000059151	2078-54-8
450	CPD000058600	
451	CPD000058187	FLUTAMIDE
452	CPD000058299	49562-28-9
453	CPD000058202	54-31-9
454	CPD000038082	5-FLUOROURACIL
455	CPD000471860	Folic acid
456	CPD000653523	HYDROCORTISONE
457	CPD000653536	Cortell
458	CPD000058184	15687-27-1
459	CPD000040181	15962-46-6
460	CPD001906766	MINOCYCLINE HYDROCHLORIDE
461	CPD000058733	MICONAZOLE NITRATE
462	CPD000059134	METYRAPONE
463	CPD001317860	70458-96-7
464	CPD000058975	
465	CPD000058999	Disipal
466	CPD000058192	
467	CPD000059120	PINDOLOL
468	CPD000037139	55268-74-1
469	CPD000059104	1716-12-7
470	CPD000058326	PREDNISOLONE ACETATE
471	CPD000058379	Phenergan
472	CPD000058180	
473	CPD000718761	Prednisolone
474	CPD000058506	
475	CPD001227202	Prednisone
476	CPD000059161	DL-PENICILLAMINE
477	CPD000058579	PIPERACILLIN SODIUM
478	CPD000857275	Quinidine hydrochloride monohydrate
479	CPD000653467	56131-49-8
480	CPD001906767	RIFAMPICIN
481	CPD000058245	trans-Retinoic acid
482	CPD000471892	Spironolactone
483	CPD000035999	
484	CPD000058219	Tyzine
485	CPD000059176	L-THYROXINE
486	CPD000058515	
487	CPD000058403	URSODEOXYCHOLIC ACID
488	CPD000059064	80-08-0
489	CPD001370746	Symmetrel
490	CPD000058849	WARFARIN SODIUM
491	CPD000058394	59-66-5
492	CPD000059083	
493	CPD001906768	ATROPINE

494	CPD000058264	389-08-2
495	CPD001567029	3,5,3'-TRIIODOTHYRONINE
496	CPD000058284	
497	CPD000058368	Annoyltin
498	CPD000058613	Busulfan
499	CPD000058269	Chlorzoxazone
500	CPD000058429	Chlorothiazide
501	CPD001370748	Cimetidine
502	CPD000058433	
503	CPD000058364	94-20-2
504	CPD000058440	Bentyl
505	CPD000312779	Chloroxine
506	CPD000058723	
507	CPD001370749	Econazole Nitrate
508	CPD001370750	536-33-4
509	CPD000058719	
510	CPD000035778	58-93-5
511	CPD001370751	Vistaril Pamoate
512	CPD000058356	70-30-4
513	CPD000059082	Isoniazid
514	CPD000058729	Duvadilan
515	CPD000058267	ISOPROTERENOL HYDROCHLORIDE
516	CPD000471847	Triclosan
517	CPD000058188	61-68-7
518	CPD000058832	Cantil
519	CPD000058471	
520	CPD001370753	Methyldopa
521	CPD000058271	NITROFURANTOIN
522	CPD000058486	
523	CPD000058292	
524	CPD000059024	Nicotinic Acid
525	CPD000058817	Norflex
526	CPD001614498	Oxytetracycline hydrochloride
527	CPD000718771	
528	CPD000058714	58-14-0
529	CPD000058661	Pro-Banthine
530	CPD000058280	57-66-9
531	CPD001906769	PYRIDINE-2-ALDOXIME METHOCHLORIDE
532	CPD000058501	125-33-7
533	CPD000058275	Propylthiouracil
534	CPD000036662	98-96-4
535	CPD000059079	Pronestyl
536	CPD000037657	Sulfisoxazole
537	CPD000058223	
538	CPD000058173	Sulfacetamide
539	CPD000058991	Sulfinpyrazone
540	CPD000326718	
541	CPD001906770	TETRACYCLINE
542	CPD000058537	Theophylline
543	CPD000058363	64-77-7
544	CPD000059118	Triamterene
545	CPD000059081	Intropin
546	CPD000058416	AMOXAPINE
547	CPD000471872	Adenine 9-beta;-D-arabinofuranoside
548	CPD000036768	29122-68-7
549	CPD001491671	Tamoxifen

550	CPD000058418	
551	CPD000058745	
552	CPD000058254	69-09-0
553	CPD001491644	CefazolinÂ Sodium
554	CPD000059061	CAPTOPRIL
555	CPD000058372	305-03-3
556	CPD000058809	
557	CPD000058321	DANAZOL
558	CPD000058375	(+)-CIS-DILTIAZEM HYDROCHLORIDE
559	CPD001906774	DIGOXIN
560	CPD000058346	17-BETA-ESTRADIOL 17-VALERATE
561	CPD000058672	
562	CPD000058329	
563	CPD000042823	Flurbiprofen
564	CPD000058455	29094-61-9
565	CPD000058393	GEMFIBROZIL
566	CPD000058229	Glyburide
567	CPD000058328	HYDROCORTISONE HEMISUCCINATE
568	CPD000058829	26807-65-8
569	CPD001906775	Ipratropium Bromide
570	CPD000058388	113-52-0
571	CPD000058463	32780-64-6
572	CPD000058466	
573	CPD000058833	Pro-Amatine
574	CPD000653524	Medroxyprogesterone 17-acetate
575	CPD001906776	19-NORETHINDRONE ACETATE
576	CPD000499579	19-Norethindrone
577	CPD000059074	NICOTINE
578	CPD001456372	Cardene
579	CPD000058835	NABUMETONE
580	CPD000058490	
581	CPD000058605	Mestinon
582	CPD001453705	Rythmol
583	CPD001491654	Pfizerpen
584	CPD000499581	99-66-1
585	CPD000058821	
586	CPD000875264	Proxymetacaine
587	CPD000058766	NALOXONE HYDROCHLORIDE
588	CPD001906777	SPECTINOMYCIN DIHYDROCHLORIDE PENTAHYDRATE
589	CPD000058523	
590	CPD000058290	1156-19-0
591	CPD000058335	76-25-5
592	CPD001456519	S(-)-Timolol maleate
593	CPD000058170	THIABENDAZOLE
594	CPD000058380	
595	CPD000058181	
596	CPD001491672	Phylloquinone
597	CPD001491659	Eryped
598	CPD000058422	Dibenzyline
599	CPD000058693	6ALPHA-METHYL-11BETA-HYDROXYPROGESTERONE
600	CPD000058524	Thalidomide
601	CPD000857229	Aminolevulinic Acid
602	CPD001496929	Carbinoxamine Maleate
603	CPD001496930	Demeclocycline
604	CPD001496932	Westcort
605	CPD000449328	

606	CPD000058840	6-[2-ETHOXY-1-NAPHTHAMIDO]-PENICILLIN SODIUM SALT
607	CPD000875314	Primaquine Diphosphate
608	CPD001496934	Micropenin
609	CPD001550033	DOXYCYCLINE
610	CPD001233361	Beclomethasone dipropionate
611	CPD000058721	Cromolyn Sodium
612	CPD000149600	Priscoline
613	CPD000544948	Mercaptopurine
614	CPD000427366	Azathioprine
615	CPD000036735	Albendazole
616	CPD000718755	Griseofulvin
617	CPD000059006	
618	CPD001496938	Methazolamide
619	CPD001496939	Terbutaline Sulfate
620	CPD000471888	Mupirocin
621	CPD000058331	
622	CPD000875233	Mefloquine hydrochloride
623	CPD001496941	Floxuridine
624	CPD001563707	MITOXANTRONE
625	CPD001906784	ENALAPRIL MALEATE
626	CPD000058337	51333-22-3
627	CPD000466386	RAMIPRIL
628	CPD000718757	DEPO-MEDROL
629	CPD000058383	(+/-)-NOREPINEPHRINE HYDROCHLORIDE
630	CPD001491664	AMCINONIDE
631	CPD001317855	Clomid
632	CPD001819784	PhentolamineÂ Mono-hydrochloride
633	CPD000058874	FLUDARABINE
634	CPD000109709	Testosterone
635	CPD000471891	Isotretinoin
636	CPD000058376	Methimazole
637	CPD000596519	Zonisamide
638	CPD000058355	
639	CPD000036734	Mebendazole
640	CPD000058736	Meclizine hydrochloride
641	CPD000058451	
642	CPD000146393	Dilantin
643	CPD000059182	Miochol
644	CPD000326766	DantroleneÂ Sodium
645	CPD001227192	Dexamethasone
646	CPD000394012	Cogentin Mesylate
647	CPD000058324	
648	CPD000059219	
649	CPD000058785	Meclomen
650	CPD000471882	Fluconazole
651	CPD001453712	Metaproterenol
652	CPD000071170	Methoxsalen
653	CPD000058224	Chloramphenicol
654	CPD000499584	Tizanidine hydrochloride
655	CPD001453706	Paroxetine
656	CPD000550486	mirtazapine
657	CPD000010931	Etomidate
658	CPD000499578	Moban
659	CPD001453708	fluvastatin
660	CPD000058680	Urecholine
661	CPD001496804	Cefuroxime

662	CPD000718805	Cytoxan
663	CPD000550478	Eszopiclone
664	CPD000058802	Bendrofluazide
665	CPD000058508	82640-04-8
666	CPD000058351	30516-87-1
667	CPD000058365	
668	CPD001317850	Ampicillin Sodium
669	CPD000058800	
670	CPD000058707	AMOXICILLIN CRYSTALLINE
671	CPD000857209	(+/-)-Epinephrine hydrochloride
672	CPD000857239	5-Azacytidine
673	CPD000058186	Buspar
674	CPD000436311	
675	CPD000059121	Podofilox
676	CPD000058313	D-CYCLOSERINE
677	CPD000059124	CORTISONE ACETATE
678	CPD000058295	17321-77-6
679	CPD001227191	298-46-4
680	CPD000875213	Memantine hydrochloride
681	CPD000036827	
682	CPD000326711	
683	CPD000058438	
684	CPD000673569	STAVUDINE
685	CPD000097306	Doxazosin
686	CPD000058963	Minoxidil
687	CPD000059167	318-98-9
688	CPD001496943	Ribavirin
689	CPD000058309	Terazosin
690	CPD000058635	Chlorthalidone
691	CPD000058330	METHYLPREDNISOLONE
692	CPD001496977	Phenelzine
693	CPD000058767	
694	CPD000469282	
695	CPD000046147	Ethambutol
696	CPD001453715	Cetirizine
697	CPD000539527	DICLOXACILLIN SODIUM
698	CPD000718800	Meloxicam
699	CPD001906781	DAUNORUBICIN HYDROCHLORIDE
700	CPD001906779	RIFAPENTINE
701	CPD000274084	Penicillin V
702	CPD000043336	Gatifloxacin
703	CPD000550475	clopidogrel
704	CPD001551784	CEFOTAXIME SODIUM
705	CPD000466319	LAMIVUDINE
706	CPD001307702	Ondansetron
707	CPD000339803	Betamethasone
708	CPD000550473	Celecoxib
709	CPD000058778	4-(AMINOMETHYL)BENZENESULFONAMIDE ACETATE
710	CPD001906782	THIOTHIXENE
711	CPD000465669	Citalopram
712	CPD000471864	Azithromycin
713	CPD000673570	Lovastatin
714	CPD000326785	Aminoglutethimide
715	CPD000058452	
716	CPD001233272	FluniSOLIDe
717	CPD000058225	Acyclovir

718	CPD000058443	
719	CPD000718785	Simvastatin
720	CPD001227203	Rifabutin
721	CPD001496951	Felodipine
722	CPD000499582	QUINAPRIL HYDROCHLORIDE
723	CPD000499573	
724	CPD000718798	138452-21-8
725	CPD001563899	Fluorometholone
726	CPD000466298	Sertraline
727	CPD001566944	CARBIDOPA

Supplementary Note 1 for section "Experimental system"

We did not observe desired activity changes on bidirectional fluorescent reporters with previously-reported non-specific modulators, Enoxacin[5] and polylysine (PLL)[6], which could be due to the idiosyncrasies of the HuH-7 cell line (**Supplementary Fig. 1a**). For the compound NSC158959, previously reported to inhibit miR-122[7], we did not observe an effect on the miR-122 bidirectional fluorescent reporter (**Supplementary Fig. 1a)**. On the other hand, for the compound NSC308847, previously reported to increase miR-122 expression and reduce reporter expression[7], we found a 2-fold reduction of the miR-122 bidirectional fluorescence reporter consistent with increased miR-122 expression (**Supplementary Fig. 1a**). We next replaced fluorescent proteins in our bidirectional reporter construct with luciferases to increase assay sensitivity and retested the compounds with *Renilla* luciferase-based miR-122 activity reporter normalized to *Firefly* luciferase gene (**Supplementary Fig. 1b**). We reproduced the reported effect for NSC308847[7]: the miR-122 sensor is knocked down consistent with elevated miR-122 activity, while the sensor with scrambled target is only slightly reduced. Quantitatively, the effect is stronger with luciferase reporters compared to fluorescent ones. We hypothesized that this was due to shorter half-lives of the luciferase proteins and constructed mCherry-PEST[8] and Ubiquitin x4-mCherry-PEST reporters in order to have fluorescent reporter versions with comparable half-life. For Ubiquitin x4-mCherry-PEST with T122 target we were no longer able to measure mCherry fluorescence (**Supplementary Fig. 1c**), and we repeated the NSC308847 measurements with the mCherry-PEST reporter. However, we were not able to detect the effects observed with the luciferase reporter. On the contrary, we saw apparent de-repression of the mCherry-PEST reporter at 10 µM concentration (**Supplementary Fig. 1d**). Yet, thorough analysis of the scatter plots suggested that this resulted from toxic effects at higher drug concentrations (**Supplementary Fig. 1e**). Contrary to these findings, the results of the fluorescent and luciferase-based assays with NSC158959 did not show consistent behavior: while the fluorescent reporters did not suggest an effect (see above), the *Renilla*-to-*Firefly* ratiometric luciferase readout was elevated when furnished with miR-122 as well as with scrambled

binding sites. We therefore hypothesized, that NSC158959 could be targeting one of the luciferases directly or interfere with the luciferase-catalyzed reaction. We tested this hypotheses by measuring *Firefly* and *Renilla* luciferases devoid of any miRNA targets separately and added the compounds NSC308847 and NSC158959 to samples at different time points: (i) Directly to the cell lysate immediately prior to photon counting; (ii) directly to cell growth media 48 h after cultivation and right before cell lysis; and (iii) 4 h post transfection, which is a typical time point for adding a drug (**Supplementary Fig. 1f**). The expression of *Renilla* luciferase did not change significantly in response to either compound; only NSC308847 had a minor effect after 48 h, which could be due to modest cytotoxicity. *Firefly* luciferase on the other hand was strongly affected by NSC158959 but not NSC308847; in particular, addition of NSC158959 at 4 hours or its direct addition to the luciferase assay reduced *Firefly* photon count, elevating the apparent ratiometric signal between *Renilla* and *Firefly* for NSC158959. Because this effect is much weaker when the compound is added to media after 48 h and interacts only briefly with the luciferase enzymes either in the cytoplasm or the cell lysate, it is possible that NSC158959 interacts with the *Firefly* luciferase enzyme in addition to miR-122 specific effect. We note that similar trends were found in two bidirectional control reporters, when *Renilla* and *Firefly* signals were analyzed separately.

Since NSC158959 and NSC308847 did not meet the criteria for positive assay validation controls, we also tested a published miR-122 inhibitor NSC5476[7] with luciferase reporters. We observed the de-repressive effect of NSC5476 on miR-122 reporters but, similar to NSC158959, we also observed an effect on the control reporter with scrambled binding sites (**Supplementary Fig. 1g**). As before, we repeated the measurements with individual untargeted luciferases and added the compound at different time points. In contrast to NSC158959, this time the *Renilla* luciferase activity is increased when the chemical is incubated with cells for 48 hours (added at 0 hours) (**Supplementary Fig. 1h**), indicating a possible non-specific effect on *Renilla* luciferase and a resulting increase in the ratiometric signal between *Renilla* and *Firefly*. To summarize, in our reporter assays the

reported specific miR-122 effects of some of the chemical modulators might have been obscured by additional non-specific interactions due to changes in the reporter systems and/or cell culture and transfection conditions, suggesting that these compounds may act in complex fashion and making them unsuitable as positive controls in our circuits.

In addition to small-molecule effectors we sought genetic or RNAi based modulators of miR-122. Literature reports showed that Exportin-5, the protein responsible for nuclear pre-miRNA export, increases the efficiency of miRNA strength for let-7a in HEK293 cells[9] and that targeting of Drosha[1], DGCR8[1, 10], Dicer[1, 3] and TRBP2[1] by siRNA or shRNA leads to a reduction of certain mature miRNAs. We assayed the effects of Exportin-5, siDicer0 and anti-DGCR8-shRNA on four different candidate miRNAs using their respective bidirectional reporters (**Supplementary Fig. 1i**): highly expressed miRNA miR-122, modestly expressed miRNA let-7b, miR-146a expressed at very low levels; and the negative control reporter with a scrambled miRNA binding site. We did not observe a clear effect. For the other siRNAs targeting the miRNA pathway we first searched the literature for published deep-sequencing or microarray data and found a dataset for siDicer in MCF-7 cells[2]. We sorted this dataset for highest differential effects and compared it with the expression strengths we observed with reporters in HuH-7. We identified a set of five miRNAs, which met both criteria, namely miR-18a, 7, let-7b, 16 and 17 and added to this set the miRNA inputs for the non-specific RNAi module, and miR-122. We found three miRNAs that were responsive to some of the siRNAs, namely miR-18a, let-7b and -17 with effects in the range of 2-fold. Unfortunately miR-122 was not among them, ruling out these non-specific modulators for validation (**Supplementary Fig. 1j**).

Next we tried synthetic miRNA mimics and LNA-based miRNA inhibitors[7, 11]. All mimics showed good performance, with miR-146a and miR-141 mimics slightly outperforming miR-145 and miR-375 (**Supplementary Fig. 2a**). Therefore we chose miR-141 and miR-146a as the low inputs for the non-specific RNAi module. Likewise, LNAs against miR-21 and miR-20a resulted in almost-complete inhibition of their cognate miRNAs (**Supplementary Fig. 2b**). To confirm that the mimics and the LNAs can be used together to

simulate different non-specific effects, we also measured their mutual orthogonality whereby each mimic or LNA was tested with all the reporters including their cognate ones. Our data show that there is no significant crosstalk within this set (**Supplementary Fig. 2c, d**).

Supplementary Note 2 for section "Assembly and testing of pilot circuit"

We addressed first the sensing of highly-expressed HuH-7 miRNA inputs. Initial characterization of fully-assembled sensors for these miRNAs[12] in HuH-7 cells did not achieve desired dynamic range. Therefore we established an optimization assay to maximize the effect of rtTA knockdown by miRNA inputs on the downstream rtTA-controlled components, that is, a combination of LacI and miR-FF4 repressors. To quantify this effect, we replaced these repressors with fluorescent reporter mCerulean and cloned the binding sequences complementary to the previously-identified high input candidates miR-21 and miR-20a in the 3'-UTR of the rtTA activator. As a control we used rtTA with scrambled miRNA binding site (TFF5). We kept the reporter constant and varied rtTA levels in transient transfections, measuring mCerulean. We found that in HuH-7 cells, miR-21 and miR-20a sensors cause enough rtTA knockdown to be reflected in substantially-lower mCerulean levels (**Supplementary Fig. 2e**). As reported in the Main Text, we planned to set up an interface between the specific and non-specific RNAi modules using transcriptional transactivator. The idea was to place this activator as the immediate output of the non-specific module and use it to control two fluorescent reporters via a bidirectional promoter. One of them would be correlated with the non-specific RNAi module activity and thus represent the non-specific readout, while the second would be additionally furnished with the binding sequence for miR-122 (**Fig. 1c**). The ratio between the latter and the former reporters would indicate specific effect on miR-122 regardless of non-specific interaction, and help isolate this specific effect. Since rtTA is used in high-level miRNA sensors, we tested other known engineered transactivators that function in mammalian cells, namely the Streptogramin-responsive transactivator (Pristinamycin- induced protein (Pip) fused to p65;

PIT2)[13] and the erythromycin-dependent transactivator (MphR(A) fused to VP16; ET)[14]. We constructed bidirectional promoters for these activators driving mCerulean and mCherry and compared their dose response with well-characterized bidirectional pTRE promoter (**Supplementary Fig. 2f**). Protein coexpression was found to be comparable to pTRE (**Supplementary Fig. 2g**), while absolute expression was lower. PIT2 dose-response was more gradual compared to ET. Next, we cloned these transactivators, linked to mCitrine reporter via a 2A peptide[15], as the immediate AND gate outputs of the non-specific RNAi module. This involved placing them under CAGop promoter and furnishing them with the binding sequences for low miRNA inputs, miR-141 and miR-146a. We then measured how their regulation via low-input and high-input sensors translates into the levels of downstream bidirectional reporters mCerulean and mCherry, with mCherry additionally furnished with miR-122 binding sequence in its 3'-UTR, to reflect specific miR-122 knockdown.

First, we cotransfected the transactivator and the bidirectional reporter constructs at optimal concentrations and applied miR-141, miR-146a mimics and miR-FF4 siRNAs to assess the direct knockdown of the activator using mCitrine as a proxy, and the indirect effect on activator-induced reporters using mCerulean (**Supplementary Fig. 2h**). To investigate context dependency for miRNA binding sequences, we also tested two sequence arrangements to achieve optimal knockdown. We found that the context matters: in cases where TFF4 binding sequence is adjacent to the stop codon, its cognate siRNA is very potent, but binding sequences following TFF4 are either not affected at all by their mimics in the case of Citrine-2A-PIT2 or affected weakly with ET-2A-Citrine (**Supplementary Fig. 2h**). By swapping the binding site positions and placing TFF4 downstream from other binding sites, we strongly increase the knockdown by the other two mimics while maintaining relatively strong knockdown with siFF4. When comparing the two transactivators we observe strong direct knockdown of the PIT2 as well as strong reduction in PIT2-controlled reporter, while with ET the effect does not propagate downstream. We explain this behavior by hypersensitivity of pERE to ET leading to a strong induction of the promoter also with only very low amounts of transactivator (**Supplementary Fig. 2f**), and thus use only PIT2 in

subsequent experiments. Next, following initial optimizations, we assembled the complete sensors for highly expressed markers using optimal amounts of rtTA constructs augmented with appropriate functional miRNA binding sequences (**Supplementary Fig. 2i**). We then titrated the downstream pTRE-LacI-Tx-miR-FF4 repressor constructs and measured sensor performance for both miR-21 and miR-20a sensors individually in order to maximize sensor dynamic range and sensitivity for changes in high inputs. The dynamic range was measured relative to the constitutive Off sensor whose components are not targeted by input miRNAs. We find that in this Off sensor, potent mCitrine and mCerulean output repression is observed with small amount of the repressor constructs, giving tight Off-state. The On-state obtained with miRNA-targeted sensors also decreases with increasing repressor amount (**Supplementary Fig. 2i**). To maintain high On-state for the purpose of robust assay readouts, we decided to use 1:1:1 plasmid ratio even though it does not result in highest dynamic range.

Supplementary Note 3 for section "Alternative assay design and simulations"

Model overview

A mechanistic model was built using MATLAB SimBiology tool, with all the transcription-translation-degradation reactions, as well as transcription factor binding, modeled explicitly with mass action kinetics. Transcription factor binding was modeled as a non-cooperative process for simplicity, since the factors used in the assay (rtTA, PIT2, LacI) are not known to have high Hill coefficients. Degradation by RNAi was modeled using Michaelis-Menten kinetics, assuming a finite pool of RISC complexes as well as a background pool of microRNAs competing for RISC binding. Transient transfection was modeled explicitly by setting initial gene copy numbers and including a degradation process for transfected genes with the rate constant corresponding to cell doubling every 24 hours. Thus, the network outputs never reach steady state, as is the case in transient transfections. We created the model such that each of the four assay variants can be tested by inactivating

certain reactions and activating others. Because the assays only differ in a small number of such reactions, the modifications affect only a small fraction of the model and in any case they do not require introduction of new parameters. Therefore all four topologies can be compared with the exact same parameter set. We used concentration units of molecules per cell (mpc) with 1 nM = 1000 mpc (an approximate conversion for a mammalian cell). The entire SimBiology project file is enclosed as Supplementary Material. The diagram is show in **Supplementary Figure 3**.

Initial choice of parameters

Despite the large number of reactions, we used similar parameter values to describe identical processes such as transcription, translation and degradation rates as well as the kinetic parameters for RNA interference. The following numerical values were chosen based on literature.

RNAi parameters and modeling

Downregulation of gene expression by miRNA was modeled exclusively at the mRNA level because the target sequences in 3'-UTR are fully complementary. The equation used was

$$\frac{d[RNA]}{dt} = -\frac{k_{cat}[RISC \cdot miRX][RNA]}{K_M + [RISC \cdot miRX]}$$

The initial parameter values[16, 17] were chosen as K_M = 1 nM (1000 molecules/cell) and k_{cat} = 0.017 s^{-1}. The creation of RISC complex associated with a specific miRNA was modeled as a reversible binding reaction with dissociation rate of 0.001 s^{-1} and dissociation constant of about 0.3 nM, leading to estimated association rate constant of 3×10^{-6} s^{-1} mpc^{-1}. The level of cytoplasmic RISC complex was set initially at 3 nM (3000 mpc)[18], although the number refers to the level in HeLa extracts and is thus a probable underestimation of the cytoplasmic protein concentration. For the ratio between the number of mRNA transcripts and the number of spliced microRNA molecules we used the factor 0.4[19].

Transcriptional regulation parameters and modeling

Initially we chose dissociation constants of 1 nM (1000 mpc) for all transcription factors rtTA, LacI and PIT2; given that dissociation rate from an occupied promoter is on the order of 0.01 s^{-1}, the association rate is 10^{-5} $s^{-1}mpc^{-1}$. Transcriptional regulation was modeled explicitly as binding and unbinding reactions without cooperativity; to avoid accumulation of protein-DNA bound complex, such complexes were converted to DNA with the rate that equaled free protein degradation rate.

DNA, RNA and protein synthesis and degradation

Genetic components of the assay are given initial copy numbers on the order of 5-15 copies/cell for the low-level components and the rest scaled by the ratios used in a particular transfection, that is, 1x of rtTA and LacI^FF4^ sensor-encoding genes, 2x of CAGop-driven gene and 4x of the bidirectional PIT2-controlled cassette. Gene copy number is diluted with half-life of 24 hours corresponding to cell division time (k_{deg}=8.02*10^{-6} s^{-1}). RNA synthesis was modeled explicitly with rate of k_{RNA}=0.003 s^{-1} for all species in the model. RNA degradation was modeled with rate constant of k_{deg}^{RNA}=6*10^{-5} s^{-1} corresponding to half-life of 3 hours. Protein translation from mRNA was modeled explicitly with rate constant of 0.008 s^{-1} for all species in the model. Degradation of transcription factor proteins (rtTA, LacI and PIT2) was modeled with rate constant of 2.4*10^{-5} s^{-1} corresponding to half-life of 8 hours. Degradation of stable fluorescent proteins was determined by cell division with rate constant of k_{deg}=8.02*10^{-6} s^{-1}.

Initial parameter scanning

We evaluated the dynamic range of the non-specific RNAi module output in different assays using simulated outputs for three different conditions. The first is denoted (0,0) – this is the condition when all the non-specific inputs are absent (Condition 5 in **Fig. 1d**). Assay should generate low output in this situation. The second condition is (1,0) – both high inputs are present at 1000 mpc each and the low inputs are absent (Condition 0 in **Fig. 1d**). The output

is expected to be high. Third condition is (0,1): the high inputs are absent and two low inputs are present at 100 mpc each (Condition not tested experimentally). The output is expected to be very low. The lower dynamic range is always the one between (1,0) and (0,0) because the (0,0) output is always higher than (0,1).

To get an idea about the parameter space, we ran the simulation (simulation time 180,000 seconds = 50 hours, approximate waiting time from transfection till measurement) while randomizing parameters describing the gene copy numbers, all RNAi-related rate constants and transcription factor association rate constants (eight parameters in total) within approximately 10-fold range. The main criterion to judge different sets was our experimental observation that the (1,0):(0,0) ratio of an assay whose output is fluorescent protein (as is the non-specific ZsYellow output in Parallel assay layout) is on the order of 15-20. Therefore, we ran about 1000 simulations with randomized parameter sets and selected those sets that generated (1,0):(0,0) ratio above 13. The following is the summary of the parameter values that resulted in such ratio:

Parameter name	mean	stdev
Plasmid copy number	6.88	4.7
[RISC complex], mpc	5.60×10^{3}	2.02×10^{3}
mRNA degradation by RISC, K_M, mpc	5.16×10^{2}	2.25×10^{2}
mRNA degradation by RISC, k_{cat}, s^{-1}	3.37×10^{-2}	0.77×10^{-2}
Association of miRNA with RISC, s^{-1} mpc^{-1}	3.78×10^{-6}	1.91×10^{-6}
k_{ON}, rtTA to DNA	6.33×10^{-6}	3.38×10^{-6}
k_{ON}, LacI to DNA	1.49×10^{-5}	8.34×10^{-6}
k_{ON}, PIT2 to DNA	1.40×10^{-5}	7.89×10^{-6}

One observes that the main changes relative to initial parameter values occurred in the parameters related to RNAi pathway: the concentration of RISC complex was higher than

initial estimate, while the K_M was lower and k_{cat} was higher, indicating that RNAi activity is stronger than initial estimates obtained predominantly in cell lysates.

Relative assay performance depends on parameter values

We replaced the initial values in our simulation with the values from the table above and scanned individual parameters across two orders of magnitude. We did not change the initial values of the association rate constants for transcription factor binding because the values in the table were very similar to 10^{-5} $s^{-1}mpc^{-1}$. We plot the ratios (1,0):(0,0) and (1,0):(0,1) for all four assays, as well as these ratios normalized internally to that of the Pilot Assay.

References

1. Mori, M. et al. Hippo Signaling Regulates Microprocessor and Links Cell-Density-Dependent miRNA Biogenesis to Cancer. *Cell* **156**, 893-906 (2014).
2. Friedländer, M.R., Mackowiak, S.D., Li, N., Chen, W. & Rajewsky, N. miRDeep2 accurately identifies known and hundreds of novel microRNA genes in seven animal clades. *Nucleic Acids Research* **40**, 37-52 (2012).
3. Levy, C. et al. Lineage-Specific Transcriptional Regulation of DICER by MITF in Melanocytes. *Cell* **141**, 994-1005 (2010).
4. Rinaudo, K. et al. A universal RNAi-based logic evaluator that operates in mammalian cells. *Nat Biotech* **25**, 795-801 (2007).
5. Shan, G. et al. A small molecule enhances RNA interference and promotes microRNA processing. *Nat Biotech* **26**, 933-940 (2008).
6. Watashi, K., Yeung, M.L., Starost, M.F., Hosmane, R.S. & Jeang, K.-T. Identification of Small Molecules That Suppress MicroRNA Function and Reverse Tumorigenesis. *Journal of Biological Chemistry* **285**, 24707-24716 (2010).
7. Young, D.D., Connelly, C.M., Grohmann, C. & Deiters, A. Small Molecule Modifiers of MicroRNA miR-122 Function for the Treatment of Hepatitis C Virus Infection and Hepatocellular Carcinoma. *Journal of the American Chemical Society* **132**, 7976-7981 (2010).
8. Rogers, S., Wells, R. & Rechsteiner, M. Amino acid sequences common to rapidly degraded proteins: the PEST hypothesis. *Science* **234**, 364-368 (1986).
9. Diederichs, S. et al. Coexpression of Argonaute-2 enhances RNA interference toward perfect match binding sites. *Proceedings of the National Academy of Sciences* **105**, 9284-9289 (2008).
10. Chien, C.-H. et al. Identifying transcriptional start sites of human microRNAs based on high-throughput sequencing data. *Nucleic Acids Research* **39**, 9345-9356 (2011).

11. Connelly, C.M., Thomas, M. & Deiters, A. High-Throughput Luciferase Reporter Assay for Small-Molecule Inhibitors of MicroRNA Function. *Journal of Biomolecular Screening* **17**, 822-828 (2012).

12. Xie, Z., Wroblewska, L., Prochazka, L., Weiss, R. & Benenson, Y. Multi-input RNAi-based logic circuit for identification of specific cancer cells. *Science* **333**, 1307-1311 (2011).

13. Weber, W., Kramer, B.P., Fux, C., Keller, B. & Fussenegger, M. Novel promoter/transactivator configurations for macrolide- and streptogramin-responsive transgene expression in mammalian cells. *The Journal of Gene Medicine* **4**, 676-686 (2002).

14. Weber, W. et al. Macrolide-based transgene control in mammalian cells and mice. *Nat Biotech* **20**, 901-907 (2002).

15. Donnelly, M.L.L. et al. The 'cleavage' activities of foot-and-mouth disease virus 2A site-directed mutants and naturally occurring '2A-like' sequences. *Journal of General Virology* **82**, 1027-1041 (2001).

16. Haley, B. & Zamore, P.D. Kinetic analysis of the RNAi enzyme complex. *Nature Structural & Molecular Biology* **11**, 599-606 (2004).

17. Martinez, J. & Tuschl, T. RISC is a 5 ' phosphomonoester-producing RNA endonuclease. *Genes & Development* **18**, 975-980 (2004).

18. Brown, K.M., Chu, C.Y. & Rana, T.M. Target accessibility dictates the potency of human RISC. *Nature Structural & Molecular Biology* **12**, 469-470 (2005).

19. Strovas, T.J., Rosenberg, A.B., Kuypers, B.E., Muscat, R.A. & Seelig, G. MicroRNA-Based Single-Gene Circuits Buffer Protein Synthesis Rates against Perturbations. *ACS Synthetic Biology* (2014).
